# Schiff Base Ancillary Ligands in Bis(diimine) Copper(I) Dye-Sensitized Solar Cells

**DOI:** 10.3390/ijms21051735

**Published:** 2020-03-03

**Authors:** Elias Lüthi, Paola Andrea Forero Cortés, Alessandro Prescimone, Edwin C. Constable, Catherine E. Housecroft

**Affiliations:** Department of Chemistry, University of Basel, BPR 1096, Mattenstrasse 24a, CH-4058 Basel, Switzerland; elias.luethi@stud.unibas.ch (E.L.); paolaandrea.forerocortes@unibas.ch (P.A.F.C.); alessandro.prescimone@unibas.ch (A.P.); edwin.constable@unibas.ch (E.C.C.)

**Keywords:** copper, dye-sensitized solar cell, Schiff base, phosphonic acid, crystal structure

## Abstract

Five 6,6′-dimethyl-2,2′-bipyridine ligands bearing *N*-arylmethaniminyl substituents in the 4- and 4′-positions were prepared by Schiff base condensation in which the aryl group is Ph (**1**), 4-tolyl (**2**), 4-*^t^*BuC_6_H_4_ (**3**), 4-MeOC_6_H_4_ (**4**), and 4-Me_2_NC_6_H_4_ (**5**). The homoleptic copper(I) complexes [CuL_2_][PF_6_] (L = **1**–**5**) were synthesized and characterized, and the single crystal structure of [Cu(**1**)_2_][PF_6_]·Et_2_O was determined. By using the “surfaces-as-ligands, surfaces-as-complexes” (SALSAC) approach, the heteroleptic complexes [Cu(**6**)(L_ancillary_)]^+^ in which **6** is the anchoring ligand ((6,6′-dimethyl-[2,2′-bipyridine]-4,4′-diyl)bis(4,1-phenylene))bis(phosphonic acid)) and L_ancillary_ = **1**–**5** were assembled on FTO-TiO_2_ electrodes and incorporated as dyes into n-type dye-sensitized solar cells (DSCs). Data from triplicate, fully-masked DSCs for each dye revealed that the best-performing sensitizer is [Cu(**6**)(**1**)]^+^, which exhibits photoconversion efficiencies (*η*) of up to 1.51% compared to 5.74% for the standard reference dye N719. The introduction of the electron-donating MeO and Me_2_N groups (L_ancillary_ = **4** and **5**) is detrimental, leading to a decrease in the short-circuit current densities and external quantum efficiencies of the solar cells. In addition, a significant loss in open-circuit voltage is observed for DSCs sensitized with [Cu(**6**)(**5**)]^+^, which contributes to low values of *η* for this dye. Comparisons between performances of DSCs containing [Cu(**6**)(**1**)]^+^ and [Cu(**6**)(**4**)]^+^ with those sensitized by analogous dyes lacking the imine bond indicate that the latter prevents efficient electron transfer across the dye.

## 1. Introduction

The Grätzel n-type dye-sensitized solar cell (DSC) was developed in the early 1990s and converts solar to electrical energy [1,2,3,4]. A coating of nanoparticles of a semiconductor (usually TiO_2_) on a transparent conducting oxide glass electrode is treated with a dye to form the photoanode of the DSC. The performance of the cell depends on a number of factors, most crucially the dye, the redox couple in the electrolyte, and the electrolyte composition. Over the last 25 years, much effort has gone into optimizing these components in order to improve the overall performance efficiency. Typical dyes include metal-free organic, ruthenium(II) and zinc(II) porphyrin compounds, and the highest photoconversion efficiencies (*η*) now reach 12–14% [5,6,7,8,9]. The structures of many organic dyes are complex and the associated synthetic strategies and protocols are non-trivial. Thus, the use of metal coordination compounds remains attractive, as they are both photostable and relatively easy to prepare, provided that ligand design and synthesis can be optimized. Although ruthenium(II) dyes represent state-of-the-art, the use of first row *d*-block metals such as copper [10,11,12,13] or iron [14,15,16,17] is advantageous in terms of sustainability and conforming to the United Nations Sustainable Development Goals (SDGs). 

**Scheme 1:**
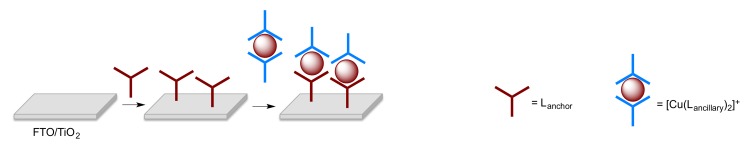
Schematic representation of the SALSAC ligand-exchange strategy for heteroleptic copper(I) dye assembly.

The complexes used as dyes possess ligands that covalently bind to the surface of the semiconductor (the anchoring ligand(s)) and additional ligands, which are used to modify the photonic and electronic properties (the ancillary ligand(s)). In order to achieve efficient electron injection into the semiconductor in a DSC, a dye should exhibit a “push–pull” (or donor–π–acceptor, D–π–A) design, in which the ancillary ligands possess π-donor properties and the anchoring ligands π-acceptor character [18,19,20]. Homoleptic bis(diimine)copper(I) complexes are easily synthesized from reactions of [Cu(MeCN)_4_]^+^ salts with a 2,2′-bipyridine (bpy) or 1,10-phenanthroline (phen) ligand, but they lack the necessary “push–pull” character. In solution, heteroleptic bis(diimine)copper(I) compounds tend to undergo ligand redistribution leading to mixtures of homo- and heteroleptic species. Two different strategies have been devised to assemble and stabilize heteroleptic bis(diimine)copper(I) complexes for use in DSCs. The HETPHEN (HETeroleptic PHENanthroline) approach utilized by Odobel and coworkers [21,22] employs sterically demanding substituents in the 6,6′-positions of a bpy ligand, or in the 2,9-positions of phen, to isolate heteroleptic [Cu(L)(L′)][X] complexes and stabilize them with respect to ligand redistribution in solution. In contrast, we have adopted the “surfaces-as-ligands, surfaces-as-complexes” (SALSAC) approach [23,24], which is extremely versatile and allows heteroleptic copper(I) dyes to be assembled directly on a semiconductor surface by ligand exchange between an anchored diimine ligand and a homoleptic copper(I) complex (Scheme 1). 

**Scheme 2:**
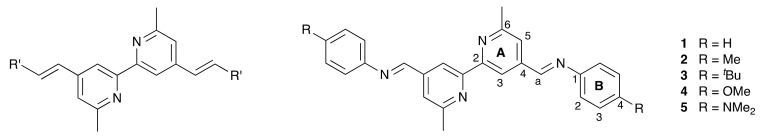
The general ligand type derived from 4,4′-styryl-6,6′-dimethyl-2,2′-bipyridine previously investigated (left), and the structures of compounds **1**–**5** with atom labeling for NMR spectroscopic assignments.

A class of ligands that has proven beneficial in bis(diimine)copper(I) dyes is based upon 4,4′-styryl-6,6′-dimethyl-2,2′-bipyridine in which the R′ group in Scheme 2 is systematically varied. Complexes in which R′ = 4-(^n^Bu_2_NC_6_H_4_) or 4-[^n^(C_8_H_17_)_2_NC_6_H_4_] exhibit absorption spectra dominated by intense intraligand charge-transfer (ILCT) bands with the metal-to-ligand charge-transfer (MLCT) absorption appearing as a low-energy shoulder [25]. With R′ = CO_2_H, we incorporated the ligands on the left-side of Scheme 2 into homoleptic complexes and established functioning copper(I) dyes in DSCs [26]. The extended conjugation in this family of ligands increases the intensity and results in a red-shift of the MLCT absorption, both of which are beneficial for light-harvesting [26,27]. Daniel, Odobel, and coworkers applied the HETPHEN strategy to incorporate ligands derived from 4,4′-styryl-6,6′-dimethyl-2,2′-bipyridine into heteroleptic copper(I) complexes [27] and followed this with an impressive demonstration of the potential for these dyes in DSCs [22]. Among metal-free organic dyes, a few containing azo spacers have been shown to be effective sensitizers in DSCs [28]. Between the extremes of the C=C and N=N units lies the imine bond, and we were interested to see how heteroleptic copper(I) dyes incorporating Schiff base ligands would perform in DSCs. The ease of preparation of Schiff bases by the condensation of amines with aldehydes or ketones has lead to a myriad of applications, including those in energy-related materials [29]. To the best of our knowledge, there are no examples of Schiff base-decorated bpy ligands being used in copper(I) sensitizers. The reversibility of imine bond formation is an attractive feature in terms of the potential for the structural manipulation and chemical regeneration of degraded surface-bound dyes. 

## 2. Results and Discussion

### 2.1. Ligand Synthesis and Characterization

**Scheme 3:**
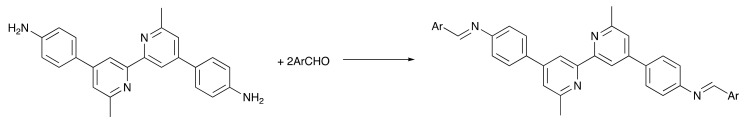
Initial strategy to Schiff base ligands.

Two approaches to the Schiff base ligands **1**–**5** (Scheme 2) were considered. The first was based on the condensation of appropriate aldehydes with 4,4′-bis(4-aminophenyl)-6,6′-dimethyl-2,2′-bipyridine (Scheme 3) [30]. In order to favor the formation of the desired imines, it was necessary to limit the reaction to aldehydes containing no α-protons in order to minimize enamine formation. Typically, the Schiff base products were poorly soluble in common organic solvents, leading to difficulties in purification. Thus, the approach shown in Scheme 3 was abandoned. A second approach employed the condensation of 6,6′-dimethyl-[2,2′-bipyridine]-4,4′-dicarbaldehyde with arylamines, a strategy that is analogous to that used by Le Bozec and coworkers to prepare the compounds shown in Scheme 4 from [2,2′-bipyridine]-4,4′-dicarbaldehyde [31]. 6,6′-Dimethyl-[2,2′-bipyridine]-4,4′-dicarbaldehyde was prepared according to the route in Scheme 5, following a literature procedure [32]. Subsequent condensations of the dialdehyde with aniline, 4-methylaniline, 4-*tert*-butylaniline, 4-methoxyaniline, and 4-(dimethylamino)aniline gave ligands **1**–**5** in yields ranging between 55.0% and 70.1%.

**Figure 1:**
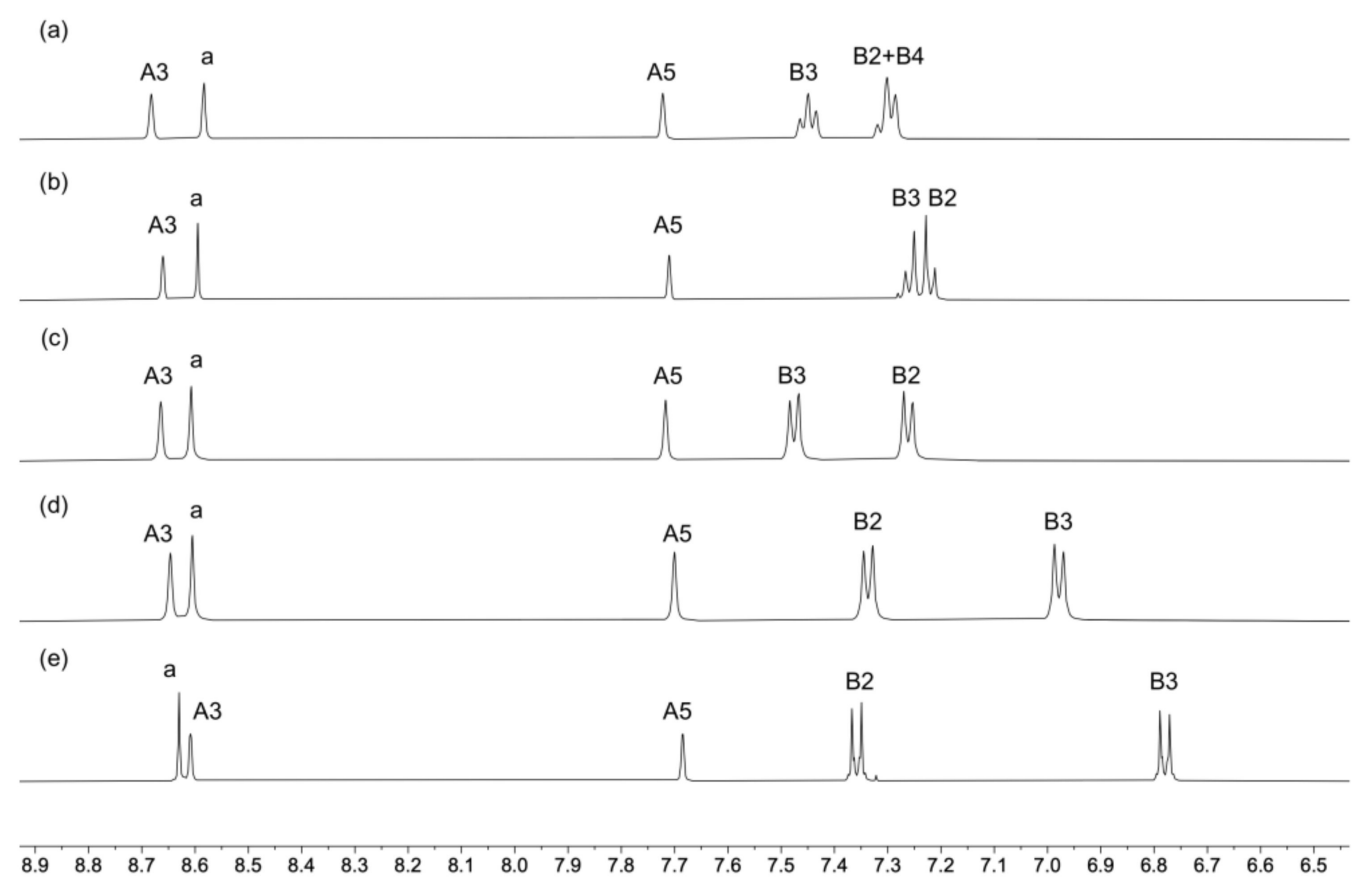
The aromatic regions of the ^1^H NMR (500 MHz, CD_2_Cl_2_, 298 K) spectra of compounds (**a**) 1, (**b**) 2, (**c**) 3, (**d**) 4, and (**e**) 5. See Scheme 2 for atom labeling.

Compounds **1**–**5** were characterized by NMR, IR, and UV–vis spectroscopies and high-resolution electrospray mass spectrometry (HR ESI–MS). Peaks corresponding to [M+H]^+^ were observed in the mass spectra (see Materials and Methods section). IR spectra are shown in Figures S1–S5 (see the supporting information). The ^1^H and ^13^C{^1^H} NMR spectra were assigned using NOESY, COSY, HMQC, and HMBC spectra. The aromatic regions of the ^1^H NMR spectra are displayed in Figure 1 and full spectra in Figure S6. Figures S7–S16 show the HMQC and HMBC spectra of compounds **1–5**. Protons H^B2^ and H^B3^ were distinguished in each of ligands **2**, **3**, **4**, and **5** by NOESY cross-peaks to the methyl, *tert*-butyl, methoxy and dimethylamino protons, respectively, and the shift to lower frequencies for the signals for protons H^B3^ in **4** and **5** (Figure 1) is consistent with the effects of the electron-donating MeO and Me_2_N groups. 

**Scheme 4:**
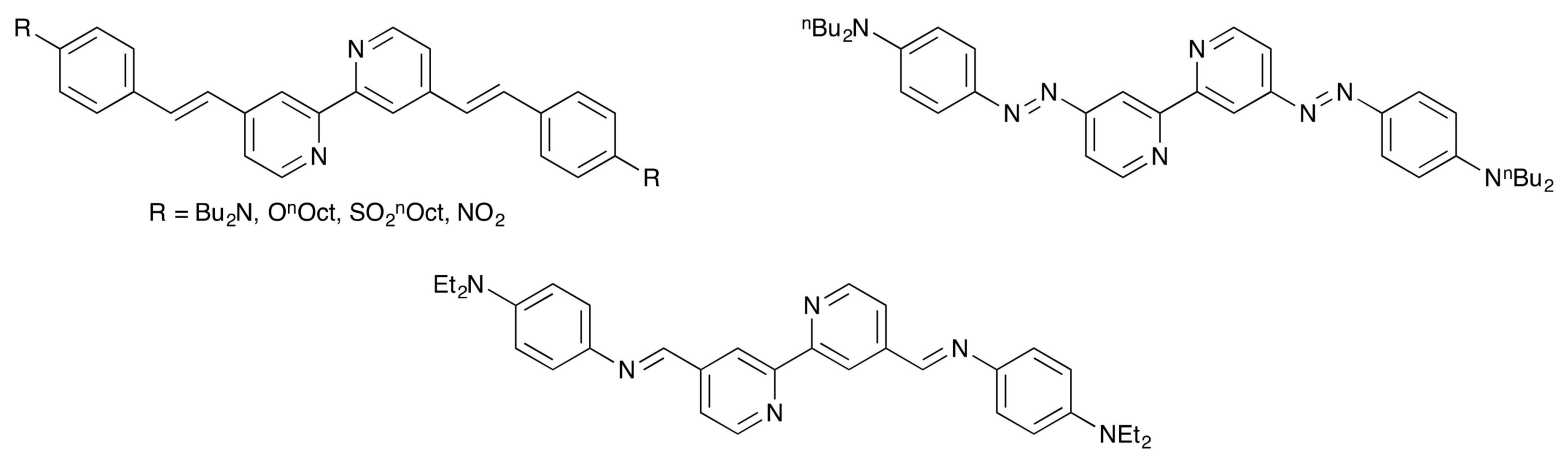
4,4′-π-Conjugated 2,2′-bipyridines, including a Schiff base derivative, reported by Le Bozec and coworkers [31].

**Scheme 5:**
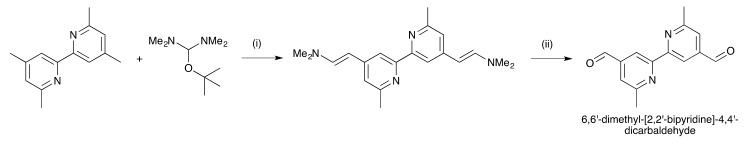
Synthetic route to the precursor 6,6′-dimethyl-[2,2′-bipyridine]-4,4′-dicarbaldehyde. Conditions: (i) DMF, 160 °C, 18 h; 52% yield; (ii) NaIO_4_, THF, 15 h, room temperature; 44% yield.

**Figure 2:**
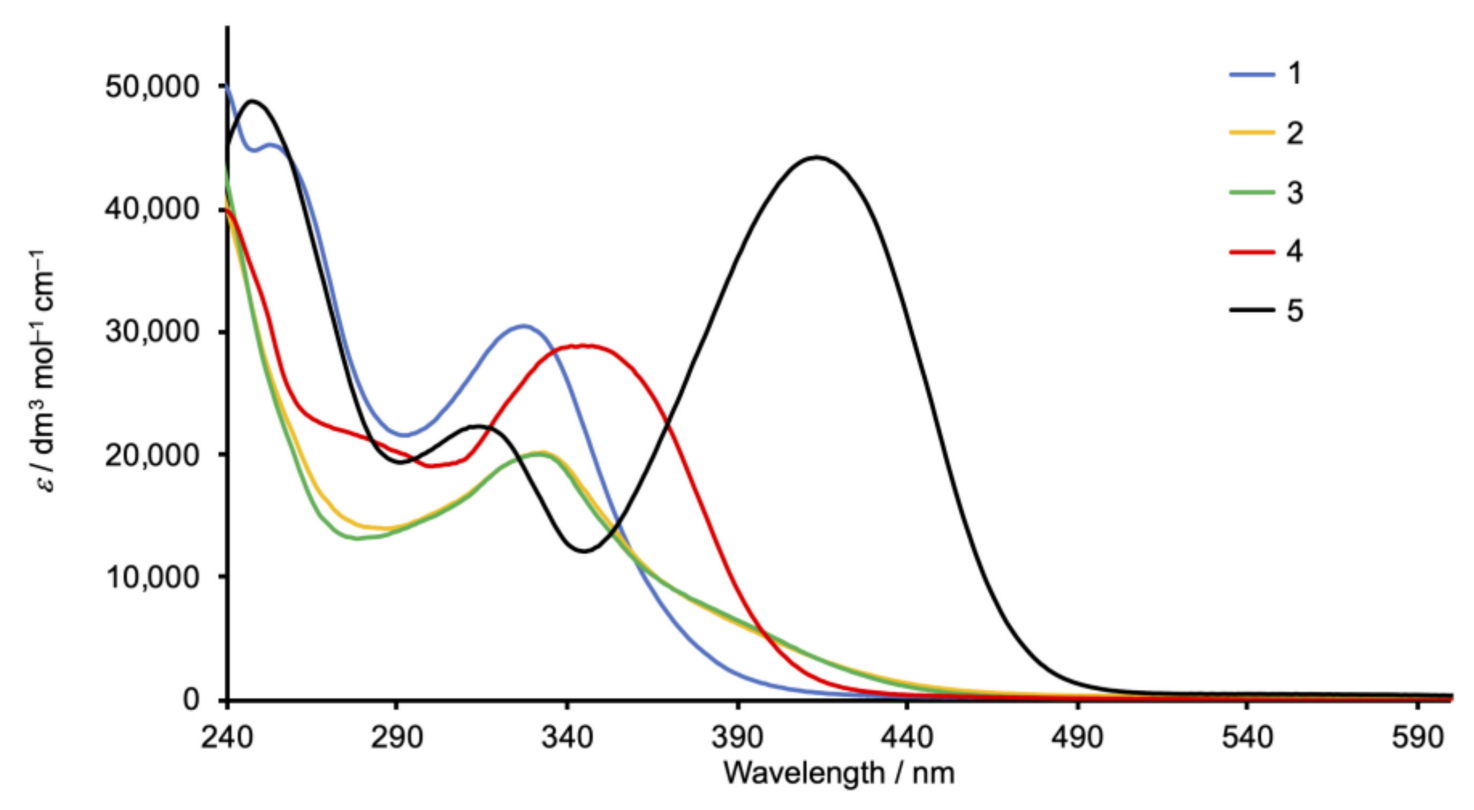
Solution absorption spectra of compounds **1**–**5** (CH_2_Cl_2_, 2 × 10^−5^ mol dm^−3^).

The solution absorption spectra of **1**, **2**, and **3** (Figure 2) recorded in CH_2_Cl_2_ exhibit intense bands below 350 nm arising from spin-allowed, π*←π transitions. In contrast, the spectra of the methoxy- and dimethylamino derivatives **4** and **5** exhibit intense absorption maxima at 344 and 420 nm, respectively, which are assigned to ILCT transitions, consistent with the “push–pull” character of these compounds. The value of *λ*_max_ = 420 nm for **5** compares with *λ*_max_ = 433 nm (also in CH_2_Cl_2_) for the related Schiff base derivative (Scheme 4) reported by Le Bozec [31].

### 2.2. Synthesis and Characterization of the Homoleptic Copper(I) Complexes

**Figure 3:**
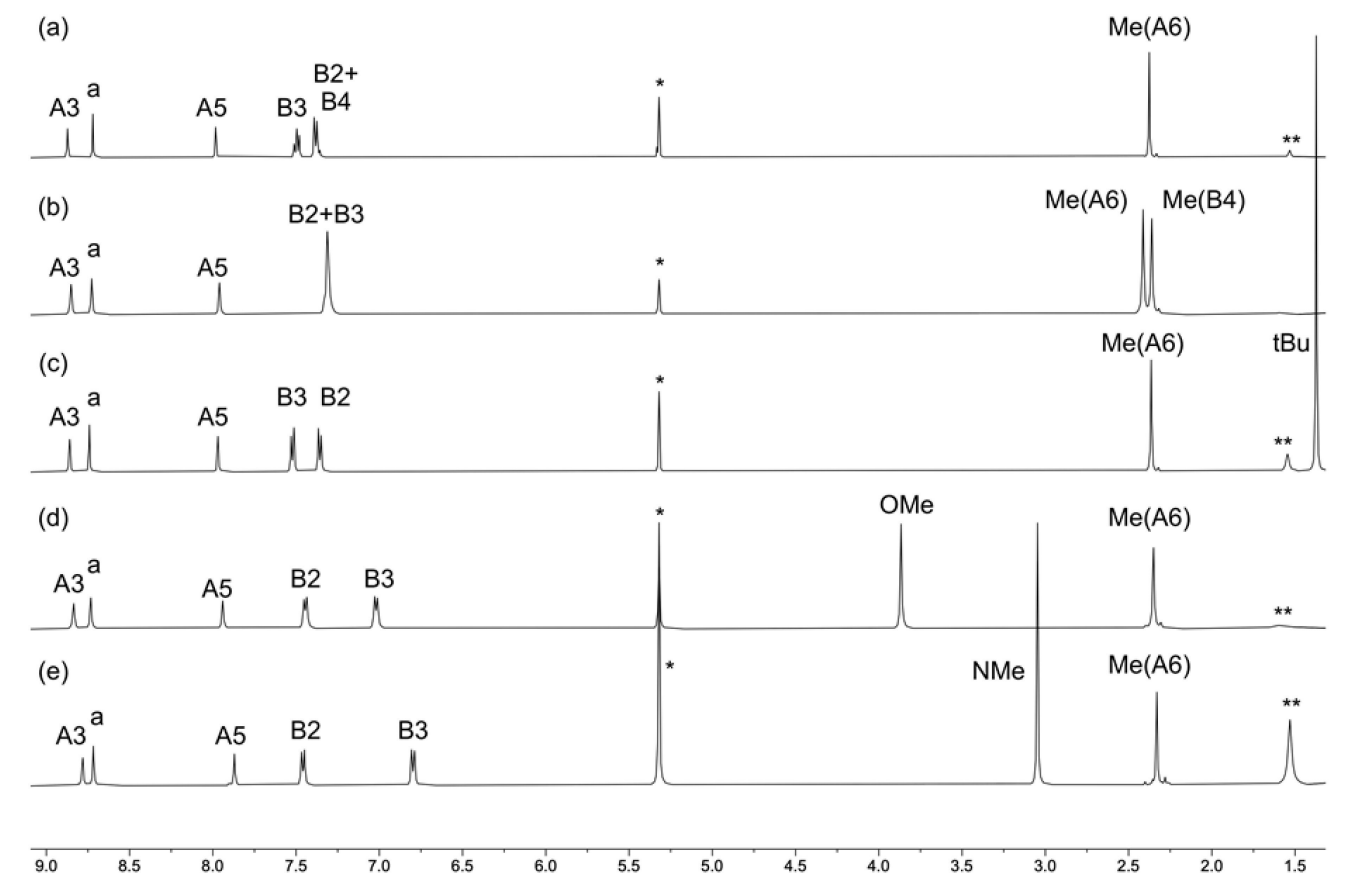
The ^1^H NMR (500 MHz, CD_2_Cl_2_, 298 K) spectra of compounds (**a**) [Cu(**1**)_2_][PF_6_], (**b**) [Cu(**2**)_2_][PF_6_], (**c**) [Cu(**3**)_2_][PF_6_], (**d**) [Cu(**4**)_2_][PF_6_], and (**e**) [Cu(**5**)_2_][PF_6_]. See Scheme 2 for atom labeling. * = Residual CHDCl_2_, ** = H_2_O.

Reactions of [Cu(MeCN)_4_][PF_6_] with two equivalents of **1**, **2**, **3**, **4**, or **5** resulted in the formation of the homoleptic [CuL_2_][PF_6_] complexes in yields of between 70.2% and 86.9% after recrystallization from CH_2_Cl_2_ and Et_2_O. IR spectra of the compounds are displayed in Figures S17–S21 (see the supporting information). Both ESI (Figures S22–S26) and high-resolution ESI mass spectra were recorded and exhibited base peaks arising from the [M–PF_6_]^+^ ions. The ^1^H NMR spectra of the complexes are shown in Figure 3 and are similar to those of the free ligands (Figure 1 and Figure S6). Signal assignments were made using 2D NMR methods, and the HMQC and HMBC spectra are shown in Figures S27–S36 in the supporting information). The ^31^P{^1^H} NMR spectrum of each compound exhibited a septet at *δ* −144.4 ppm characteristic of [PF_6_]^−^.

### 2.3. Crystal Structure of [Cu(1)_2_][PF_6_]·Et_2_O.

**Figure 4:**
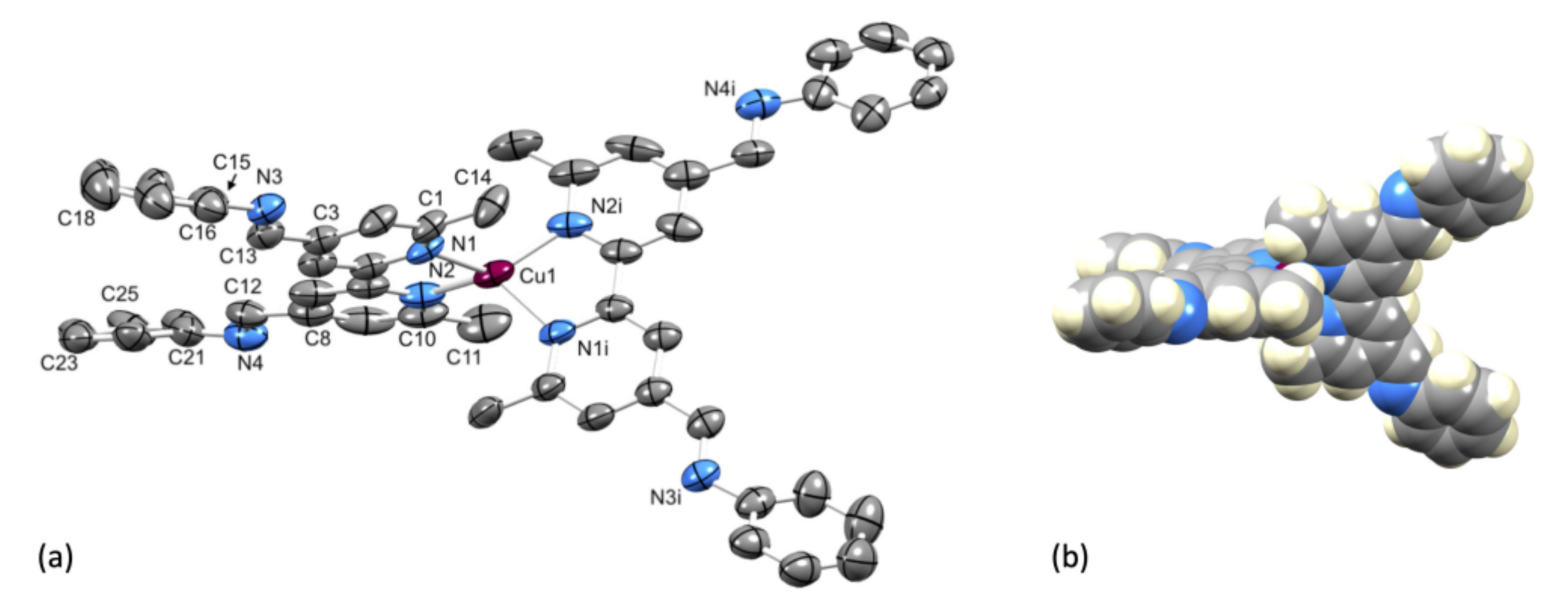
(**a**) ORTEP-style representation of the structure of the [Cu(**1**)_2_]^+^ cation in [Cu(**1**)_2_][PF_6_]·Et_2_O. Ellipsoids are plotted at 40% probability level and H atoms and solvent molecules are omitted for clarity. Important bond metrics: Cu1–N1 = 2.033(5), Cu1–N2 = 2.035(6), N3–C13 = 1.262(9), N3–C15 = 1.441(9), C3–C13 = 1.485(9), N4–C12 = 1.213(8), N4–C21 = 1.425(10), C12–C8 = 1.583(11) Å; N1–Cu1–N2 = 81.2(2), N1–Cu1–N1i = 117.4(3), N1–Cu1–N2i = 126.7(2), C13–N3–C15 = 116.8(7), C12–N4–C21 = 110.9(6)°. (**b**) Space-filling representation of the [Cu(**1**)_2_]^+^ cation illustrating the location of the 6- and 6′-methyl substituents, providing steric protection around the copper(I) center.

**Figure 5:**
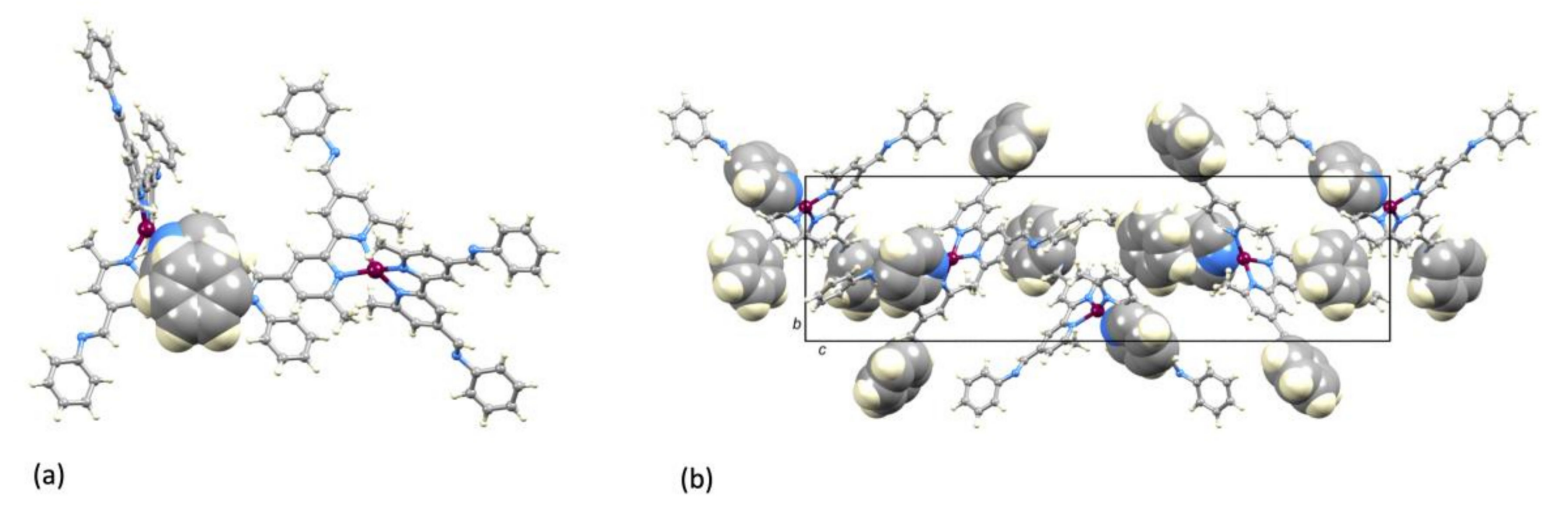
Packing of [Cu(**1**)_2_]^+^ cations in [Cu(**1**)_2_][PF_6_]·Et_2_O. (**a**) Face-to-face π-stacking interaction between the phenyl ring containing atom C21 and the pyridine ring with N1. (**b**) View down the crystallographic *a*-axis showing π-stacking interactions through part of the crystal lattice; all aromatic rings involved in π-stacking are shown in space-filling representation.

Single crystals of [Cu(**1**)_2_][PF_6_]·Et_2_O were grown from a CH_2_Cl_2_ solution of the compound layered with Et_2_O. The compound crystallizes in the tetragonal space group *P*4_1_22 with half of the [Cu(**1**)_2_]^+^ cation and half the [PF_6_]^−^ anion in the asymmetric unit. The second half of each ion is related to the first by a 2-fold rotation axis. Figure 4a shows the structure of the complex cation and selected bond lengths and angles are given in the figure caption. Atom Cu1 is in a distorted tetrahedral coordination environment with the 6- and 6′-methyl substituents of one ligand accommodated over the chelate ring formed by the second bpy unit (Figure 4b). The bpy unit is slightly twisted with an angle of 12.0° between the planes of the pyridine rings. The two C=N bonds lie approximately in the same plane of the pyridine ring to which each is bonded (torsion angles N3–C13–C3–C4 = –174.2(6)° and N4–C12–C8–C7 = −174.2(7)°), while the phenyl rings are twisted out of this plane (torsion angles C16–C15–N3–C13 = 147.0(7)° and C26–C21–N4–C12 = 151.8(7)°). Packing of the [Cu(**1**)_2_]^+^ cations involves face-to-face π-stacking of the phenyl ring containing atom C21 and the pyridine ring with N1 (Figure 5a). The angle between the ring planes is 7.8° and the centroid...centroid distance is 3.84 Å, parameters that are consistent with efficient packing [33]. The inter-cation π-stacking interactions involve the crystallographically independent ligand with its symmetry generated counterpart, and the packing, therefore, extends throughout the lattice, as shown in Figure 5b.

### 2.4. Absorption Spectra and Electrochemical Properties of the Homoleptic Copper(I) Complexes

**Figure 6:**
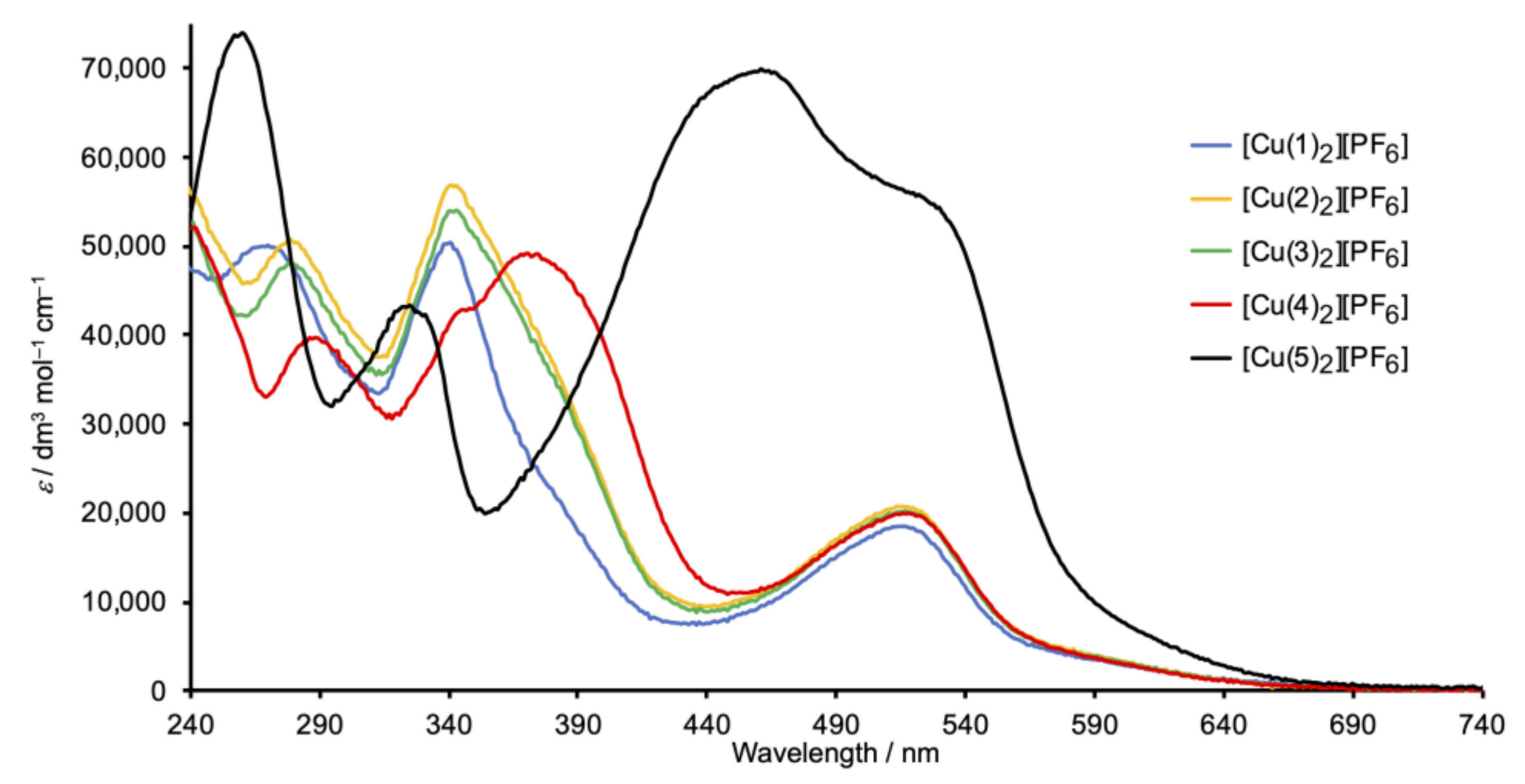
Solution absorption spectra of complexes [CuL_2_][PF_6_] with L = **1**–**5** (CH_2_Cl_2_, 2 × 10^−6^ mol dm^−3^).

Figure 6 depicts the solution absorption spectra of the Schiff base copper(I) complexes. The spectra of [Cu(**1**)_2_][PF_6_], [Cu(**2**)_2_][PF_6_], and [Cu(**3**)_2_][PF_6_] are similar and exhibit high-energy absorptions arising from ligand-centered π*←π transitions, in addition to a broad, lower intensity band at 518, 513, and 516 nm, respectively, from the MLCT. In contrast, the spectrum of [Cu(**4**)_2_][PF_6_] (with the peripheral methoxy functionalization) shows an additional absorption at 370 nm arising from ILCT, consistent with the spectral signature of the free ligand **4** (Figure 2). In [Cu(**5**)_2_][PF_6_], the overlap of the ILCT (461 nm) with the MLCT (shoulder at 530 nm) (compare Figure 2 and Figure 6), and the mixed character of these excited states leads to a significant increase in MLCT intensity and a red-shifting of the absorption. 

**Figure 7:**
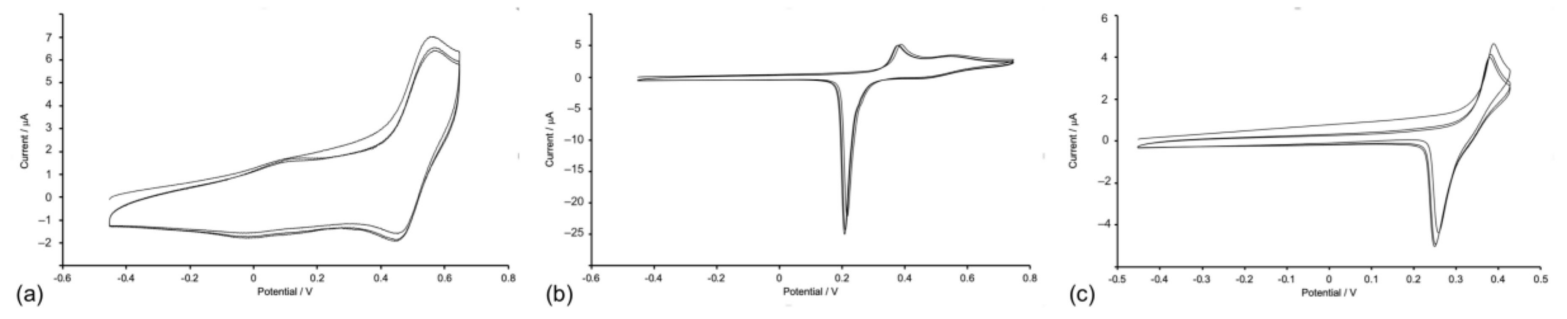
(**a**) CV of [Cu(**1**)_2_][PF_6_] showing three successive cycles scanned between −0.45 and +0.65 V with a glassy carbon working electrode. CVs of [Cu(**5**)_2_][PF_6_] showing three successive cycles scanned between (**b**) −0.45 V and +0.75 V and (**c**) −0.45 V and +0.43 V, with a platinum working electrode. Referenced to external Fc/Fc^+^ = 0.0 V; CH_2_Cl_2_ solution with [*^n^*Bu_4_N][PF_6_] as supporting electrolyte and scan rate of 0.1 V s^−1^.

The copper(I) complexes are redox-active and their electrochemical behavior was investigated by cyclic voltammetry (CV). Table 1 summarizes the electrochemical processes for the copper(I) compounds and also for ligands **2** and **5**. Each of [Cu(**1**)_2_][PF_6_] (Figure 7), [Cu(**2**)_2_][PF_6_], [Cu(**3**)_2_][PF_6_], and [Cu(**4**)_2_][PF_6_] undergoes a reversible copper-centred oxidation at +0.48 or +0.49 V (with respect to ferrocene, Fc/Fc^+^). This potential compares with +0.42 V (vs. Fc/Fc^+^ in CH_2_Cl_2_) for [CuL_2_][PF_6_] where L = 4,4′-bis(4-bromophenyl)-2,2′-bipyridine [34], and +0.35 V for [Cu(6,6′-Me_2_bpy)_2_][TFSI] (Me_2_bpy = 6,6′-dimethyl-2,2′-bipyridine, TFSI = bis(trifluoromethanesulfonyl)imide) [35]. For [Cu(6,6′-Me_2_bpy)_2_][TFSI], the original literature value was +0.97 V vs. SHE, the potential is corrected by −0.62 V to formally adjust to Fc/Fc^+^ [36]. Each compound undergoes a series of irreversible ligand-centered reductions (Table 1). For [Cu(**2**)_2_][PF_6_] and [Cu(**4**)_2_][PF_6_], if the positive potential scan window is taken past +1.07 or +1.20 V, respectively, through a ligand oxidation process, the return wave for the Cu^+^/Cu^2+^ process is lost and a new ECE (electrochemical–chemical–electrochemical) wave is observed at +0.69 or +0.21 V, respectively. For [Cu(**5**)_2_][PF_6_], the Cu^+^/Cu^2+^ oxidation is irreversible and the oxidation occurs an +0.54 V (Figure 7b). This process is preceded by a partially reversible ligand-centered oxidation at +0.38 V (Figure 7b,c), with the return wave showing an adsorption spike. The CV of ligand **5** exhibits an oxidative process at a similar potential to [Cu(**5**)_2_][PF_6_] (Table 1), and we, therefore, assign this to an oxidative process centered on the NMe_2_ group. No analogous process is observed in the CV of ligand **2**, which is representative of ligands with no redox-active substituents.

### 2.5. Solar Cell Fabrication and Performances

**Scheme 6:**
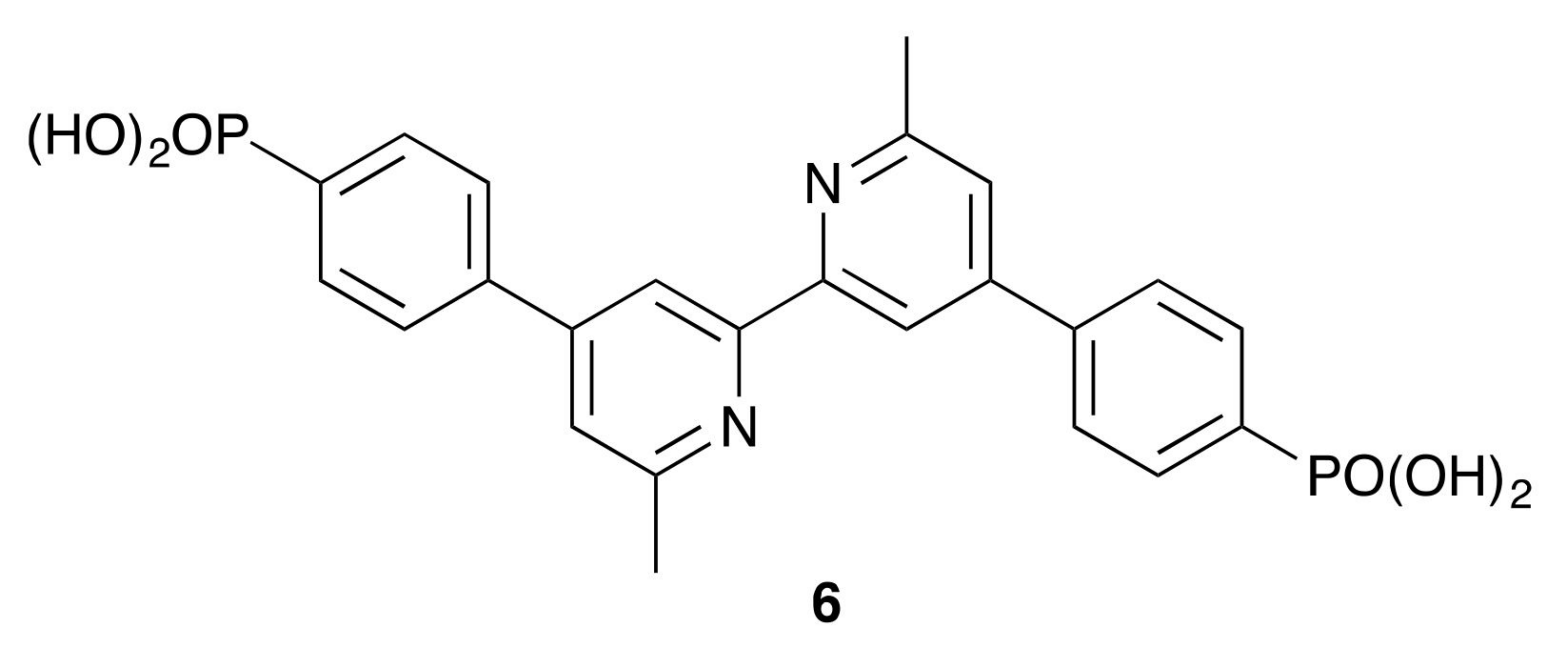
The structure of anchoring ligand **6**.

**Figure 8:**
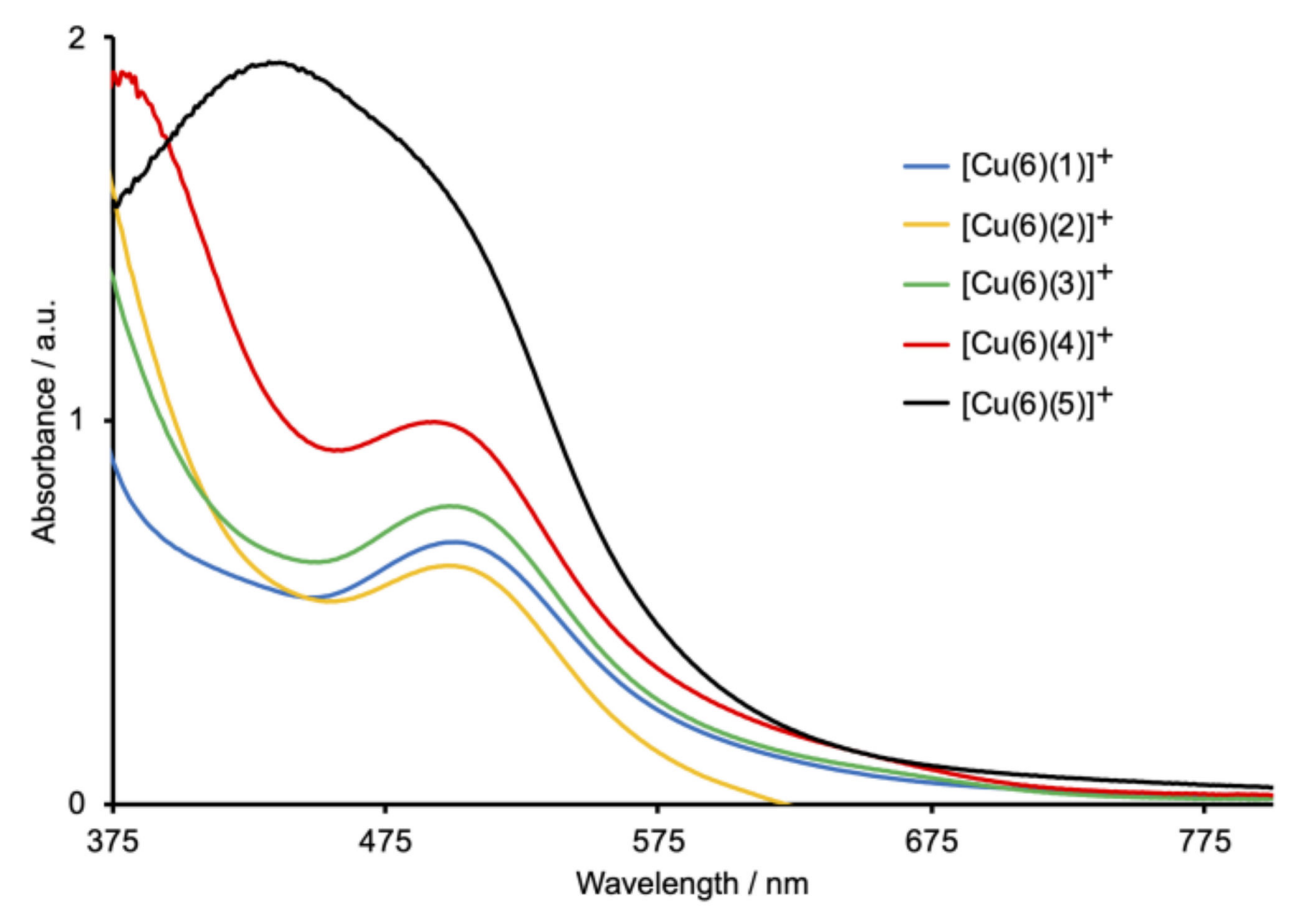
Solid-state absorption spectra of FTO/TiO_2_ electrodes functionalized with the Schiff-base dyes. The highest energy absorption (below ca. 380 nm) is due to TiO_2_.

Heteroleptic [Cu(L_anchor_)(L_ancillary_)]^+^ dyes were assembled on commercial FTO/TiO_2_ electrodes using the SALSAC ligand-exchange strategy [23,24], as depicted in Scheme 1. The phosphonic acid anchoring ligand **6** (Scheme 6) was chosen because it exhibits enhanced binding to TiO_2_ with respect to carboxylic acids [24]. Taking both DSC performance and efficient ligand synthesis into account, we have previously established that copper(I) dyes with phosphonic acid or phosphonate anchors [37] containing a phenylene spacer between the anchor and copper-binding domains are favored over those with carboxylic acid (or carboxylate) and cyanoacrylic acid (or cyanoacrylate) anchors [34,38,39]. The solid-state absorption spectra of the dye-functionalized electrodes are shown in Figure 8. Each of [Cu(**6**)(**1**)]^+^, [Cu(**6**)(**2**)]^+^, and [Cu(**6**)(**3**)]^+^ has an MLCT absorption maximum at 504 nm, while λ_max_ for [Cu(**6**)(**4**)]^+^ is 498 nm. For [Cu(**6**)(**5**)]^+^, the spectrum is dominated by the ILCT absorption (λ_max_ = 438 nm) with a shoulder at ca. 500 nm arising from the MLCT band. Maxima in the solid-state spectra of the heteroleptic dyes (Figure 8) are blue-shifted with respect to those of the homoleptic complexes in solution (Figure 6).

**Figure 9:**
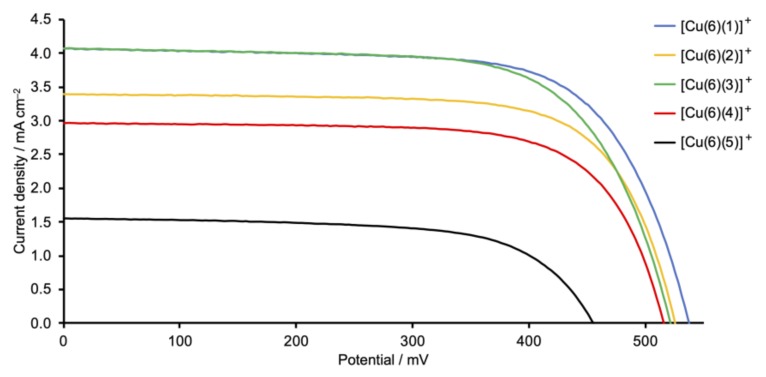
*J–V* curves for DSCs sensitized by copper(I) dyes containing Schiff base ancillary ligands **1–5**. The best-performing cell for each dye is shown; *J–V* curves for all the DSCs are depicted in Figures S38–S41 in the supporting information. Refer to Scheme 2 for the ancillary ligand structures.

The photoconversion efficiencies of the DSCs (sets of three, fully-masked cells for each dye) were measured and cell performance parameters are given in Table 2. For reference, a DSC sensitized with the commercially available ruthenium(II) dye N719 was used. In the right-hand column of Table 2, we give values of the relative efficiency of each DSC relative to the cell with N719 set at 100%. This is a reliable means of comparing DSC parameters recorded, for example, with different sun simulators [40]. The fill-factors (*ff*) of the cells lie in the range 65–70%, consistent with well-fabricated DSCs. The use of triplicate cells confirms the reproducibility of the data, lending confidence to the trends observed in performances as the ancillary ligand is varied. Current density–potential (*J–V*) curves for all the cells are displayed in Figures S37–S41 in the supporting information, and Figure 9 shows *J–V* curves for the best-performing DSC with each dye. 

**Figure 10:**
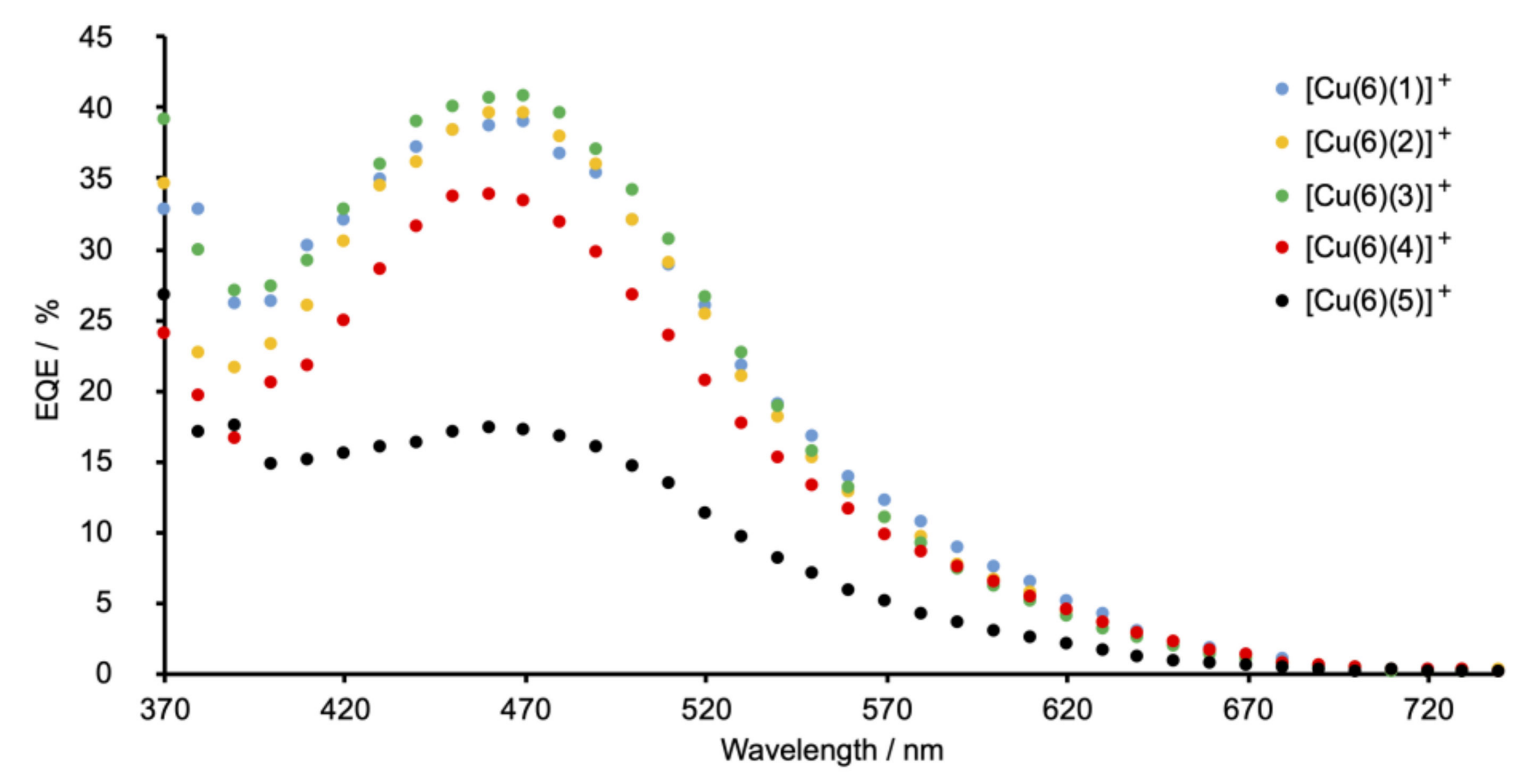
EQE spectra for DSCs sensitized by copper(I) dyes containing Schiff base ancillary ligands **1–5**. The best-performing cell for each dye is shown; EQE spectra for all the DSCs are shown in Figures S42–S46 in the supporting information.

The highest values of the short-circuit current density (*J*_SC_) are observed for DSCs sensitized with [Cu(**6**)(**1**)]^+^ (phenyl substituent) and [Cu(**6**)(**3**)]^+^ (*tert*-butyl substituent). Changing from a phenyl to a 4-methylphenyl group (ancillary ligand **2**) results in a decrease in *J*_SC_ (Table 2 and Figure 9). The differences in *J*_SC_ are consistent with the EQE (external quantum efficiency) spectra (Figure 10). Although values of EQE_max_ are similar for DSCs with [Cu(**6**)(**1**)]^+^, [Cu(**6**)(**2**)]^+^, and [Cu(**6**)(**3**)]^+^ (EQE_max_ ranges from 39% to 41% at λ_max_ = 470 nm), the quantum efficiency is lower for [Cu(**6**)(**2**)]^+^ at wavelengths between 370 and 420 nm than for [Cu(**6**)(**1**)]^+^ and [Cu(**6**)(**3**)]^+^. For all DSCs containing [Cu(**6**)(**1**)]^+^, [Cu(**6**)(**2**)]^+^, and [Cu(**6**)(**3**)]^+^, values of the open-circuit voltage lie in a similar range (518–538 mV). The photoconversion efficiencies (*η*) for dyes containing the Schiff base ancillary ligands with peripheral phenyl, 4-methylphenyl and *tert*-butyl substituents lie in a similar range (*η* = 1.26–1.55%) and achieve conversion efficiencies that reach 26% that of N719.

**Scheme 7:**
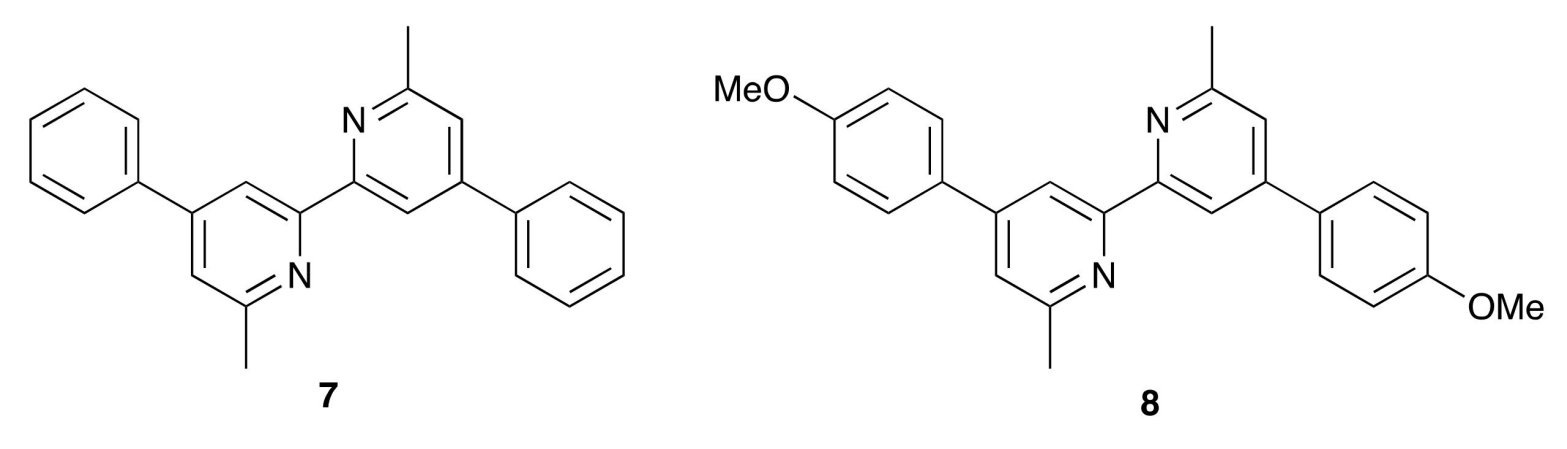
Structures of the previously reported ancillary ligands **7** and **8** [41].

The introduction of the electron-donating MeO and Me_2_N groups was expected to be beneficial in terms of the “push–pull” dye design. An overview of bisdiimine copper(I) dyes draws attention to the most efficient ancillary ligands being those with electron-releasing substituents [12]. We have previously described the performances of fully-masked DSCs sensitized with [Cu(**6**)(**7**)]^+^ and [Cu(**6**)(**8**)]^+^ in which ancillary ligands **7** and **8** are 6,6′-dimethyl-4,4′-diphenyl-2,2-bipyridine and 4,4′-bis(4-methoxyphenyl)-6,6′-dimethyl-2,2-bipyridine (Scheme 7). On going from [Cu(**6**)(**7**)]^+^ to [Cu(**6**)(**8**)]^+^, an increase in *J*_SC_ (4.27 to 4.87 mA cm^−2^ for the best-performing cells) and *η* (1.66 to 1.82%) was observed [41]. However, for the Schiff base ancillary ligands, changing the functionality from phenyl to 4-methoxyphenyl on-going from [Cu(**6**)(**1**)]^+^ to [Cu(**6**)(**4**)]^+^ results in lower *J*_SC_ values (2.74–2.96 mA cm^−2^ for [Cu(**6**)(**4**)]^+^ compared to 3.64–4.08 mA cm^−2^ for [Cu(**6**)(**1**)]^+^; Table 1). The lower value of EQE_max_ (34%, Figure 10) is consistent with the drop in *J*_SC_. A small decrease in *V*_OC_ is also observed, leading to lower values of solar cell efficiencies (*η* = 0.99–1.08%). A more substantial decrease in performance is observed when the 4-dimethylamino groups are introduced. On-going from [Cu(**6**)(**1**)]^+^ to [Cu(**6**)(**5**)]^+^, values of *J*_SC_ decrease significantly to 1.49–1.56 mA cm^−2^ and there is an associated decrease in the maximum EQE to ca. 17% (Figure 10). There is also a detrimental influence on *V*_OC_ (Figure 9 and Table 2), leading to poor global efficiencies of ca. 0.45%.

Since the performance measurements for DSCs sensitized with [Cu(**6**)(**1**)]^+^, [Cu(**6**)(**4**)]^+^, [Cu(**6**)(**7**)]^+^, and [Cu(**6**)(**8**)]^+^ were made under the same conditions (electrodes materials, electrolyte, instrumentation, fully masked), a comparison of the data sheds light on the role of the imine unit in the ancillary ligands. Table 3 compares the data. The pair of dyes [Cu(**6**)(**1**)]^+^ and [Cu(**6**)(**7**)]^+^ differ only in the presence or absence of the imine spacer, respectively, as does the pair [Cu(**6**)(**4**)]^+^ and [Cu(**6**)(**8**)]^+^ (see Scheme 2 and Scheme 7). In both cases, introducing the imine unit results in a decrease in *J*_SC_ and *V*_OC_, and a loss in overall performance. Thus, not only does the imine spacer hinder the beneficial effects of the electron-releasing methoxy group, it is also disadvantageous in the case of the peripheral phenyl substituent.

## 3. Materials and Methods 

### 3.1. General 

^1^H, ^13^C{^1^H}, and ^31^P{^1^H} NMR spectra were recorded on a Bruker Avance III-500 spectrometer (Bruker BioSpin AG, Fällanden, Switzerland) at 298 K. The ^1^H and ^13^C NMR chemical shifts were referenced with respect to residual solvent peaks (*δ* TMS = 0) and ^31^P NMR chemical shifts with respect to *δ* (85% aqueous H_3_PO_4_) = 0 ppm. A Shimadzu LCMS-2020 instrument (Shimadzu Schweiz GmbH, 4153 Reinach, Switzerland) was used to record electrospray ionization (ESI) mass spectra with samples introduced as MeOH solutions. High-resolution ESI (HR-ESI) mass spectra were measured on a Bruker maXis 4G QTOF instrument (Bruker BioSpin AG, Fällanden, Switzerland). FT-infrared (IR) and solution absorption spectra were measured using PerkinElmer UATR Two (Perkin Elmer, 8603 Schwerzenbach, Switzerland) and UV-2600 (Shimadzu Schweiz GmbH, 4153 Reinach, Switzerland) spectrophotometers, respectively, and solid-state absorption spectra were recorded using a Cary-5000 (Agilent Technologies Inc., Santa Clara, CA, United States) instrument. 

Electrochemical measurements used a CH Instruments 900B potentiostat (CH Instruments, Texas 78738, USA) with [^n^Bu_4_N][PF_6_] (0.1 M) as supporting electrolyte and a scan rate of 0.1 V s^−1^; the solvent was CH_2_Cl_2_ with sample concentrations ca. 2 × 10^−3^ mol dm^−3^. The working electrode was platinum (for [Cu(**5**)_2_][PF_6_]) or glassy carbon. A leakless Ag^+^/AgCl (eDAQ ET069-1) (eDAQ Europe, 38640 Claix, France) reference electrode was used, and the counter-electrode was a platinum wire. Final potentials were externally referenced with respect to the Fc/Fc^+^ couple.

All reactions were carried out with chemicals used as received from Sigma Aldrich (Sigma Aldrich Chemie GmbH, 89555 Steinheim, Germany) or Fluorochem (Chemie Brunschwig AG, 4052 Basel, Switzerland) without further purification. [Cu(MeCN)_4_][PF_6_] [42] and compound **6** [34] were prepared according to the literature.

### 3.2. 4,4′,6,6-Tetramethyl-2,2′-Bipyridine

The method of preparation was based on that of Mukkala and Kankare [43] with a longer reaction time of 5 days. ^1^H NMR spectroscopic data were consistent with those reported [43].

### 3.3. Synthesis of (1E,1′E)-2,2′-(6,6′-Dimethyl[2,2′-bipyridine]-4,4′-diyl)bis(N,N-dimethylethen-1-amine)

The method was based on that described in the literature [32] using 10 equivalents of Bredereck′s reagent to one equivalent of 4,4′,6,6′-tetramethyl-2,2′-bipyridine. The reaction was monitored by ^1^H NMR spectroscopy until it went to completion. The ^1^H NMR spectrum of the product was consistent with the literature [32].

### 3.4. Synthesis of 6,6′-Dimethyl-[2,2′-Bipyridine]-4,4′-Dicarbaldehyde

The compound was prepared as reported by Dupau et al. [32], but with a 9-fold excess of NaIO_4_. The spectroscopic data were in agreement with those reported [32,44]. 

### 3.5. General Procedure for the Synthesis of the Schiff Base Ligands

Compounds **1**–**5** were prepared by condensation of 6,6′-dimethyl-[2,2′-bipyridine]-4,4′-dicarbaldehyde with the appropriate amine according to the following procedure [45]. Anhydrous MgSO_4_ (0.4 g) and 1.0 equivalent of 6,6′-dimethyl-[2,2′-bipyridine]-4,4′-dicarbaldehyde were added to a solution of the amine (1.0 mmol, 2.0 eq.) in CH_2_Cl_2_ (5 mL) at ambient temperature (ca. 22 °C). The mixture was stirred overnight at room temperature, and was then filtered. The residue was washed with CH_2_Cl_2_ and the volume of the filtrate reduced under reduced pressure. The product was precipitated from CH_2_Cl_2_/hexane, was collected by filtration, and was washed with hexane and diethyl ether. Reaction scales and yields are given below.

### 3.6. Compound ***1***

The method was as above using 6,6′-dimethyl-[2,2′-bipyridine]-4,4′-dicarbaldehyde (100 mg, 0.416 mmol, 1.0 eq.), aniline (0.077 mL, 0.82 mmol, 2.0 eq.), MgSO_4_ anhydrous (400 mg, 1.66 mmol, 8.0 eq.) and dry CH_2_Cl_2_ (10 mL). Compound **1** was isolated as a beige powder in (103 mg, 0.264 mmol, 63.4%). M.p = 216 °C. ^1^H NMR (500 MHz, CD_2_Cl_2_) *δ*/ppm: 8.68 (s, 2H, H^A3^), 8.58 (s, 2H, H^a^), 7.72 (s, 2H, H^A5^), 7.45 (td, *J* = 7.8, 1.8 Hz, 4H, H^B3^), 7.35–7.26 (m, 6H, H^B2+B4^), 2.73 (s, 6H, H^Me^). ^13^C{^1^H} NMR (126 MHz, CD_2_Cl_2_) *δ*/ppm: 159.6 (C^A6^), 159.4 (C^a^), 156.8 (C^A2^), 151.9 (C^B1^), 144.8 (C^A4^), 129.8 (C^B3^), 127.3 (C^B4^), 122.0 (C^A5^), 121.5 (C^B2^), 117.9 (C^A3^), 24.9 (C^Me^). HRMS *m/z* 391.1913 [M+H]^+^ (calc. 391.1917). UV–vis (CH_2_Cl_2_, 2 × 10^−5^ mol dm^−3^): λ_max_ /nm 256 (ε/dm^3^ mol^−1^ cm^−1^ 44,800), 327 (30,450). IR spectrum: see Figure S1.

### 3.7. Compound ***2***

The method was as in Section 3.5, using 6,6′-dimethyl-[2,2′-bipyridine]-4,4′-dicarbaldehyde (100 mg, 0.416 mmol, 1.0 eq.), 4-methylaniline (91.9 mg, 0.832 mmol, 2.0 eq.), MgSO_4_ anhydrous (400 mg, 1.66 mmol, 8.0 eq.), and dry CD_2_Cl_2_ (10 mL). Compound **2** was isolated as a beige powder (122 mg 0.291 mmol, 70.1%). M.p. = 217 °C. ^1^H NMR (500 MHz, CD_2_Cl_2_) *δ*/ppm: 8.66 (d, *J* = 1.4 Hz, 2H, H^A3^), 8.59 (s, 2H, H^a^), 7.71 (d, *J* = 1.4 Hz, 2H, H^A5^), 7.26 (m, 4H, H^B3^), 7.22 (m, 4H, H^B2^), 2.72 (s, 6H, H^Me(A6)^), 2.31 (s, 6H, H^Me(B4)^). ^13^C{^1^H} NMR (126 MHz, CD_2_Cl_2_) *δ*/ppm: 159.6 (C^A6^), 158.4 (C^a^), 156.8 (C^A2^), 149.1 (C^B1^), 144.9 (C^A4^), 137.6 (C^B4^), 130.5 (C^B2^), 121.5 (C^A5^), 121.9 (C^B3^), 117.8 (C^A3^), 24.9 (C^Me(A6)^), 21.3 (C^Me(B4)^). HRMS *m/z* 419.2222 [M+H]^+^ (calc. 419.2230). UV–vis (CH_2_Cl_2_, 2 x 10^−5^ mol dm^−3^): λ_max_/nm 333 (ε/dm^3^ mol^−1^ cm^−1^ 20,100). IR spectrum: see Figure S2.

### 3.8. Compound ***3***

The method was as in Section 3.5, using 6,6′-dimethyl-[2,2′-bipyridine]-4,4′-dicarbaldehyde (100 mg, 0.416 mmol, 1.0 eq.), 4-*tert*-butylaniline (124 mg, 0.832 mmol, 2.0 eq.), MgSO_4_ anhydrous (400 mg, 1.66 mmol, 8.0 eq.), and dry CH_2_Cl_2_ (10 mL). Compound **3** was isolated as a beige powder (115 mg, 0.229 mmol, 55.0%). M.p. = 291 °C. ^1^H NMR (500 MHz, CD_2_Cl_2_) *δ*/ppm: 8.66 (s, 2H, H^A3^), 8.61 (s, 2H, H^a^), 7.72 (s, 2H, H^A5^), 7.48 (d, *J* = 8.3 Hz, 4H, H^B3^), 7.26 (d, *J* = 8.3 Hz, 4H, H^B2^), 2.73 (s, 6H, H^Me^), 1.36 (s, 18H, H*^t^*^Bu^). ^13^C{^1^H} NMR (126 MHz, CD_2_Cl_2_) *δ*/ppm: 160.0 (^A6^), 158.9 (^a^), 157.3 (^A2^), 151.2 (^B4^), 149.5 (^B1^), 145.4 (C^A4^), 127.2 (C^B3^), 122.3 (C^A5^), 121.7 (C^B2^), 118.3 (C^A3^), 35.5 (C^C(*t*Bu)^), 32.1 (C^Me(*t*Bu)^), 25.3 (C^Me(A6)^). HRMS *m/z* 503.3175 [M+H]^+^ (calc. 503.3169). UV–vis (CH_2_Cl_2_, 2 × 10^−5^ mol dm^−3^): λ/nm 331 (ε/dm^3^ mol^−1^ cm^−1^ 20,000). IR spectrum: see Figure S3.

### 3.9. Compound ***4***

The method was as in Section 3.5, using 6,6′-dimethyl-[2,2′-bipyridine]-4,4′-dicarbaldehyde (100 mg, 0.416 mmol, 1.0 eq.), 4-methoxyaniline (102 mg, 0.832 mmol, 2.0 eq.), MgSO_4_ anhydrous (400 mg, 1.66 mmol, 8.0 eq.), and dry CH_2_Cl_2_ (10 mL). Compound **4** was isolated as a beige powder (127 mg, 0.282 mmol, 67.8%). M.p. = 215 °C. ^1^H NMR (500 MHz, CD_2_Cl_2_) *δ*/ppm: 8.65 (s, 2H, H^A3^), 8.61 (s, 2H, H^a^), 7.70 (s, 2H, H^A5^), 7.34 (m, 4H, H^B2^), 6.98 (m, 4H, H^B3^), 3.85 (s, 6H, H^OMe^), 2.72 (s, 6H, H^Me(A6)^). ^13^C{^1^H} NMR (126 MHz, CD_2_Cl_2_) *δ*/ppm: 159.5 (C^B4^), 159.3 (C^A6^), 156.6 (C^a^), 145.6 (C^A4^), 144.2 (C^B1^), 123.0 (C^B2^), 121.5 (C^A5^), 117.6 (C^A3^), 114.9 (C^B3^), 55.9 (C^OMe^), 24.8 (C^Me(A6)^). HRMS *m/z* 451.2121 [M+H]^+^ (calc. 451.2129). UV–vis (CH_2_Cl_2_, 2 × 10^−5^ mol dm^−3^): λ/nm 283 sh (ε/dm^3^ mol^−1^ cm^−1^ 21,000), 344 (28,900). IR spectrum: see Figure S4.

### 3.10. Compound ***5***

The method was as in Section 3.5, using 6,6′-dimethyl-[2,2′-bipyridine]-4,4′-dicarbaldehyde (100 mg, 0.416 mmol, 1.0 eq.), 4-(dimethylamino)aniline (117 mg, 0.832 mmol, 2.0 eq.), MgSO_4_ anhydrous (400 mg, 1.66 mmol, 8.0 eq.), and dry CH_2_Cl_2_ (10 mL). Compound **5** was isolated as a yellow powder (118 mg, 0.248 mmol, 59.5%). M.p. = 284 °C. ^1^H NMR (500 MHz, CD_2_Cl_2_) *δ*/ppm: 8.63 (s, 2H, H^a^), 8.61 (s, 2H, H^A3^), 7.68 (s, 2H, H^A5^), 7.36 (m, 4H, H^B2^), 6.78 (m, 4H, H^B3^), 3.01 (s, 12H, H^NMe^), 2.71 (s, 6H, H^Me(A6)^). ^13^C{^1^H} NMR (126 MHz, CD_2_Cl_2_) *δ*/ppm: 159.4 (C^A6^), 153.6 (C^a^), 150.9 (C^B4^), 145.6 (C^A4^), 139.9 (C^B1^), 123.3 (C^B2^), 121.3 (C^A5^), 117.5 (C^A3^), 113.1 (C^B3^), 40.9 (C^NMe^), 24.9 (C^Me(A6)^). HRMS *m/z* 477.2757 [M+H]^+^ (calc. 477.2761). UV–vis (CH_2_Cl_2_, 2 × 10^−5^ mol dm^−3^): λ/nm 250 (ε/dm^3^ mol^−1^ cm^−1^ 48,500), 316 (22,200), 420 (43,200). IR spectrum: see Figure S5.

### 3.11. General Procedure for the Synthesis of Copper Complexes

[Cu(MeCN)_4_][PF_6_] (1 eq.) and the diimine ligand (2 eq.) were dissolved in CH_2_Cl_2_ in a round-bottomed flask. The reaction mixture immediately turned red and was stirred for 20 min. After evaporation of the solvent, the product was recrystallized from CH_2_Cl_2_/diethyl ether (*v/v* 1:10) and the resulting microcrystalline solid was collected by filtration, washed with diethyl ether, and dried under vacuum. Scales of reactions are given below.

### 3.12. [Cu(**1**)_2_][PF_6_]

The reaction was carried out as described above using compound **1** (40.0 mg, 0.102 mmol, 2.0 eq.), [Cu(MeCN)_4_][PF_6_] (19.1 mg, 0.0512 mmol, 1.0 eq.) and dry CH_2_Cl_2_ (10 mL). [Cu(**1**)_2_][PF_6_] was isolated as a red solid (44.0 mg, 0.0445 mmol, 86.9%). ^1^H NMR (500 MHz, CD_2_Cl_2_) *δ*/ppm 8.87 (s, 4H, H^A3^), 8.72 (s, 4H, H^a^), 7.98 (s, 4H, H^A5^), 7.50 (m, 8H, H^B3^), 7.40–7.35 (overlapping m, 12H, H^B2+B4^), 2.38 (s, 12H, H^Me^).^13^C{^1^H} NMR (126 MHz, CD_2_Cl_2_) *δ*/ppm 158.4 (C^A6^), 156.9 (C^a^), 152.6 (C^A2^), 150.8 (C^B1^), 145.6 (C^A4^), 129.9 (C^B3^), 128.1 (C^B4^), 125.5 (C^A5^), 121.5 (C^B2^), 118.8 (C^A3^), 25.5 (C^Me^). ^31^P{^1^H} NMR (202 MHz, CD_2_Cl_2_) *δ*/ppm: −144.4 (hept, *J* = 7101 Hz). ESI MS *m*/*z* 843.25 [M–PF_6_]^+^ (base peak, calc. 843.30). HR-ESI MS *m*/*z* 843.2968 [M–PF_6_]^+^ (calc. 843.2979). UV–vis (CH_2_Cl_2_, 2 × 10^−6^ M): λ/nm 266 nm (ε/dm^3^ mol^−1^ cm^−1^ 49,950), 335 (50,400), 518 (18,450). Found C 60.88, H 4.35, N 10.87; required for C_52_H_44_CuF_6_N_8_P.2H_2_O C 60.90, H 4.72, N 10.93. IR spectrum: see Figure S17.

### 3.13. [Cu(**2**)_2_][PF_6_]

The reaction was carried out as described in Section 3.11, using compound **2** (30.0 mg, 0.0716 mmol, 2.0 eq.), [Cu(MeCN)_4_]PF_6_] (13.3 mg, 0.0358 mmol, 1.0 eq.), and dry CH_2_Cl_2_ (10 mL). [Cu(**2**)_2_][PF_6_] was isolated as a red solid (31.9 mg, 0.0305 mmol, 85.2%). ^1^H NMR (500 MHz, CD_2_Cl_2_) *δ*/ppm: 8.85 (s, 4H, H^A3^), 8.73 (s, 4H, H^a^), 7.96 (s, 4H, H^A5^), 7.34–7.28 (overlapping m, 16H, H^B2+B3^), 2.41 (s, 12H, H^Me(B4)^), 2.36 (s, 12H, H^Me(A6)^). ^13^C{^1^H} NMR (126 MHz, CD_2_Cl_2_) *δ*/ppm: 158.5 (C^A6^), 156.0 (C^a^), 152.8 (C^A2^), 148.3 (C^B1^), 146.0 (C^A4^), 138.8 (C^B4^), 130.7 (C^B3^), 125.5 (C^A5^), 121.8 (^B2^), 118.9 (C^A3^), 25.6, (C^Me(A6)^), 21.4 (C^Me(B4)^). ^31^P{^1^H} NMR (202 MHz, CD_2_Cl_2_) *δ*/ppm = −144.4 (hept, *J* = 710 Hz). ESI MS *m*/*z* 899.28 [M–PF_6_]^+^ (base peak, calc. 899.36). HR-ESI MS *m*/*z* 899.3594 [M–PF_6_]^+^ (calc. 899.3605). UV–vis (CH_2_Cl_2_, 2 × 10^−6^ M): λ/nm 282 nm (ε/dm^3^ mol^−1^ cm^−1^ 50,400), 343 (56,900), 513 (20,650). Found C 62.80, H 5.21, N 10.68; required for C_56_H_52_CuF_6_N_8_P.1.5H_2_O C 62.71, H 5.17, N 10.45. IR spectrum: see Figure S18.

### 3.14. [Cu(**3**)_2_][PF_6_]

The reaction was carried out as described in Section 3.11, using compound **3** (30.0 mg, 0.0596 mmol, 2.0 eq.), [Cu(MeCN)_4_]PF_6]_ (11.1 mg, 0.0298 mmol, 1.0 eq.), and dry CH_2_Cl_2_ (10 mL). [Cu(**3**)_2_][PF_6_] was isolated as a red solid (25.4 mg, 0.0209 mmol, 70.2%). ^1^H NMR (500 MHz, CD_2_Cl_2_) *δ*/ppm: 8.86 (s, 4H, H^A3^), 8.74 (s, 4H, ^a^), 7.97 (s, 4H, H^A5^), 7.52 (m, 8H, H^B3^), 7.36 (m, 8H, H^B2^), 2.36 (s, 12H, H^Me^), 1.37 (s, 36H, H*^t^*^Bu^). ^13^C{^1^H} NMR (126 MHz, CD_2_Cl_2_) *δ*/ppm: 158.5 (C^A6^), 156.4 (C^a^), 152.9 (C^A2^), 152.0 (C^B4^), 148.2 (C^B1^), 146.0 (C^A4^), 127.0 (C^B3^), 125.5 (C^A5^), 121.5 (C^B2^), 118.9 (C^A3^), 35.2 (C^C(*t*Bu)^), 31.64 (C^Me(*t*Bu)^), 25.6 (C^Me(A6)^). ^31^P{^1^H} NMR (202 MHz, CD_2_Cl_2_) *δ*/pp: −144.4 (hept, *J* = 711 Hz). ESI MS *m*/*z* 1067.40 [M–PF_6_]^+^ (calc. 1067.55), 1099.47 [M–PF_6_+MeOH]^+^ (base peak, calc. 1099.58), 1131.50 [M–PF_6_+2MeOH]^+^ (calc. 1131.60). HR-ESI MS *m*/*z* 1067.5464 [M–PF_6_]^+^ (calc. 1067.5483). UV–vis (CH_2_Cl_2_, 2 × 10^−6^ M): λ/nm 281 nm (ε/dm^3^ mol^−1^ cm^−1^ 48,100), 340 (53,850), 516 (20,100). Found C 65.88, H 6.47, N 9.69; required for C_68_H_76_CuF_6_N_8_P.1.5H_2_O C 65.82, H 6.42, N 9.03. IR spectrum: see Figure S19.

### 3.15. [Cu(**4**)_2_][PF_6_]

The reaction was carried out as described in Section 3.11, using compound **4** (30.0 mg, 0.0666 mmol, 2.0 eq.), [Cu(MeCN)_4_]PF_6_] (12.4 mg, 0.0333 mmol, 1.0 eq.) and dry CH_2_Cl_2_ (10 mL). [Cu(**4**)_2_][PF_6_] was isolated as a red solid (27.0 mg, 0.0243 mmol, 73.1%). ^1^H NMR (500 MHz, CD_2_Cl_2_) *δ*/ppm: 8.83 (s, 4H, H^A3^), 8.72 (s, 4H, H^a^), 7.92 (s, 4H, H^A5^), 7.44 (m, 8H, H^B2^), 7.02 (m, 8H, H^B3^), 3.87 (s, 12H, H^OMe^), 2.35 (s, 12H, H^Me(A6)^). ^13^C{^1^H} NMR (126 MHz, CD_2_Cl_2_) *δ*/ppm: 160.3 (C^B4^), 158.2 (C^A6^), 153.9 (C^a^), 152.2 (C^A2^), 145.6 (C^A4^), 144.2 (C^B1^), 123.0 (C^B2^), 125.2 (C^A5^), 118.6 (C^A3^), 115.0 (C^B3^), 56.0 (C^OMe^), 25.4 (C^Me(A6)^). ^31^P{^1^H} NMR (202 MHz, CD_2_Cl_2_) *δ*/ppm: −144.4 (hept, *J* = 711 Hz). ESI MS *m*/*z* 963.31 [M–PF_6_]^+^ (base peak, calc. 963.34). HR-ESI MS *m*/*z* 963.3399 [M–PF_6_]^+^ (calc. 963.3402). UV–vis (CH_2_Cl_2_, 2 × 10^−6^ M): λ/nm 289 nm (ε/dm^3^ mol^−1^ cm^−1^ 38,400), 345 sh (42,100), 370 (49,250), 518 (19,900). Found C 59.40, H 4.61, N 9.81; required for C_56_H_52_CuF_6_N_8_O_4_P.H_2_O C 59.65, H 4.83, N 9.94. IR spectrum: see Figure S20.

### 3.16. [Cu(**5**)_2_][PF_6_]

The reaction was carried out as described in Section 3.11, using compound **5** (60.0 mg, 0.126 mmol, 2.0 eq.), [Cu(MeCN)_4_]PF_6_ (23.4 mg, 0.0629 mmol, 1.0 eq.), and dry CH_2_Cl_2_ (10 mL). [Cu(**5**)_2_][PF_6_] was isolated as a red solid (56.2 mg, 0.0484 mmol, 76.9%). ^1^H NMR (500 MHz, CD_2_Cl_2_) *δ*/ppm: 8.78 (s, 4H, H^A3^), 8.72 (s, 4H, H^a^), 7.87 (s, 4H, H^A5^), 7.46 (m, 8H, H^B2^), 6.80 (m, 8H, H^B3^), 3.05 (s, 24H, ^NMe^), 2.33 (s, 12H, H^Me(A6)^). ^13^C{^1^H} NMR (126 MHz, CD_2_Cl_2_) *δ*/ppm: 157.9 (C^A6^), 152.6 (C^A2^), 151.3 (C^B4^), 149.9 (C^a^), 146.3 (C^A4^), 138.6 (C^B1^), 124.5 (C^A5^), 123.7 (C^B2^), 118.0 (C^A3^), 112.7 (C^B3^), 40.5 (C^NMe^), 25.4 (C^Me(A6)^). ^31^P{^1^H} NMR (202 MHz, CD_2_Cl_2_) *δ*/ppm −144.4 (hept, *J* = 710 Hz). ESI MS *m*/*z* 1015.40 [M–PF_6_]^+^ (base peak, calc. 1015.4667). HR-ESI MS *m*/*z* 1015.4658 [M–PF_6_]^+^ (calc. 1015.47). UV–vis (CH_2_Cl_2_, 2 (CH_2_Cl_2_, 2 × 10^−6^ M): λ/nm 260 nm (ε/dm^3^ mol^−1^ cm^−1^ 73,900), 325 (43,300), 461 (69,850), 530 sh (54,600). Found C 60.22, H 5.84, N 13.82; required for C_60_H_64_CuF_6_N_12_P.2H_2_O C 60.17, H 5.72, N 14.03. IR spectrum: see Figure S21.

### 3.17. Crystallography

Single crystal data were collected on a Bruker APEX-II diffractometer (CuKα radiation) with data reduction, solution and refinement using the programs APEX [46], ShelXT [47], Olex2 [48], and ShelXL v. 2014/7 [49]. SQUEEZE [50] was used for the solvent regions and the electrons removed equated to one molecule of Et_2_O per compound formula unit. The analysis of the structure was made using Mercury CSD v. 4.3.0 [51,52]. 

C_56_CuF_6_H_54_N_8_OP, *M_r_* = 1063.58, dark red block, tetragonal, space group *P*4_1_22, *a* = 11.1913(7), *b* = 11.1913(7), *c* = 39.724(3) Å, *V* = 4975.2(7) Å^3^, *D*_c_ = 1.420 g cm^−3^, *T* = 130 K, *Z* = 4, *Z′* = 0.5, μ(CuK_a_) = 1.535 mm^−1^. Total 25745 reflections measured, 4642 unique (*R_int_* = 0.0318). Refinement of 4412 reflections (310 parameters) with *I* > 2*σ*(*I*) converged at final *R*_1_ = 0.0679 (*R*_1_ all data = 0.0705), *wR*_2_ = 0.2001 (*wR*_2_ all data = 0.2035), gof = 1.031. CCDC 1982171.

### 3.18. DSC Fabrication

FTO/TiO_2_ electrodes (Solaronix Test Cell Titania Electrodes, Solaronix SA, Aubonne, Switzerland) were washed with milliQ water and EtOH, heated at 450 °C for 30 min, then cooled to ca. 60 °C. The electrodes were immediately placed in a DMSO solution of **6** (1.0 mM) for 24 h, after which they were removed, washed with DMSO and CH_2_Cl_2_, then dried under N_2_. Each electrode functionalized with **6** was immersed in a CH_2_Cl_2_ solution (0.1 mM) of [CuL_2_][PF_6_] (L = **1**, **2**, **3**, **4** or **5**) for 3 days at ambient temperature (ca. 22 °C). After removing the electrodes from the dye-baths, they were washed with CH_2_Cl_2_ and dried under a flow of N_2_. For the reference dye N719 (Solaronix SA, Aubonne, Switzerland), FTO/TiO_2_ electrodes (Solaronix Test Cell Titania Electrodes, Solaronix SA, Aubonne, Switzerland) were immersed in a solution of N719 (EtOH, 0.3 mM) for ca. 24 h, removed from the solution, washed with EtOH and dried under a flow of N_2_. Counter electrodes (Solaronix Test Cell Platinum Electrodes, Solaronix SA, Aubonne, Switzerland) were washed with EtOH and heated at 450 °C for 30 min to remove volatile organics.

The working and counter-electrode for each DSC were combined using thermoplast hot-melt sealing foil (Solaronix Test Cell Gaskets, 60 µm, Solaronix SA, Aubonne, Switzerland) and the gap between the electrodes was filled with electrolyte (LiI (0.1 M), I_2_ (0.05 M), 1-methylbenzimidazole (0.5 M), 1-butyl-3-methylimidazolinium iodide (0.6 M) in 3-methoxypropionitrile) by vacuum backfilling through a hole in the counter-electrode. This hole was finally sealed (Solaronix Test Cell Sealings and Solaronix Test Cell Caps, Solaronix SA, Aubonne, Switzerland). 

### 3.19. Electrodes for Solid-State Absorption Spectroscopy.

The fabrication method described above for the FTO/TiO_2_ electrodes was followed, but using Solaronix Test Cell Titania Electrodes Transparent (Solaronix SA, Aubonne, Switzerland).

### 3.20. DSC and EQE Measurements 

The DSCs were masked using black-coloured copper sheet with an accurately calibrated aperture smaller than the TiO_2_ surface area. Black tape was used to complete the masking at the top and sides of the cells. Performance measurements were made by irradiating the DSCs from behind with a LOT Quantum Design LS0811 instrument (LOT-QuantumDesign GmbH, Darmstadt, Germany, 100 mW cm^−2^ = 1 sun, AM1.5 G conditions). The simulated light power was calibrated using an Si reference cell. 

EQE measurements were made using a Spe Quest quantum efficiency setup (Rera Systems, Nijmegen, The Netherlands) with a 100 W halogen lamp (QTH) and a lambda 300 grating monochromator (LOT-Oriel GmbH & Co. KG, Darmstadt, Germany). The monochromatic light was modulated to 3 Hz using a chopper wheel (ThorLabs Inc., Newton, NJ, USA), and the cell response was amplified with a large dynamic range IV converter (Melles Griot B.V., Didam, Netherlands) and measured with a SR830 DSP Lock-In amplifier (Stanford Research Systems Inc., Sunnyvale, CA, USA). 

## 4. Conclusions

We have prepared and characterized a series of new Schiff base ligands **1**–**5** bearing bpy metal-binding domains and *N*-arylmethaniminyl substituents. Their homoleptic copper(I) complexes [CuL_2_][PF_6_] were also synthesized. The single crystal structure of [Cu(**1**)_2_][PF_6_]·Et_2_O confirmed a distorted tetrahedral coordination environment; in the Schiff base ligand, the C=N bonds lie approximately in the same plane of the pyridine ring to which each is connected, while the phenyl rings are twisted out of this plane. The solution absorption spectrum of each of [Cu(**1**)_2_][PF_6_], [Cu(**2**)_2_][PF_6_], and [Cu(**3**)_2_][PF_6_] shows an MLCT absorption (518, 513, and 516 nm, respectively), while for [Cu(**4**)_2_][PF_6_] and [Cu(**5**)_2_][PF_6_], ILCT bands are observed; overlap of the ILCT and MLCT in [Cu(**5**)_2_][PF_6_] results in a substantial increase in MLCT intensity and a red-shift. 

Heteroleptic [Cu(**6**)(L_ancillary_)]^+^ dyes with L_ancillary_ = **1**–**5** were assembled on FTO-TiO_2_ electrodes and incorporated into DSCs. The best-performing sensitizer is [Cu(**6**)(**1**)]^+^, with a values of *η* up to 1.51% compared to 5.74% for a DSC sensitized by N719. The introduction of the electron-donating MeO (in **4**) and Me_2_N (in **5**) groups results in a decrease in *J*_SC_ and EQE_max_, and for [Cu(**6**)(**5**]^+^, low values of *V*_OC_ are also observed. Comparisons between performances of DSCs containing [Cu(**6**)(**1**)]^+^ and [Cu(**6**)(**4**)]^+^ with those sensitized by related dyes [Cu(**6**)(**7**)]^+^ and [Cu(**6**)(**8**)]^+^ in which the outer phenyl rings are directly bonded to the bpy domain [41] reveal that not only does the imine spacer hinder the favourable effects of the electron-donating MeO substituent, it is also detrimental in the case of the peripheral phenyl substituent. 

## Figures and Tables

**Table 1:** Cyclic voltammetric data for [CuL_2_][PF_6_] compounds referenced to external Fc/Fc^+^ = 0.0 V; CH_2_Cl_2_ solutions (ca. 2 × 10^−3^ mol dm^−3^) with [*^n^*Bu_4_N][PF_6_] as supporting electrolyte and scan rate of 0.1 V s^−1^.

Compound	*E*_1/2_^ox^/V	*E*_pc_ – *E*_pa_/mV	*E*_pc_^a^	*E*_pa_^a,c^/V
**2**				−2.11
**5**			+0.39	−2.17
[Cu(**1**)_2_][PF_6_]	+0.49^b^	130		−1.68, –1.89, –2.04
[Cu(**2**)_2_][PF_6_]	+0.49^b^	70		−1.69, −1.96, −2.04
[Cu(**3**)_2_][PF_6_]	+0.48^b^	70		−1.71, −1.92, −2.07, −2.21
[Cu(**4**)_2_][PF_6_]	+0.49^b^	90		−1.74, −1.95, −2.12
[Cu(**5**)_2_][PF_6_]			+0.38,^c^ +0.54^b^	−1.78, −2.01, −2.11, −2.20

^a^ Values for *E*_pc_ or *E*_pa_ are given because the processes are partially reversible or irreversible; ^b^ copper-centered; ^c^ ligand-centered.

**Table 2:** Performance parameters (under 1 sun illumination) of triplicate, fully-masked DSCs on the day of sealing the cells with the dyes [Cu(**6**)(**1**)]^+^, [Cu(**6**)(**2**)]^+^, [Cu(**6**)(**3**)]^+^, [Cu(**6**)(**4**)]^+^, and [Cu(**6**)(**5**)]^+^. The data are compared to a DSC containing the reference dye N719. Relative *η* values are with respect to N719 set at 100%.

Dye	Cell Number	*J*_SC_/mA cm^−2^	*V*_OC_/mV	*ff*/%	*η*/%	Relative *η*/%
[Cu(**6**)(**1**)]^+^	1	4.08	538	69	1.51	26
[Cu(**6**)(**1**)]^+^	2	3.64	520	69	1.32	23
[Cu(**6**)(**1**)]^+^	3	3.87	534	70	1.44	25
[Cu(**6**)(**2**)]^+^	1	3.40	526	71	1.28	22
[Cu(**6**)(**2**)]^+^	2	3.01	530	72	1.14	20
[Cu(**6**)(**2**)]^+^	3	3.44	524	70	1.26	22
[Cu(**6**)(**3**)]^+^	1	4.08	522	68	1.45	25
[Cu(**6**)(**3**)]^+^	2	3.51	522	70	1.28	22
[Cu(**6**)(**3**)]^+^	3	3.40	518	71	1.26	22
[Cu(**6**)(**4**)]^+^	1	2.83	508	70	1.01	18
[Cu(**6**)(**4**)]^+^	2	2.96	516	71	1.08	19
[Cu(**6**)(**4**)]^+^	3	2.74	514	70	0.99	17
[Cu(**6**)(**5**)]^+^	1	1.56	455	65	0.46	8
[Cu(**6**)(**5**)]^+^	2	1.49	461	66	0.45	8
[Cu(**6**)(**5**)]^+^	3	1.55	455	65	0.46	8
N719		13.42	640	67	5.74	100

**Table 3:** Performance parameters (under 1 sun illumination) of the best-performing, fully-masked DSCs on the day of sealing the cells with the dyes [Cu(**6**)(**1**)]^+^, [Cu(**6**)(**4**)]^+^, [Cu(**6**)(**7**)]^+^, and [Cu(**6**)(**8**)]^+^.

Dye	Imine Spacer in L_ancillary_	*J*_SC_/mA cm^−2^	*V*_OC_/mV	*ff*/%	*η*/%	Reference
[Cu(**6**)(**1**)]^+^	yes	4.08	538	69	1.51	This work
[Cu(**6**)(**4**)]^+^	yes	2.96	516	71	1.08	This work
N719		13.42	640	67	5.74	This work
[Cu(**6**)(**7**)]^+^	no	4.27	545	71	1.66	[41]
N719		13.29	647	67	5.79	[41]
[Cu(**6**)(**8**)]^+^	no	4.87	528	71	1.82	[41]
N719		13.91	635	68	6.04	[41]

## Supplementary Materials

Supplementary materials can be found at https://www.mdpi.com/1422-0067/21/5/1735/s1. Figures S1–S5: IR spectra of ligands; Figures S6–S16: NMR spectra of ligands; Figures S17–S21: IR spectra of complexes; Figures S22–S26: mass spectra of complexes; Figures S27–S36: HMQC and HMBC spectra of complexes; Figures S37–S41: *J–V* curves for triplicate DSCs; Figures S42–S46: EQE spectra for triplicate DSCs.
