# Supplementary material for: Schiff Base Ancillary Ligands in Bis(diimine) Copper(I) Dye-Sensitized Solar Cells
Source: Int J Mol Sci. 2020 Mar 3;21(5):1735. doi: 10.3390/ijms21051735 (PMC7084427; doi:10.3390/ijms21051735)

checkCIF/PLATON report

Structure factors have been supplied for datablock(s) pfphcomplex_130k_0m

THIS REPORT IS FOR GUIDANCE ONLY. IF USED AS PART OF A REVIEW PROCEDURE FOR PUBLICATION, IT SHOULD NOT REPLACE THE EXPERTISE OF AN EXPERIENCED CRYSTALLOGRAPHIC REFEREE.

No syntax errors found. CIF dictionary Interpreting this report

Datablock: pfphcomplex_130k_0m

Bond precision: C-C = 0.0114 A Wavelength=1.54178

Cell:	a=11.1913(7)	b=11.1913(7)	c=39.724(3)
	alpha=90	beta=90	gamma=90
Temperature:	130 K		

	Calculated	Reported
Volume	4975.2(7)	4975.2(7)
Space group	P 41 2 2	P 41 2 2
Hall group	P 4w 2c	P 4w 2c
Moiety formula	C52 H44 Cu N8, F6 P [+ solvent]	C52 H44 Cu N8, F6 P, 1[C4H10O1]
Sum formula	C52 H44 Cu F6 N8 P [+ solvent]	C56 H54 Cu F6 N8 O P
Mr	989.47	1063.58
Dx,g cm-3	1.321	1.420
Z	4	4
Mu (mm-1)	1.481	1.535
F000	2040.0	2208.0
F000’	2039.42	
h,k,lmax	13,13,48	13,13,46
Nref	4757[2842]	4642
Tmin,Tmax	0.772,0.807	0.613,0.753
Tmin’	0.701	

Correction method= # Reported T Limits: Tmin=0.613 Tmax=0.753
AbsCorr = MULTI-SCAN

Data completeness= 1.63/0.98 Theta(max)= 70.215

R(reflections)= 0.0679(4412) wR2(reflections)= 0.2035(4642)

S = 1.031 Npar= 310

The following ALERTS were generated. Each ALERT has the format
test-name_ALERT_alert-type_alert-level.
Click on the hyperlinks for more details of the test.

Alert level B

```
PLAT230_ALERT_2_B Hirshfeld Test Diff for    N4       --C21      .        8.5 s.u.
PLAT230_ALERT_2_B Hirshfeld Test Diff for    C8       --C9       .       14.6 s.u.
PLAT369_ALERT_2_B Long   C(sp2)-C(sp2) Bond  C8       - C12      .       1.58 Ang.
PLAT987_ALERT_1_B The Flack x is >> 0 -  Do a BASF/TWIN Refinement     Please Check
```

Alert level C

```
PLAT234_ALERT_4_C Large Hirshfeld Difference C21      --C26      .       0.16 Ang.
PLAT241_ALERT_2_C High   MainMol Ueq as Compared to Neighbors of         C9 Check
PLAT241_ALERT_2_C High   MainMol Ueq as Compared to Neighbors of        C18 Check
PLAT260_ALERT_2_C Large Average Ueq of Residue Including        P1      0.164 Check
PLAT341_ALERT_3_C Low Bond Precision on  C-C Bonds ...............     0.0114 Ang.
PLAT410_ALERT_2_C Short Intra H...H Contact  H12      ..H22      .       1.98 Ang.
                                                     x,y,z  =      1_555 Check
PLAT411_ALERT_2_C Short Inter H...H Contact  H18      ..H18      .       2.14 Ang.
                                              x,-1-y,1/2-z  =      6_545 Check
PLAT911_ALERT_3_C Missing FCF Refl Between Thmin & STh/L=    0.600          4 Report
PLAT918_ALERT_3_C Reflection(s) with I(obs) much Smaller I(calc) .          1 Check
```

Alert level G

```
FORMU01_ALERT_2_G  There is a discrepancy between the atom counts in the
            _chemical_formula_sum and the formula from the _atom_site* data.
            Atom count from _chemical_formula_sum:C56 H54 Cu1 F6 N8 O1 P1
            Atom count from the _atom_site data:  C52 H44 Cu1 F6 N8 P1
CELLZ01_ALERT_1_G Difference between formula and atom_site contents detected.
CELLZ01_ALERT_1_G ALERT: Large difference may be due to a
            symmetry error - see SYMMG tests
           From the CIF: _cell_formula_units_Z    4
           From the CIF: _chemical_formula_sum  C56 H54 Cu F6 N8 O P
           TEST: Compare cell contents of formula and atom_site data

           atom    Z*formula  cif sites diff
           C        224.00    208.00   16.00
           H        216.00    176.00   40.00
           Cu         4.00      4.00    0.00
           F         24.00     24.00    0.00
           N         32.00     32.00    0.00
           O          4.00      0.00    4.00
           P          4.00      4.00    0.00
PLAT012_ALERT_1_G N.O.K.   _shelx_res_checksum Found in CIF ......     Please Check
PLAT033_ALERT_4_G Flack x Value Deviates > 3.0 * sigma from Zero .      0.057 Note
PLAT041_ALERT_1_G Calc. and Reported SumFormula    Strings  Differ     Please Check
PLAT051_ALERT_1_G Mu(calc) and Mu(CIF) Ratio Differs from 1.0 by .       3.49 %
PLAT068_ALERT_1_G Reported F000 Differs from Calcd (or Missing)...     Please Check
PLAT072_ALERT_2_G SHELXL First  Parameter in WGHT  Unusually Large       0.12 Report
PLAT244_ALERT_4_G Low    Solvent Ueq as Compared to Neighbors of         P1 Check
PLAT605_ALERT_4_G Largest Solvent Accessible VOID in the Structure        125 A**3
PLAT868_ALERT_4_G ALERTS Due to the Use of _smtbx_masks Suppressed          ! Info
PLAT912_ALERT_4_G Missing # of FCF Reflections Above STh/L=  0.600         38 Note
PLAT952_ALERT_5_G Calculated (ThMax) and CIF-Reported Lmax Differ           2 Units
PLAT958_ALERT_1_G Calculated (ThMax) and Actual (FCF) Lmax Differ           2 Units
PLAT978_ALERT_2_G Number C-C Bonds with Positive Residual Density.          2 Info
PLAT992_ALERT_5_G Repd & Actual _reflns_number_gt Values Differ by          1 Check
```

```
  0 ALERT level A = Most likely a serious problem - resolve or explain
  4 ALERT level B = A potentially serious problem, consider carefully
  9 ALERT level C = Check. Ensure it is not caused by an omission or oversight
 17 ALERT level G = General information/check it is not something unexpected

  8 ALERT type 1 CIF construction/syntax error, inconsistent or missing data
 11 ALERT type 2 Indicator that the structure model may be wrong or deficient
  3 ALERT type 3 Indicator that the structure quality may be low
  6 ALERT type 4 Improvement, methodology, query or suggestion
  2 ALERT type 5 Informative message, check
```

Validation response form

Please find below a validation response form (VRF) that can be filled in and pasted into your CIF.

```
# start Validation Reply Form
_vrf_PLAT234_pfphcomplex_130k_0m
;
PROBLEM: Large Hirshfeld Difference C21      --C26      .       0.16 Ang.
RESPONSE: ...
;
_vrf_PLAT241_pfphcomplex_130k_0m
;
PROBLEM: High   MainMol Ueq as Compared to Neighbors of         C9 Check
RESPONSE: ...
;
_vrf_PLAT260_pfphcomplex_130k_0m
;
PROBLEM: Large Average Ueq of Residue Including        P1      0.164 Check
RESPONSE: ...
;
_vrf_PLAT341_pfphcomplex_130k_0m
;
PROBLEM: Low Bond Precision on  C-C Bonds ...............     0.0114 Ang.
RESPONSE: ...
;
_vrf_PLAT410_pfphcomplex_130k_0m
;
PROBLEM: Short Intra H...H Contact  H12      ..H22      .       1.98 Ang.
RESPONSE: ...
;
_vrf_PLAT411_pfphcomplex_130k_0m
;
PROBLEM: Short Inter H...H Contact  H18      ..H18      .       2.14 Ang.
RESPONSE: ...
;
_vrf_PLAT911_pfphcomplex_130k_0m
;
PROBLEM: Missing FCF Refl Between Thmin & STh/L=    0.600          4 Report
RESPONSE: ...
;
_vrf_PLAT918_pfphcomplex_130k_0m
;
PROBLEM: Reflection(s) with I(obs) much Smaller I(calc) .          1 Check
RESPONSE: ...
;
# end Validation Reply Form
```

It is advisable to attempt to resolve as many as possible of the alerts in all categories. Often the minor alerts point to easily fixed oversights, errors and omissions in your CIF or refinement strategy, so attention to these fine details can be worthwhile. In order to resolve some of the more serious problems it may be necessary to carry out additional measurements or structure refinements. However, the purpose of your study may justify the reported deviations and the more serious of these should normally be commented upon in the discussion or experimental section of a paper or in the "special_details" fields of the CIF. checkCIF was carefully designed to identify outliers and unusual parameters, but every test has its limitations and alerts that are not important in a particular case may appear. Conversely, the absence of alerts does not guarantee there are no aspects of the results needing attention. It is up to the individual to critically assess their own results and, if necessary, seek expert advice.

Publication of your CIF in IUCr journals

A basic structural check has been run on your CIF. These basic checks will be run on all CIFs submitted for publication in IUCr journals (*Acta Crystallographica*, *Journal of Applied Crystallography*, *Journal of Synchrotron Radiation*); however, if you intend to submit to *Acta Crystallographica Section C* or *E* or *IUCrData*, you should make sure that full publication checks are run on the final version of your CIF prior to submission.

Publication of your CIF in other journals

Please refer to the *Notes for Authors* of the relevant journal for any special instructions relating to CIF submission.

PLATON version of 22/12/2019; check.def file version of 13/12/2019

Datablock pfphcomplex_130k_0m - ellipsoid plot

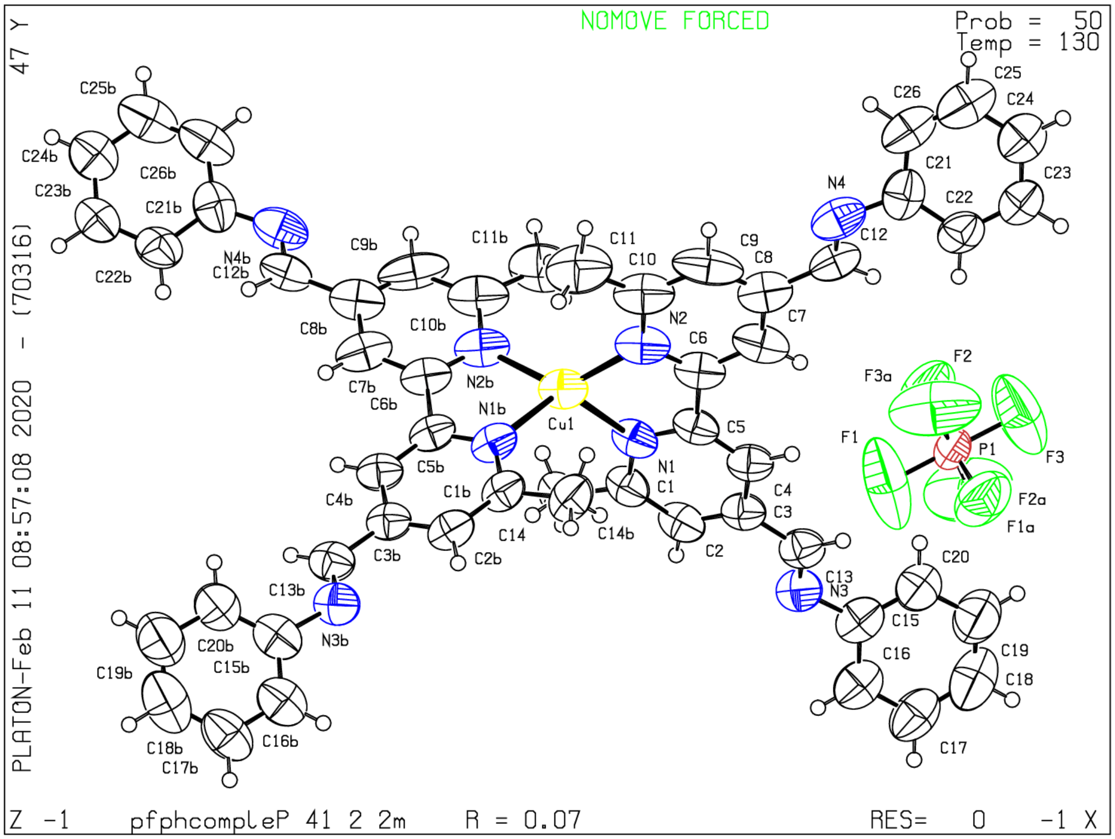

Supplement: Supplementary file 1 [file ijms-21-01735-s001.zip › checkcif.pdf]
