# Supplementary material for: Schiff Base Ancillary Ligands in Bis(diimine) Copper(I) Dye-Sensitized Solar Cells
Source: Int J Mol Sci. 2020 Mar 3;21(5):1735. doi: 10.3390/ijms21051735 (PMC7084427; doi:10.3390/ijms21051735)
Supplement: Supplementary file 1 [file ijms-21-01735-s001.zip › Supporting information 21Feb 2020.docx]

Supporting information

Schiff Base Ancillary Ligands in Bis(diimine) Copper(I) Dye-Sensitized Solar Cells

Elias Lüthi,^1^ Paola Andrea Forero Cortés,^1^ Alessandro Prescimone,^1^ Edwin C. Constable^1^ and Catherine E. Housecroft^1^*

^1^Department of Chemistry, University of Basel, BPR 1096, Mattenstrasse 24a, CH-4058 Basel, Switzerland


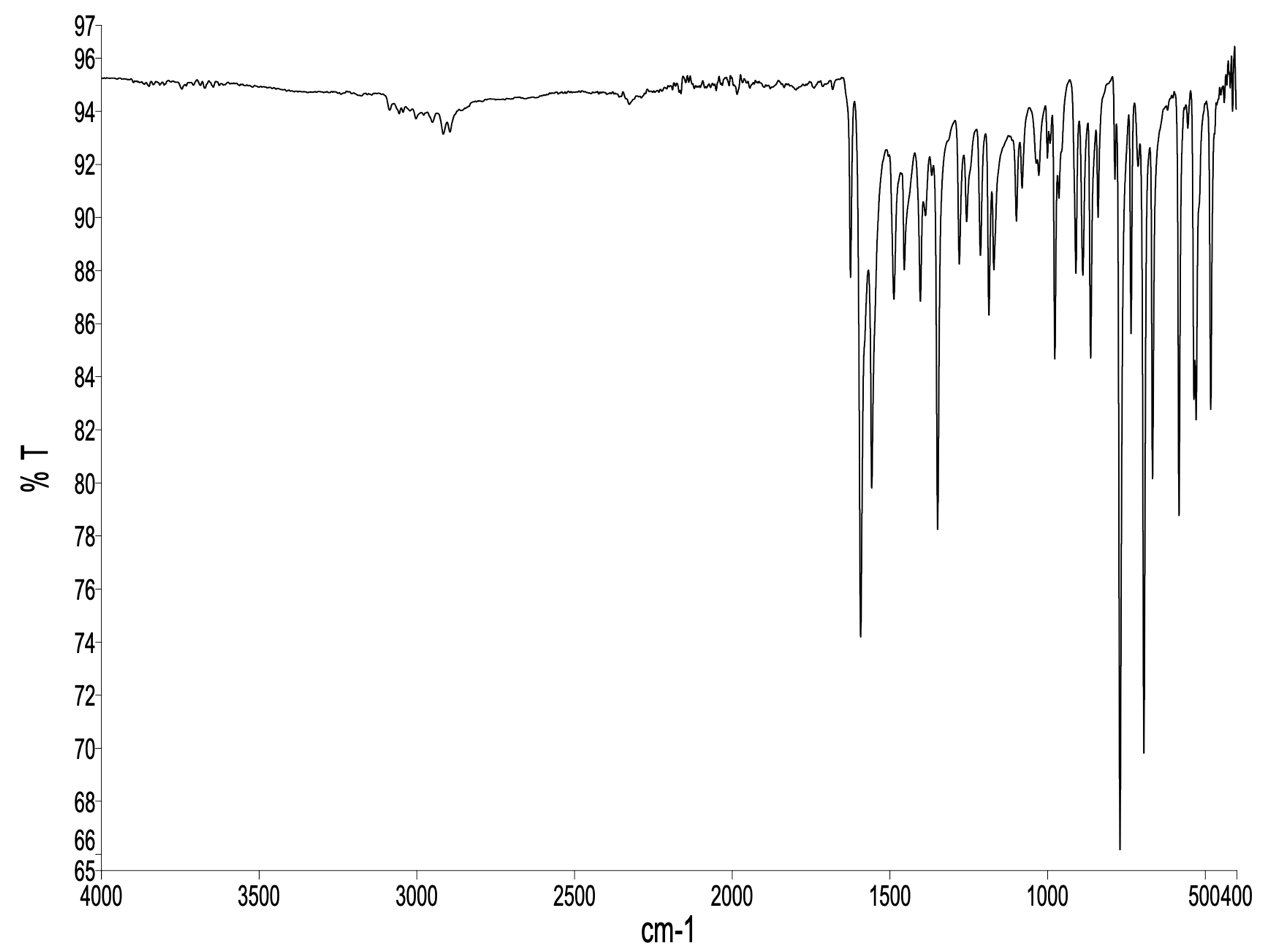


Figure S1. Solid-state FT-IR spectrum of ligand **1**.


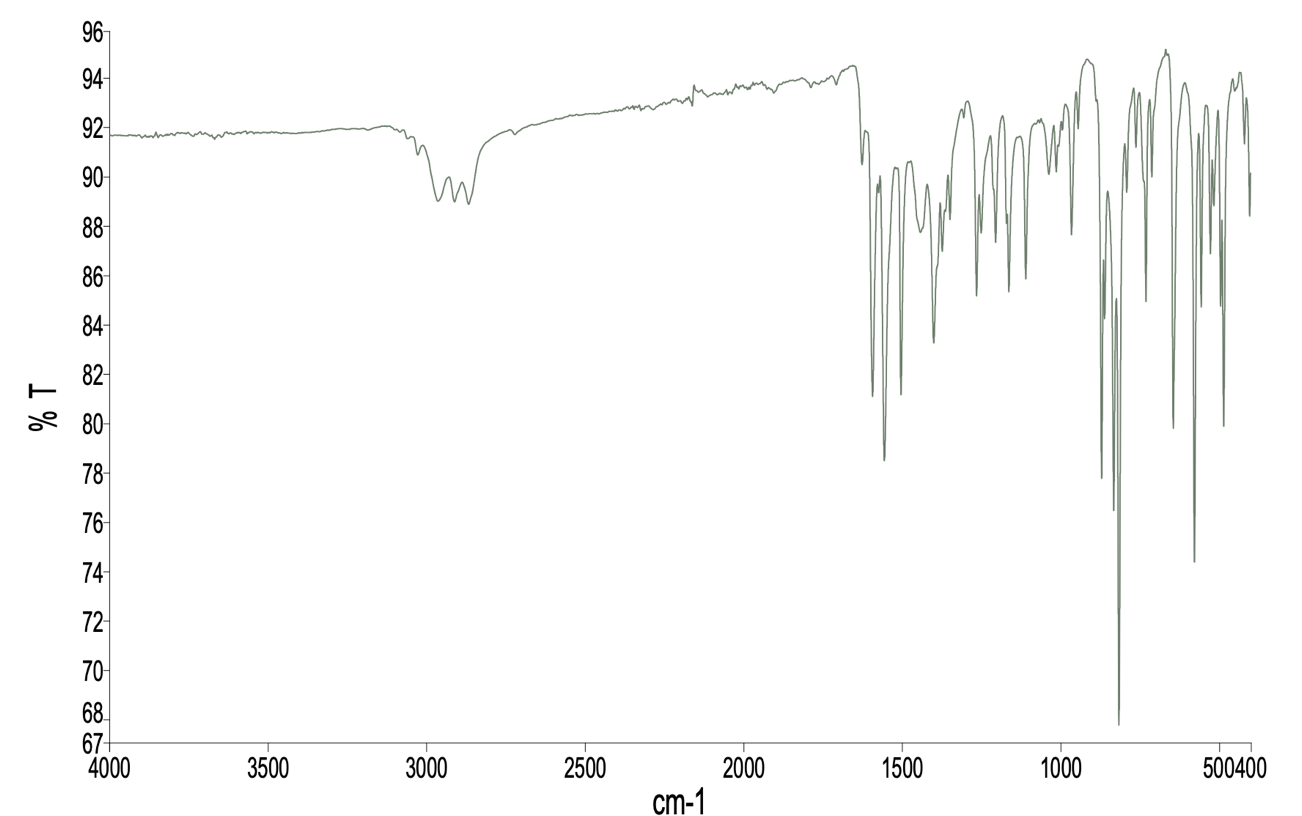


Figure S2. Solid-state FT-IR spectrum of ligand **2**.


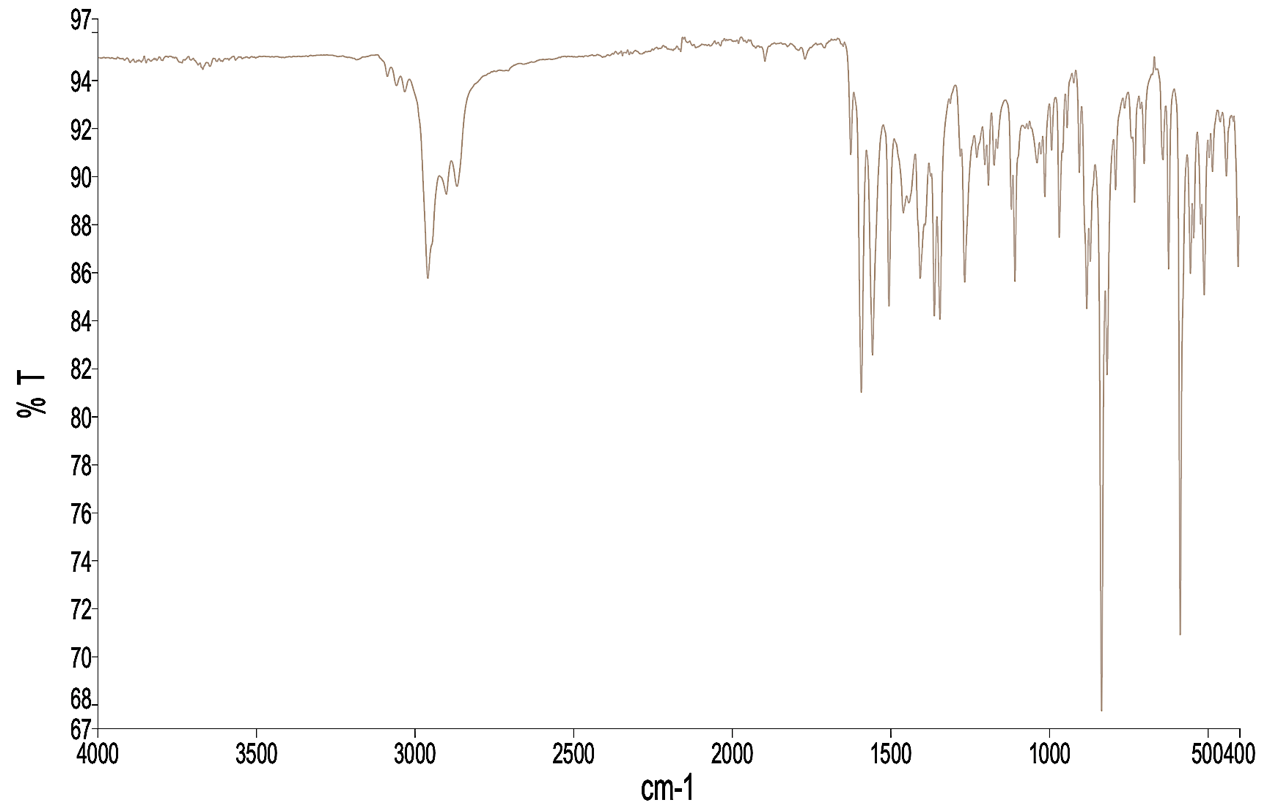


Figure S3. Solid-state FT-IR spectrum of ligand **3**.


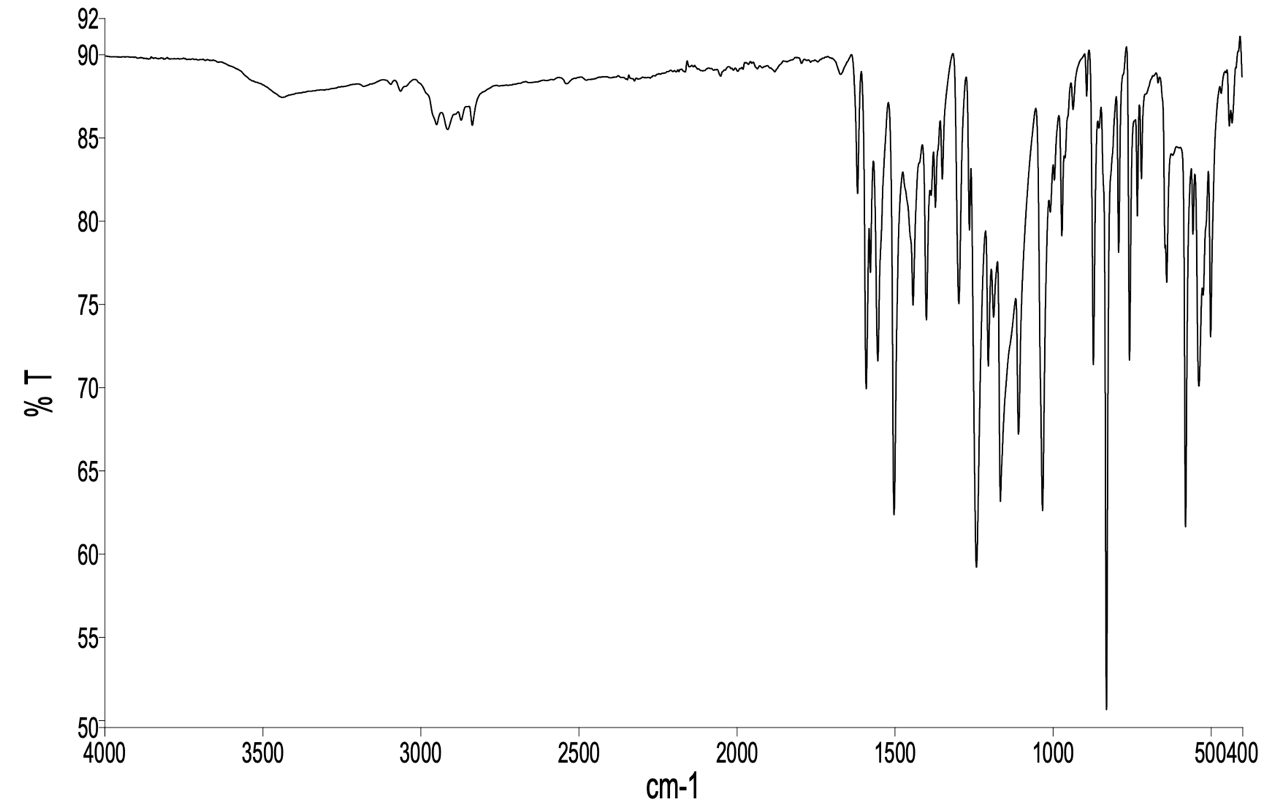


Figure S4. Solid-state FT-IR spectrum of ligand **4**.


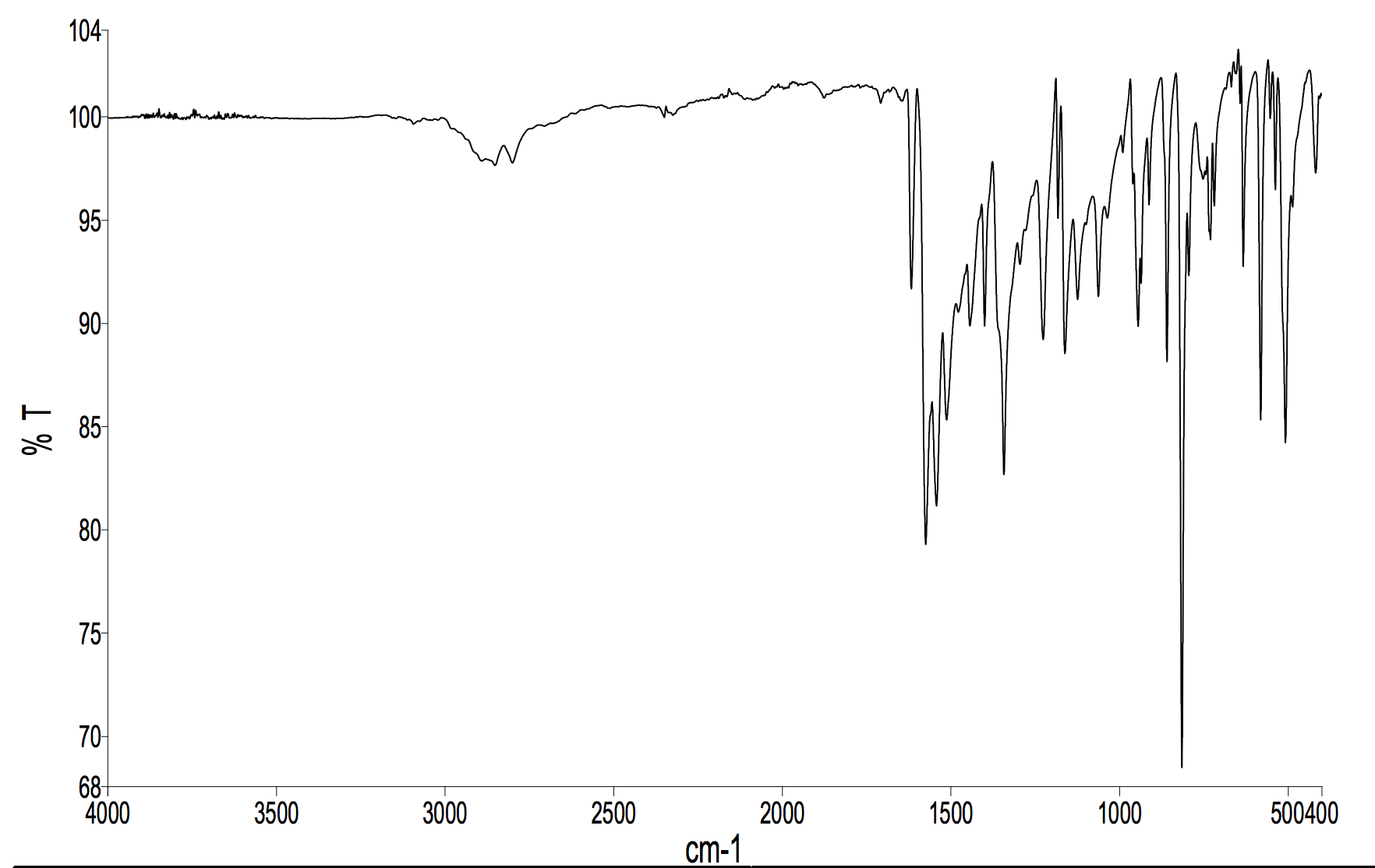


Figure S5. Solid-state FT-IR spectrum of ligand **5**.


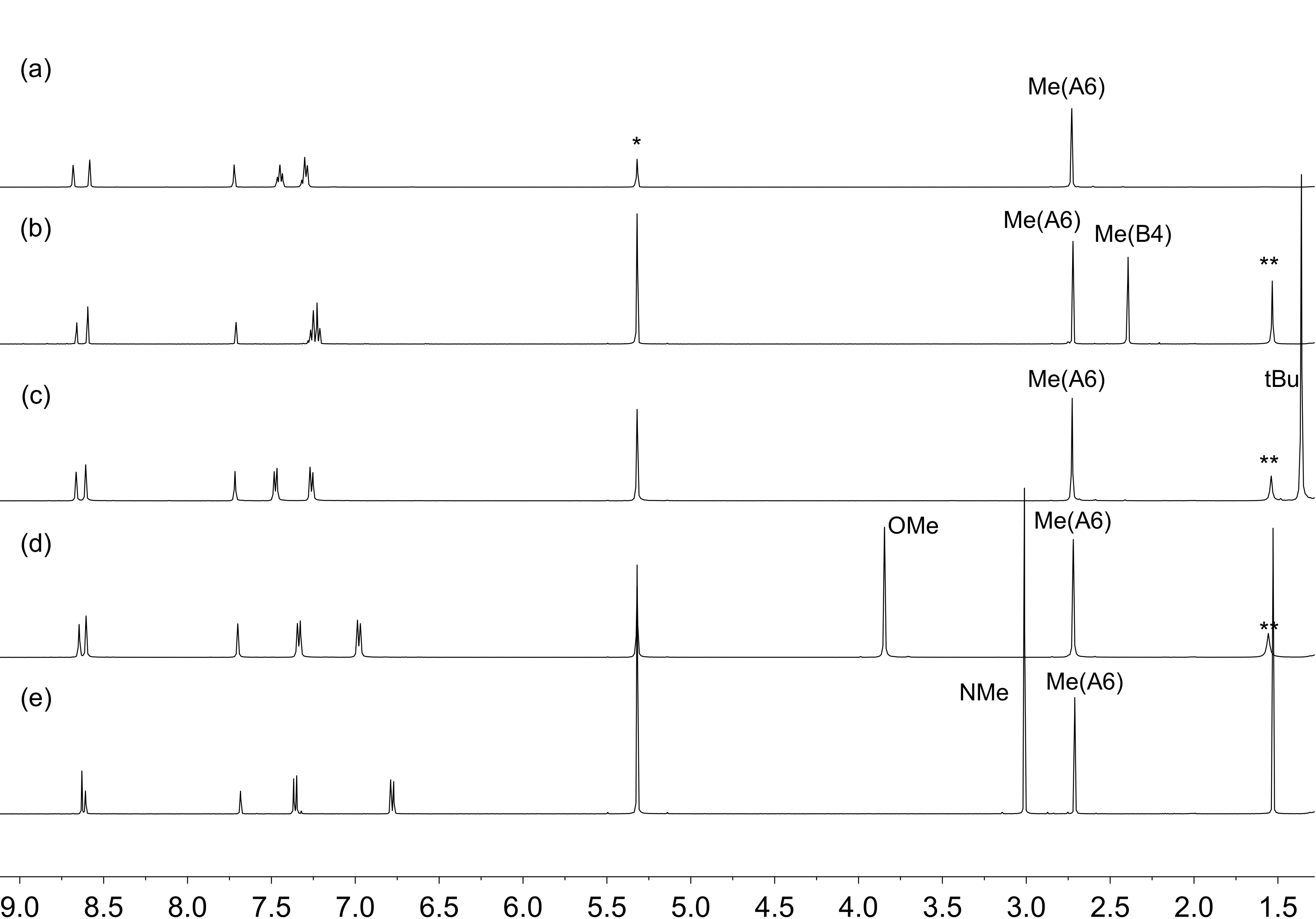


Figure S6. ^1^H NMR (500 MHz, CD_2_Cl_2_, 298 K) spectra of ligands (a) **1**, (b) **2**, (c) **3**, (d) **4**, and (e) **5**. See Scheme 1 for atom labels. * = residual CHDCl_2_; ** = H_2_O.


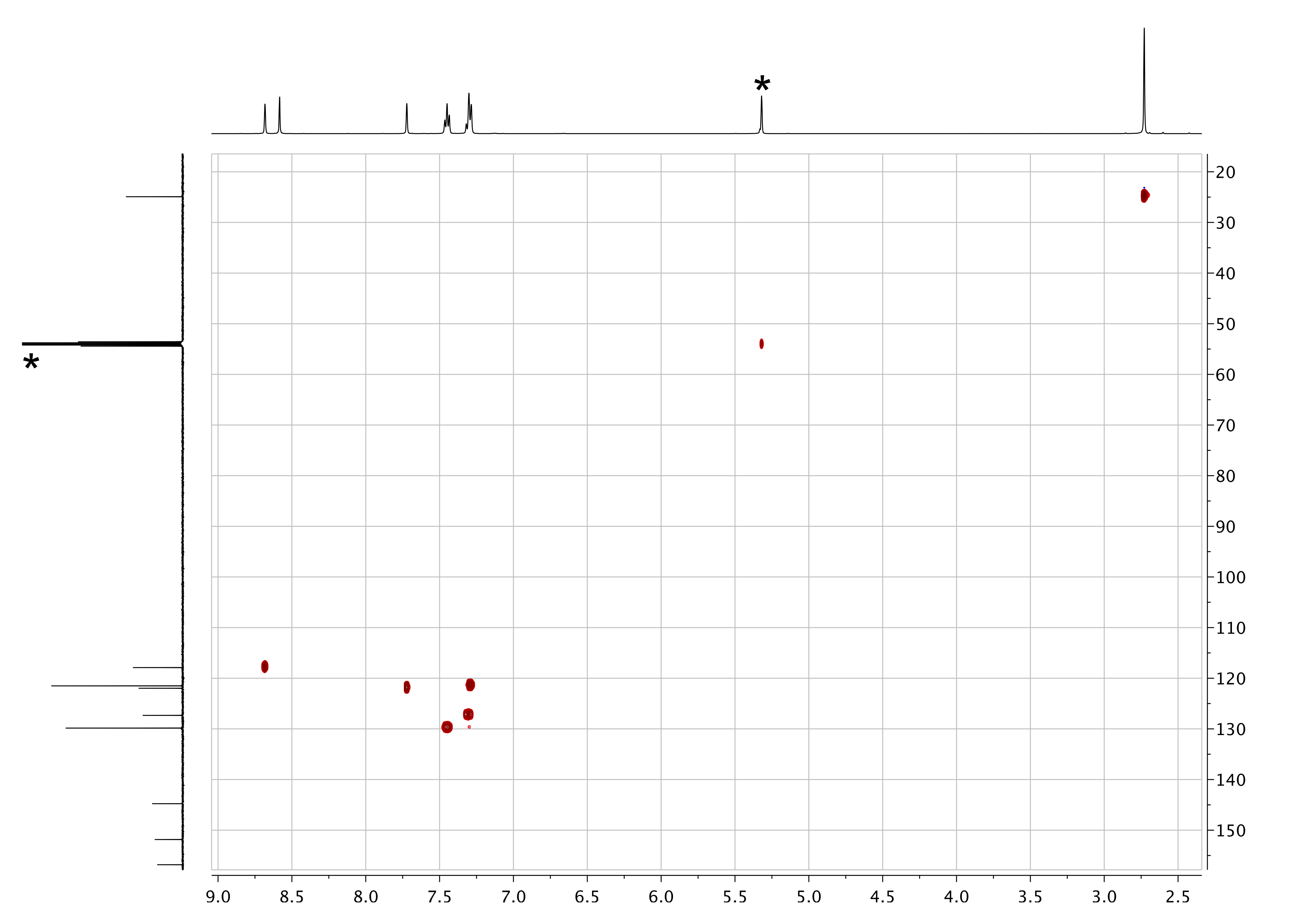


Figure S7. HMQC of ligand **1** (500 MHz ^1^H, 126 MHz ^13^C, CD_2_Cl_2_, 298 K). * = residual CHDCl_2_.


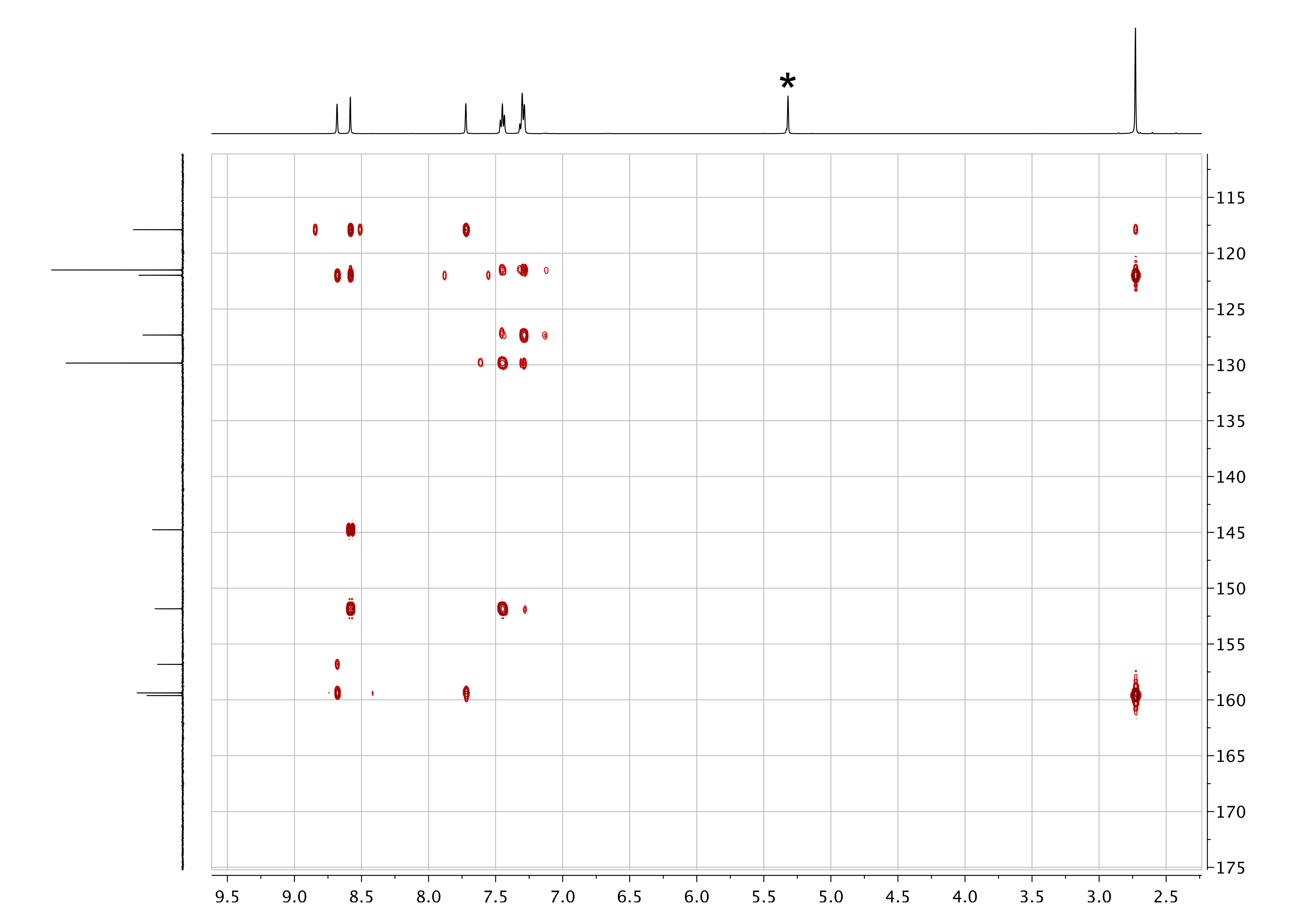


Figure S8. HMBC of of ligand **1** (500 MHz ^1^H, 126 MHz ^13^C, CD_2_Cl_2_, 298 K). * = residual CHDCl_2_.

**
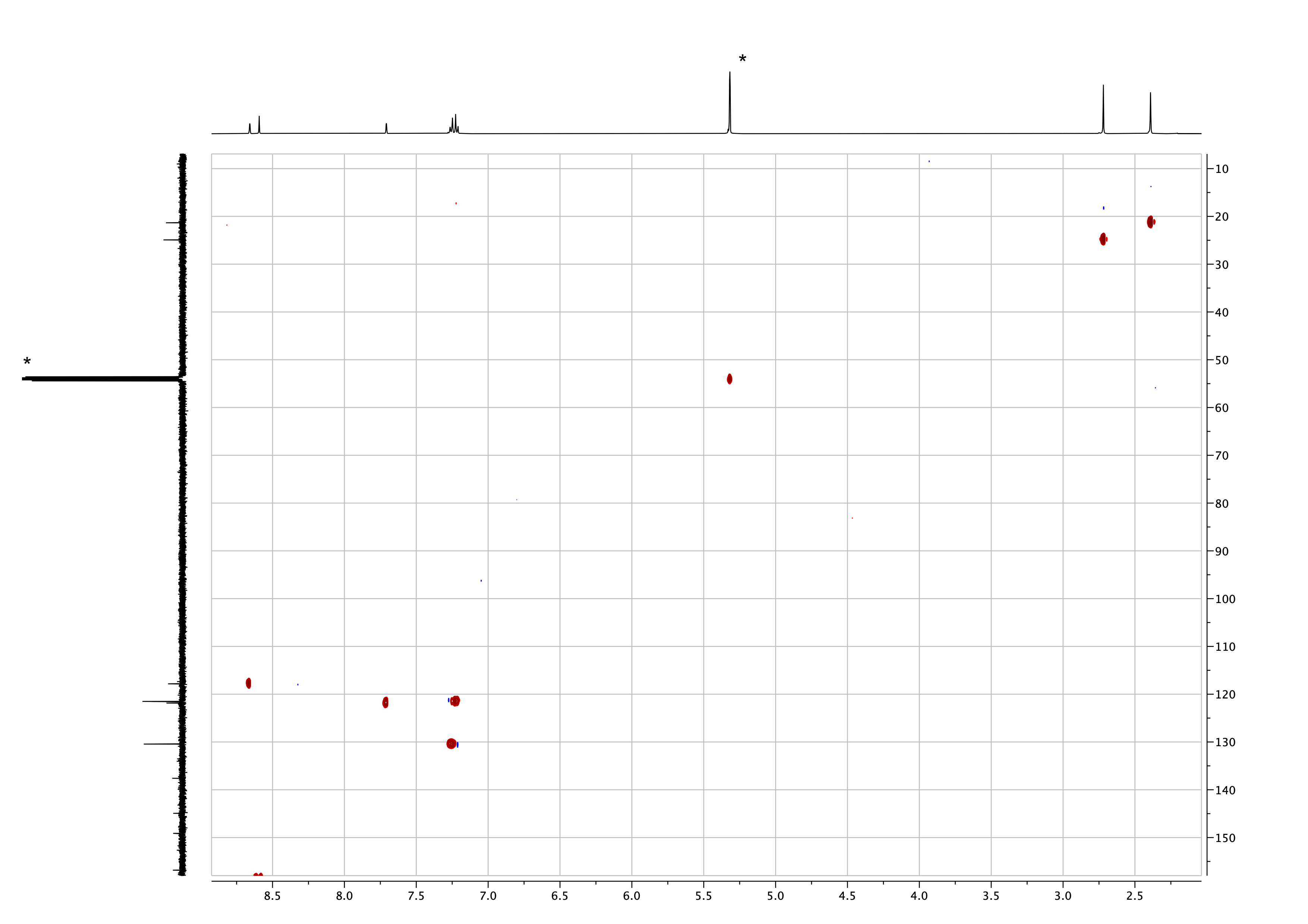
**

Figure S9. HMQC of ligand **2** (500 MHz ^1^H, 126 MHz ^13^C, CD_2_Cl_2_, 298 K). * = residual CHDCl_2_ or CD_2_Cl_2_.


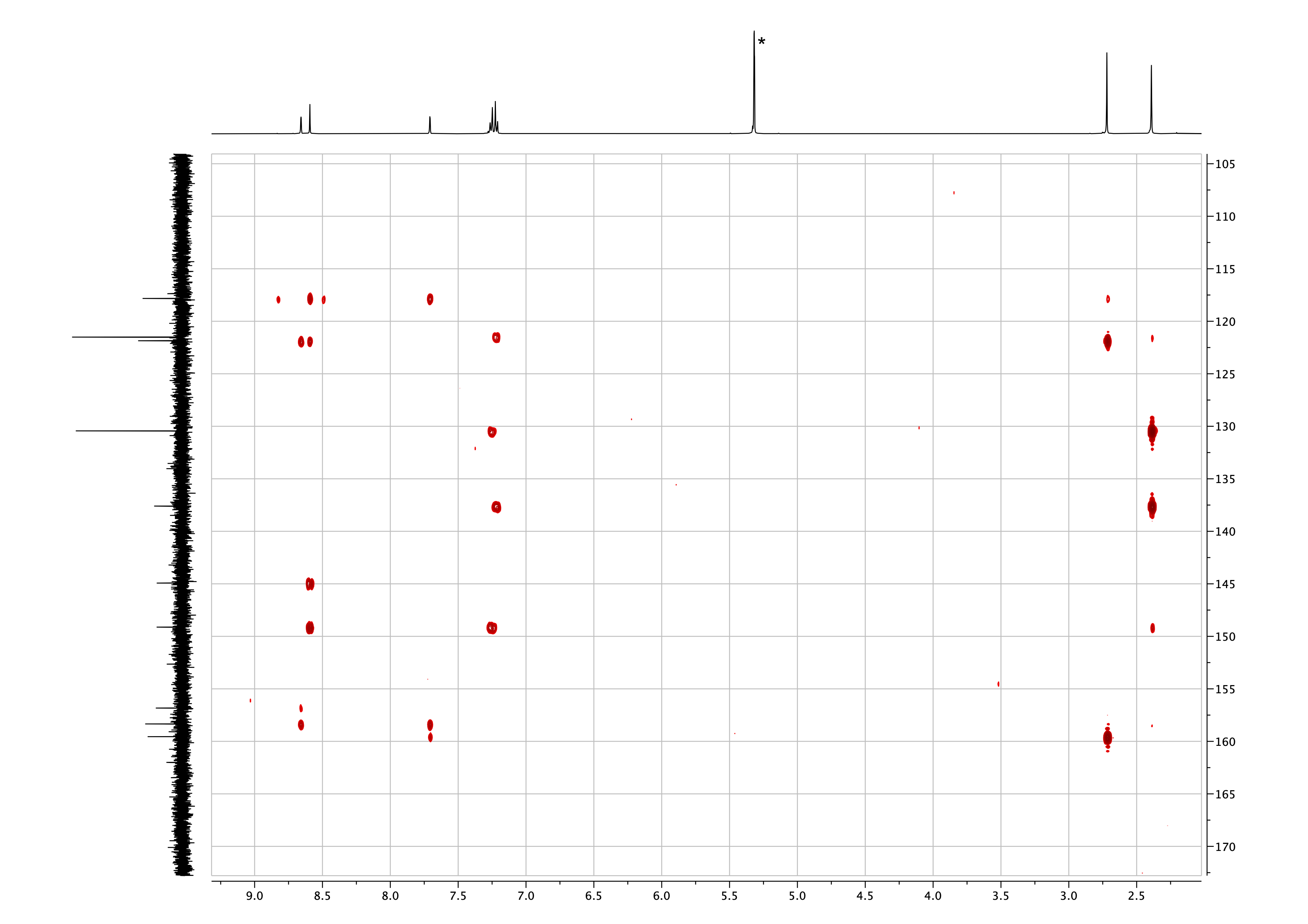


Figure S10. HMBC of ligand **2** (500 MHz ^1^H, 126 MHz ^13^C, CD_2_Cl_2_, 298 K). * = residual CHDCl_2_.


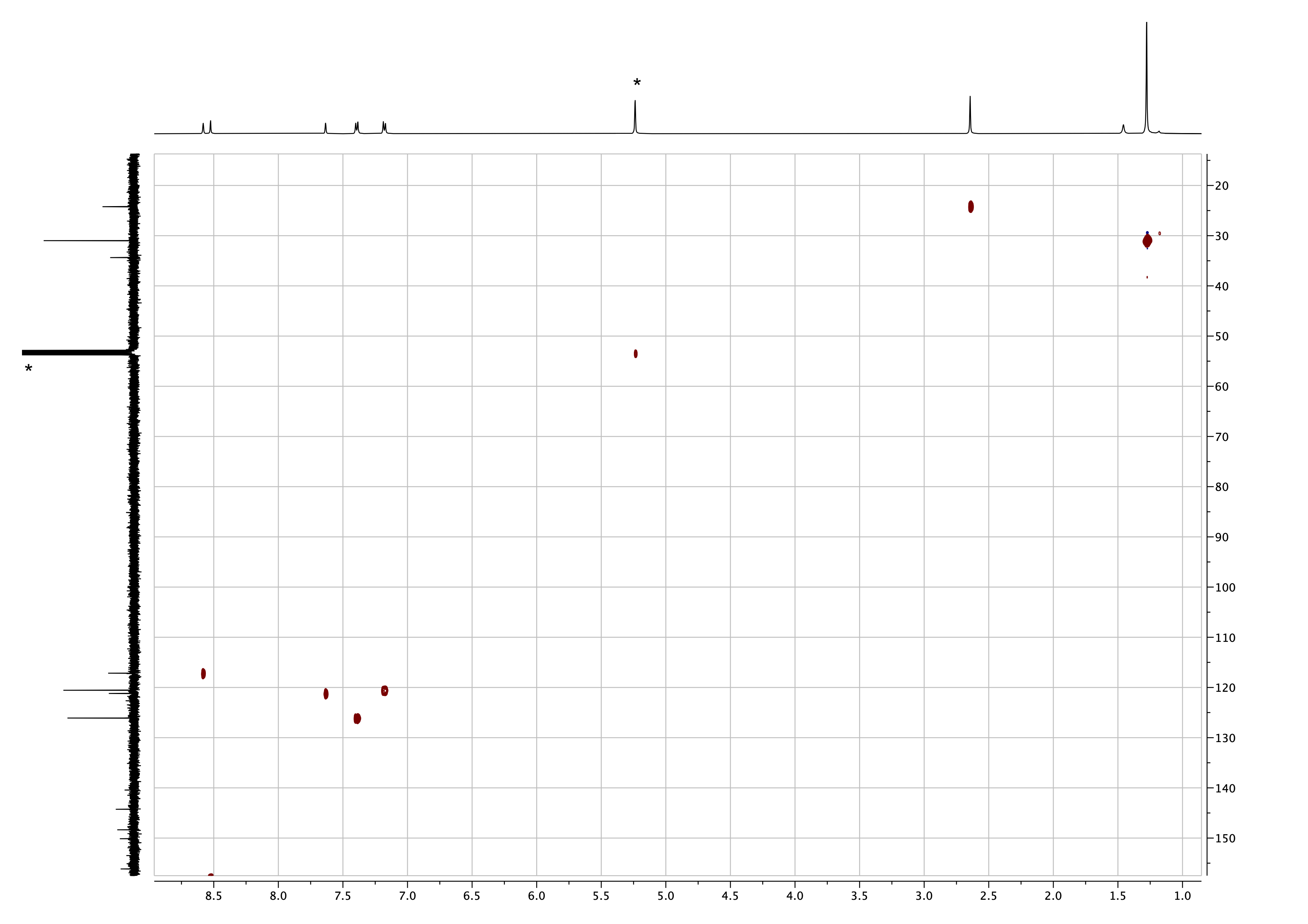


Figure S11. HMQC of ligand **3** (500 MHz ^1^H, 126 MHz ^13^C, CD_2_Cl_2_, 298 K). * = residual CHDCl_2_ or CDCl_3_.


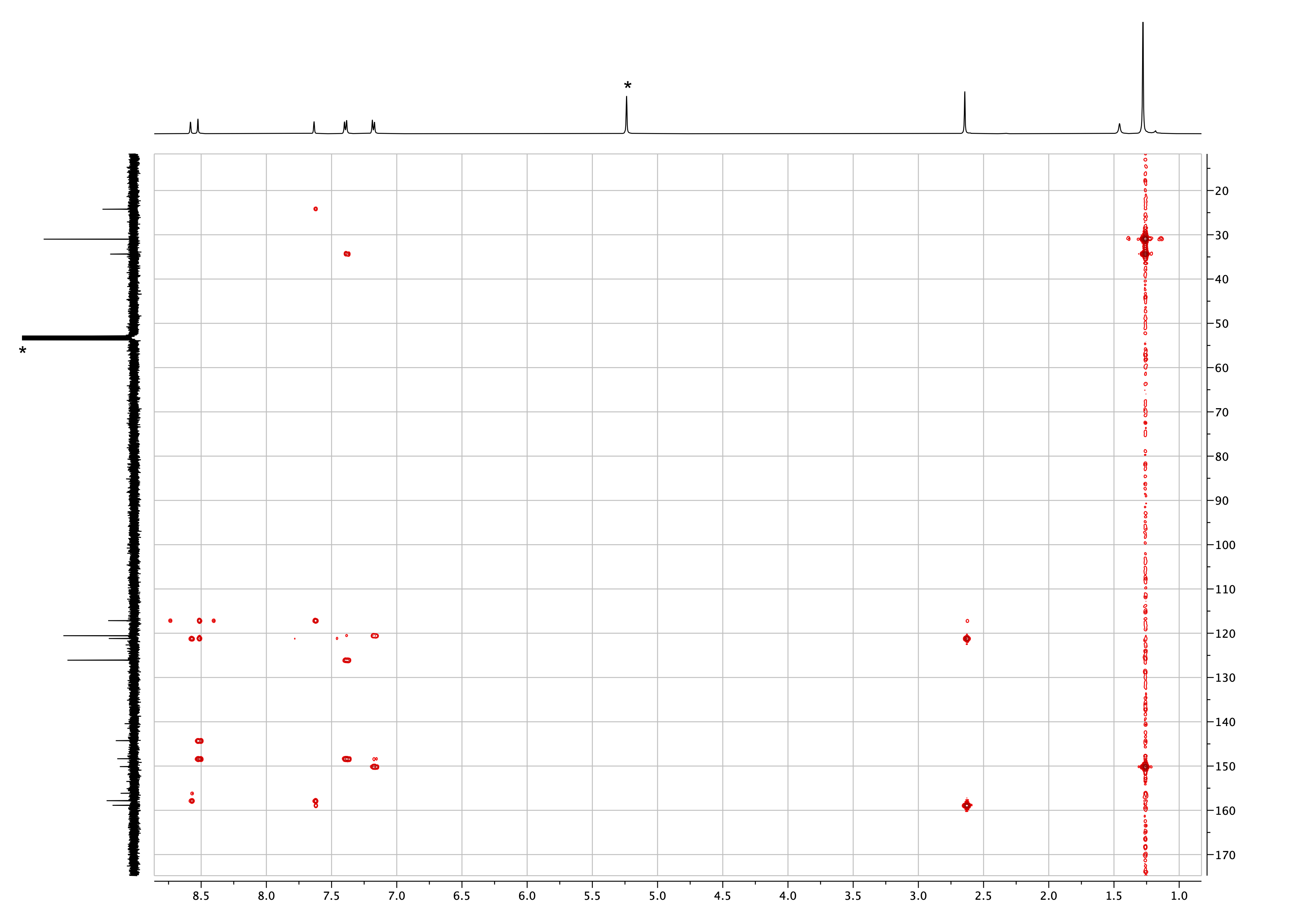


Figure S12. HMBC of ligand **3** (500 MHz ^1^H, 126 MHz ^13^C, CD_2_Cl_2_, 298 K). * = residual CHDCl_2_.


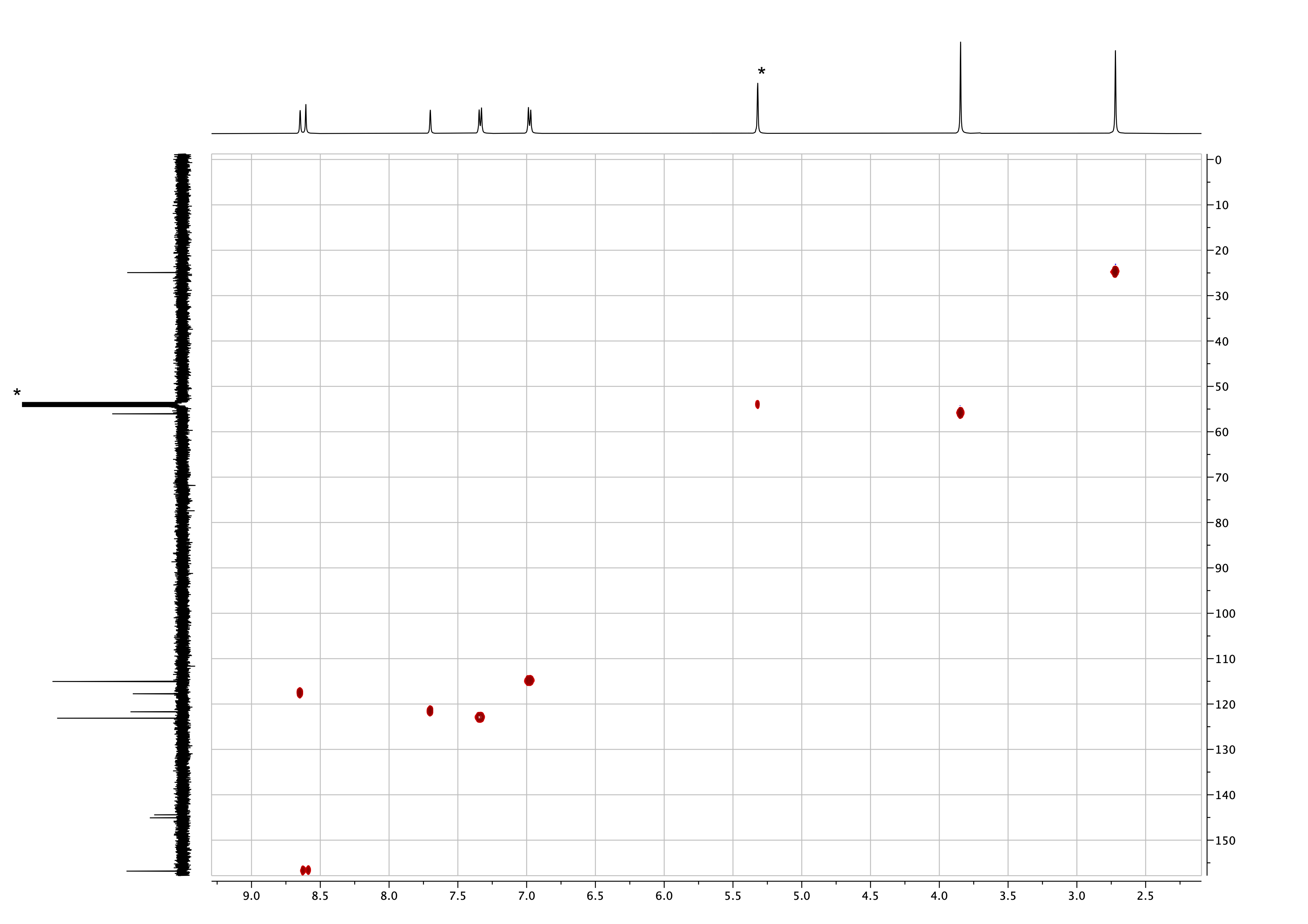


Figure S13. HMQC of ligand **4** (500 MHz ^1^H, 126 MHz ^13^C, CD_2_Cl_2_, 298 K). * = residual CHDCl_2_ or CDCl_3_.


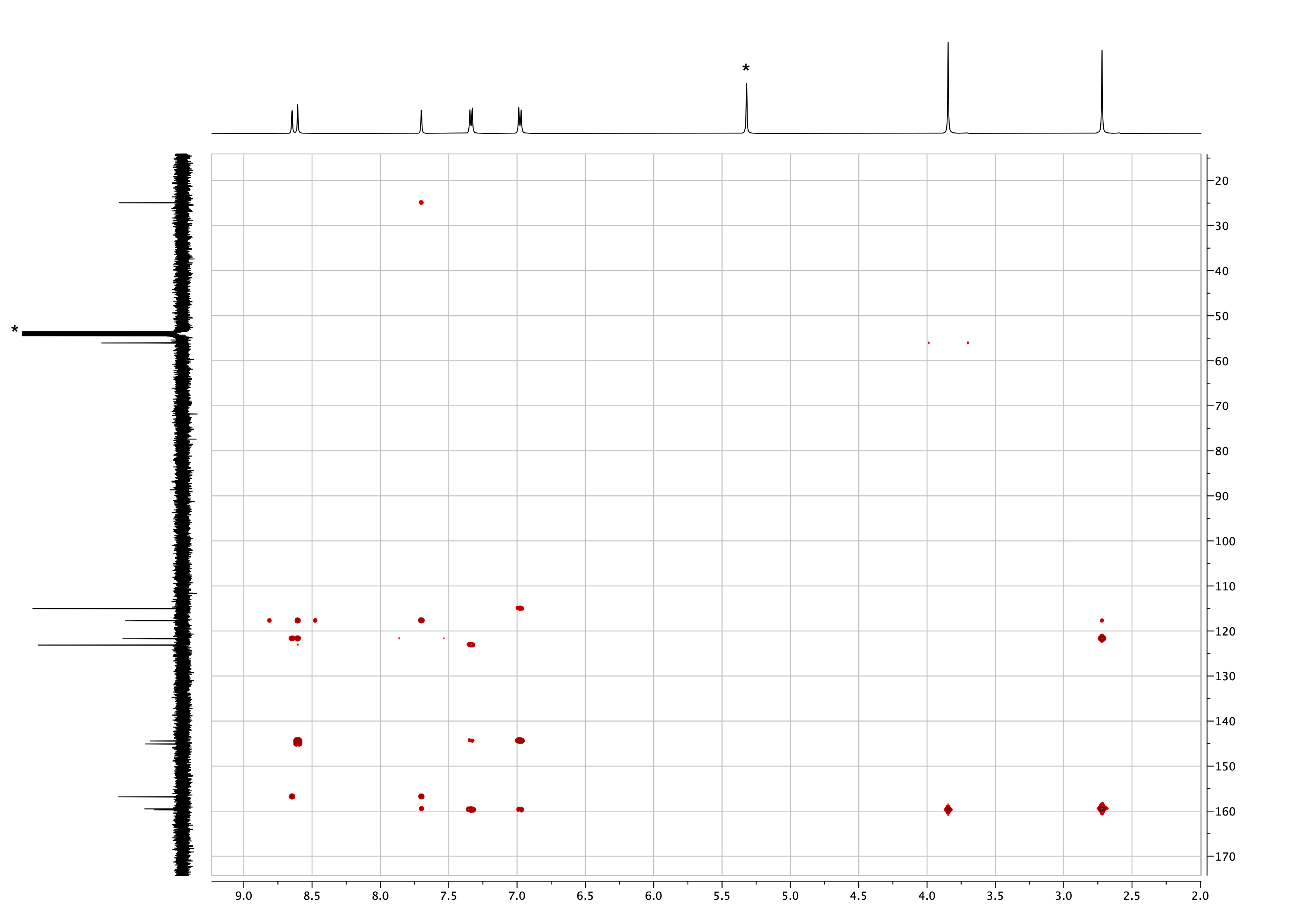


Figure S14. HMBC of ligand **4** (500 MHz ^1^H, 126 MHz ^13^C, CD_2_Cl_2_, 298 K). * = residual CHDCl_2_ or CDCl_3_.


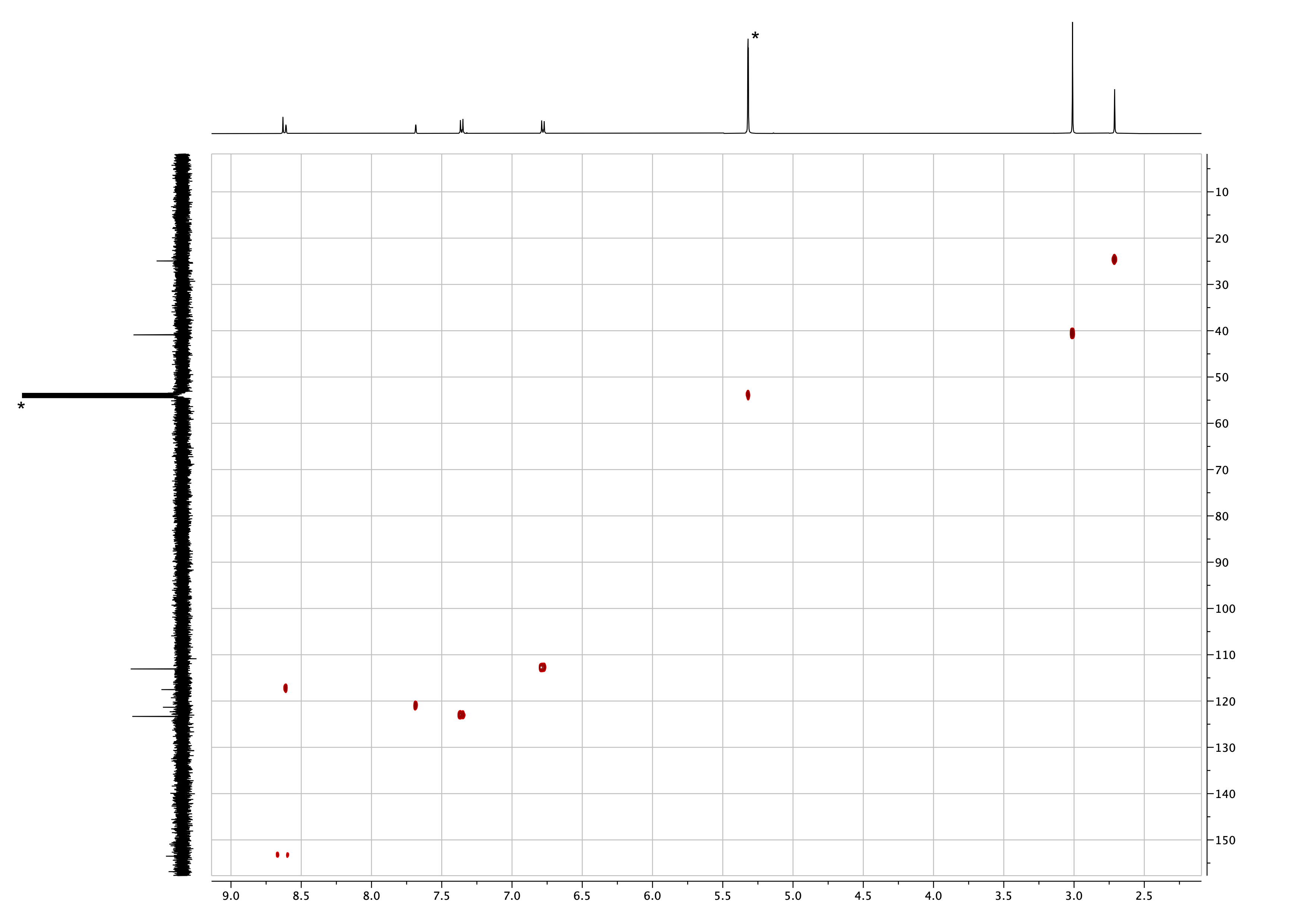


Figure S15. HMQC of ligand **5** (500 MHz ^1^H, 126 MHz ^13^C, CD_2_Cl_2_, 298 K). * = residual CHDCl_2_ or CDCl_3_.


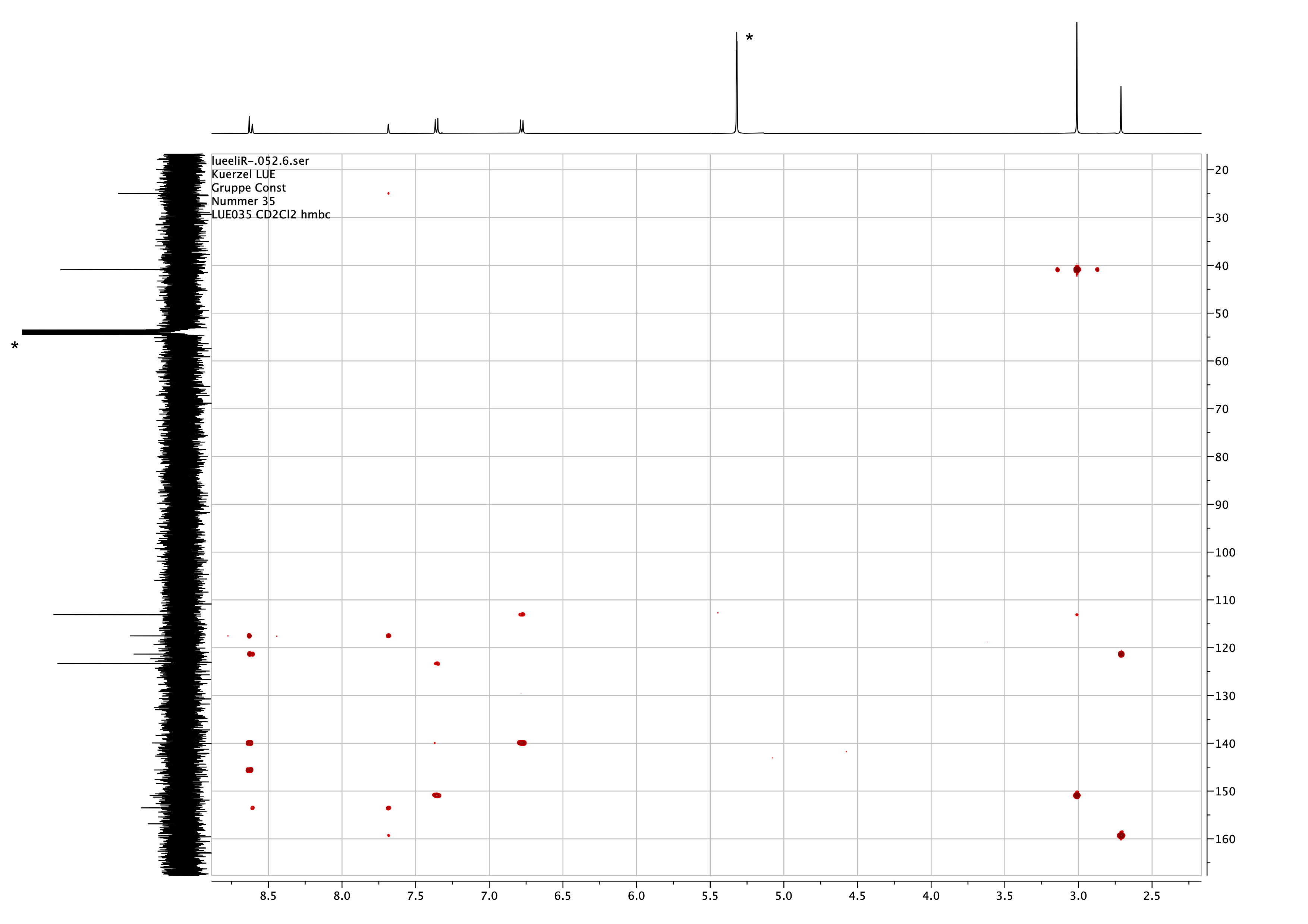


Figure S16. HMBC of ligand **5** (500 MHz ^1^H, 126 MHz ^13^C, CD_2_Cl_2_, 298 K). * = residual CHDCl_2_ or CDCl_3_.


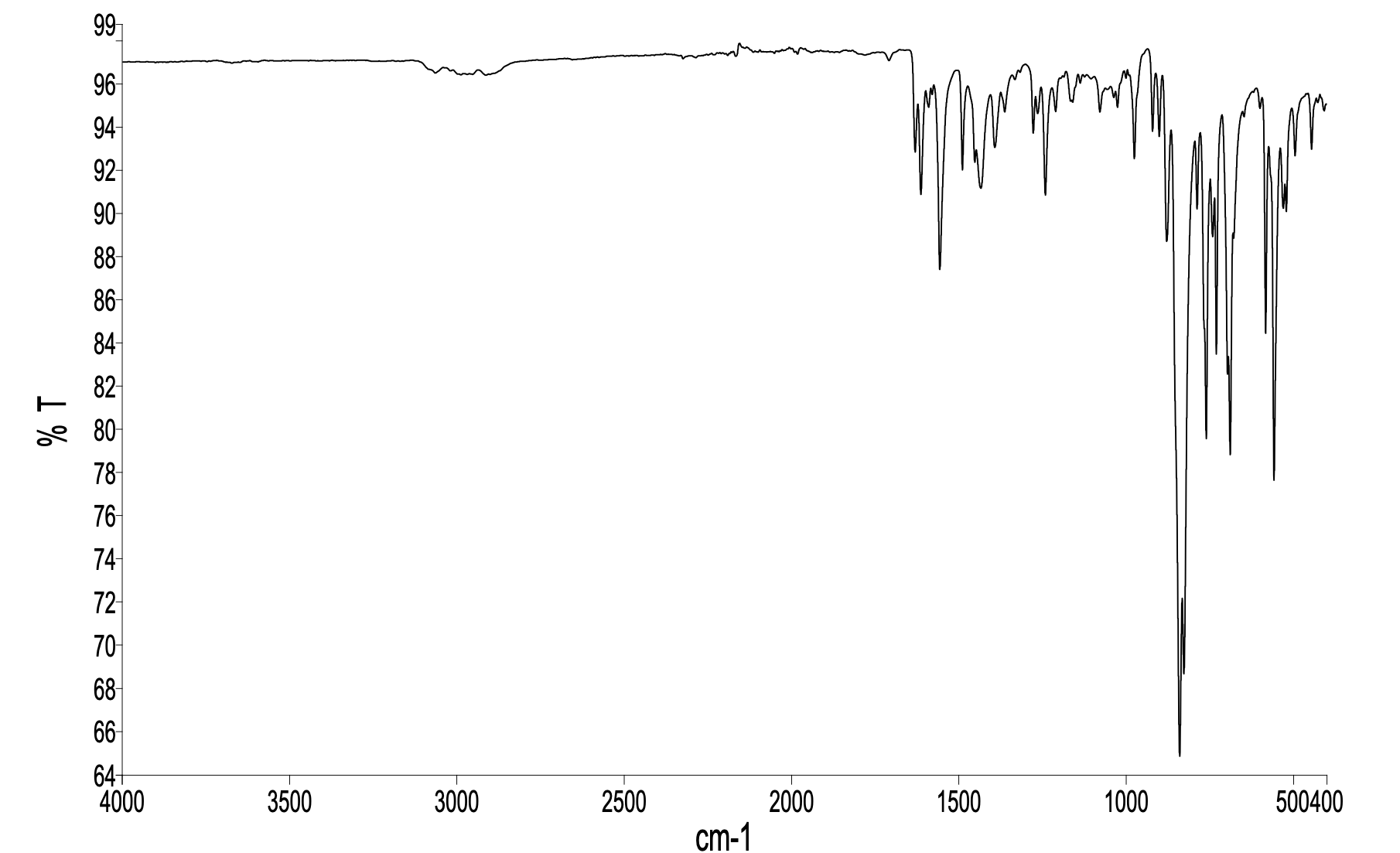


Figure S17. Solid-state FT-IR spectrum of [Cu(**1**)_2_][PF_6_].


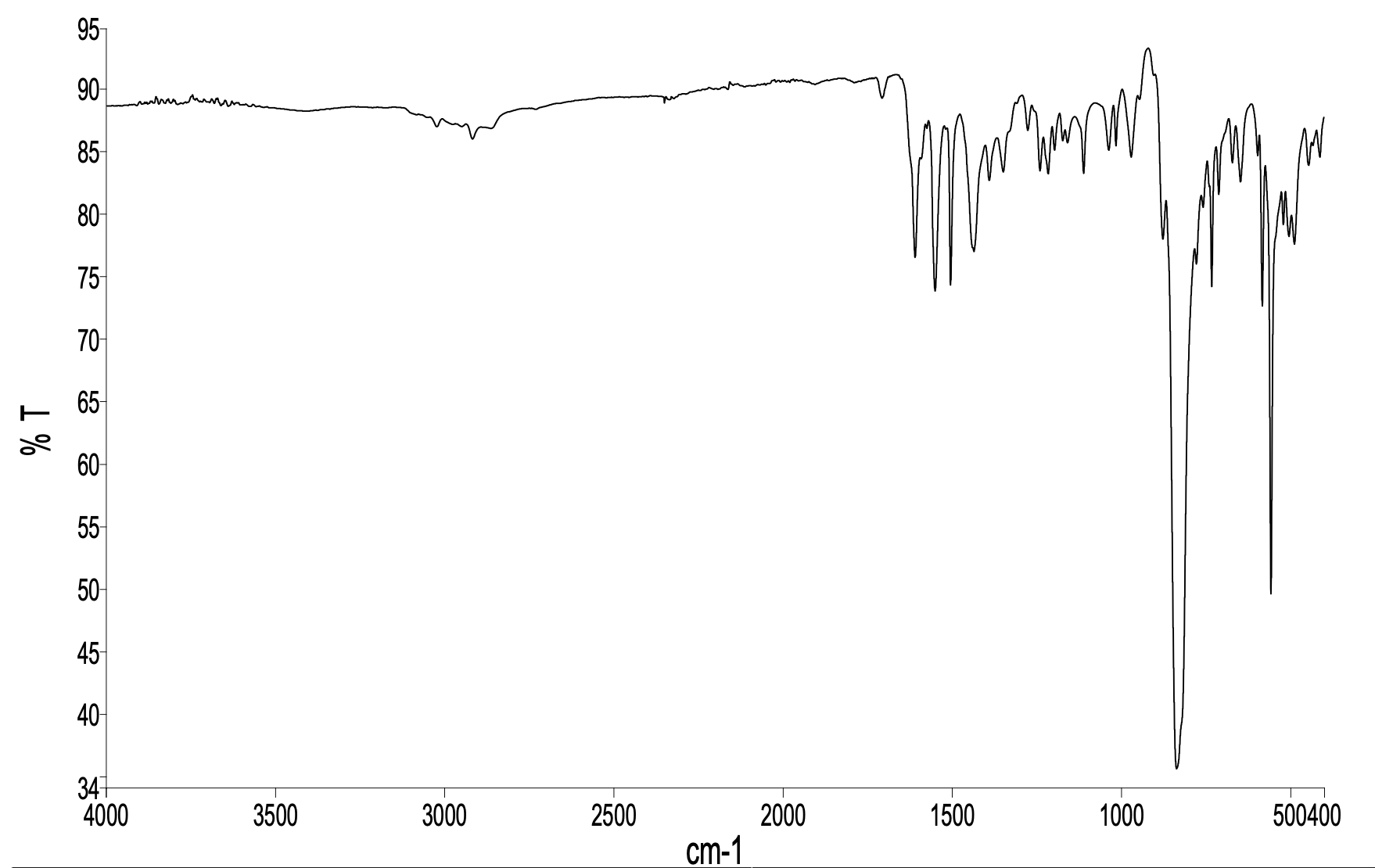


Figure S18. Solid-state FT-IR spectrum of [Cu(**2**)_2_][PF_6_].


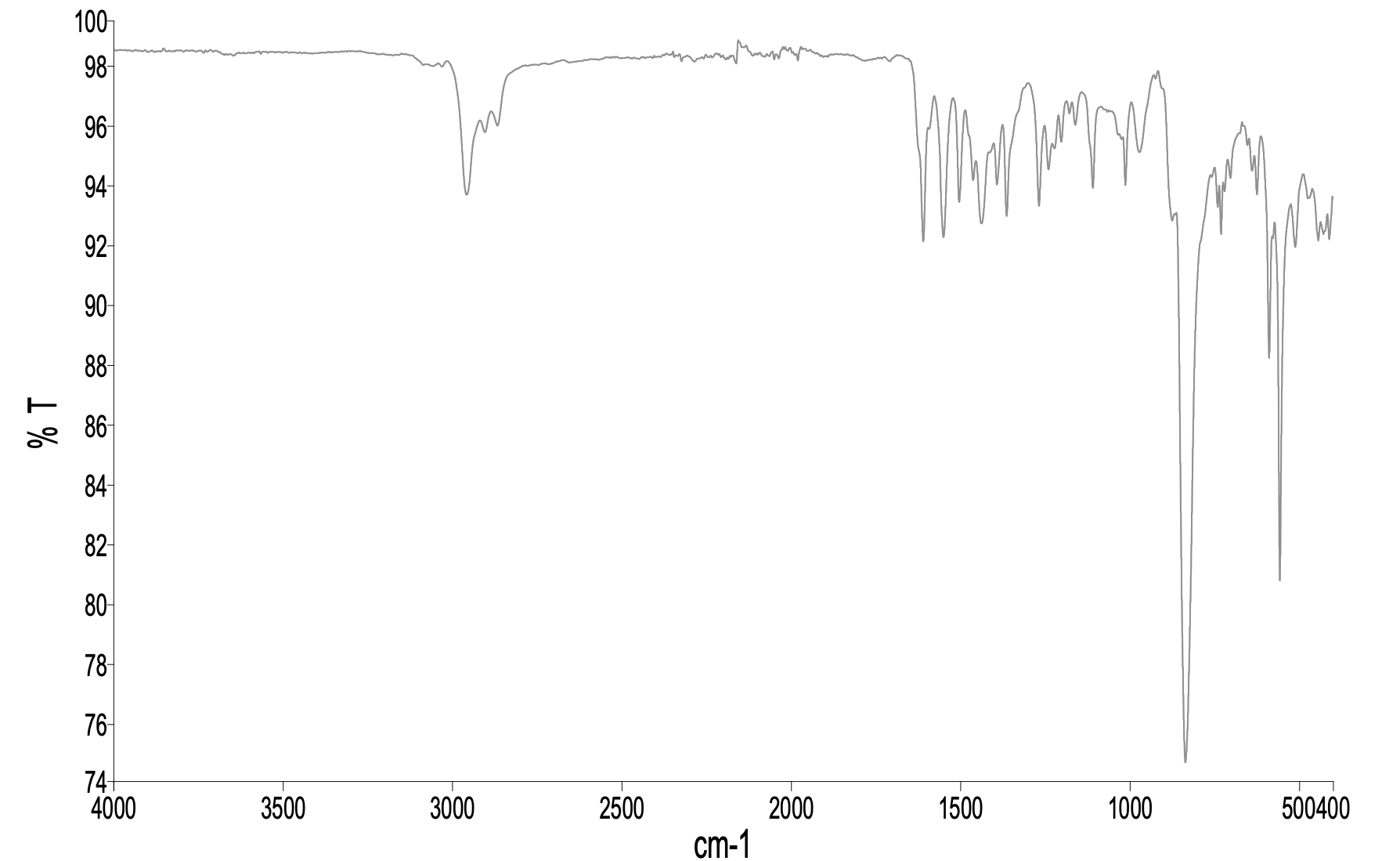


Figure S19. Solid-state FT-IR spectrum of [Cu(**3**)_2_][PF_6_].


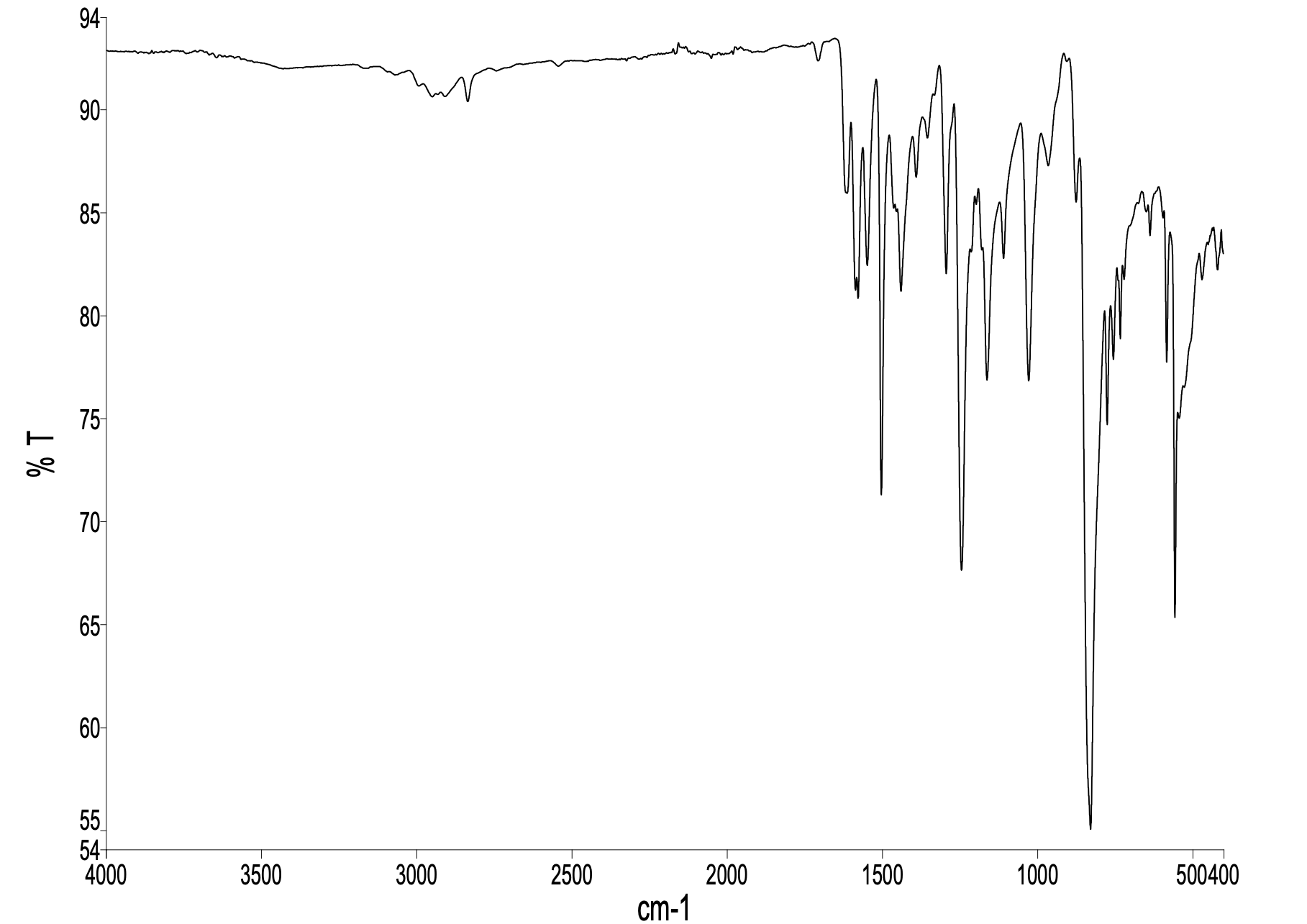


Figure S20. Solid-state FT-IR spectrum of [Cu(**4**)_2_][PF_6_].


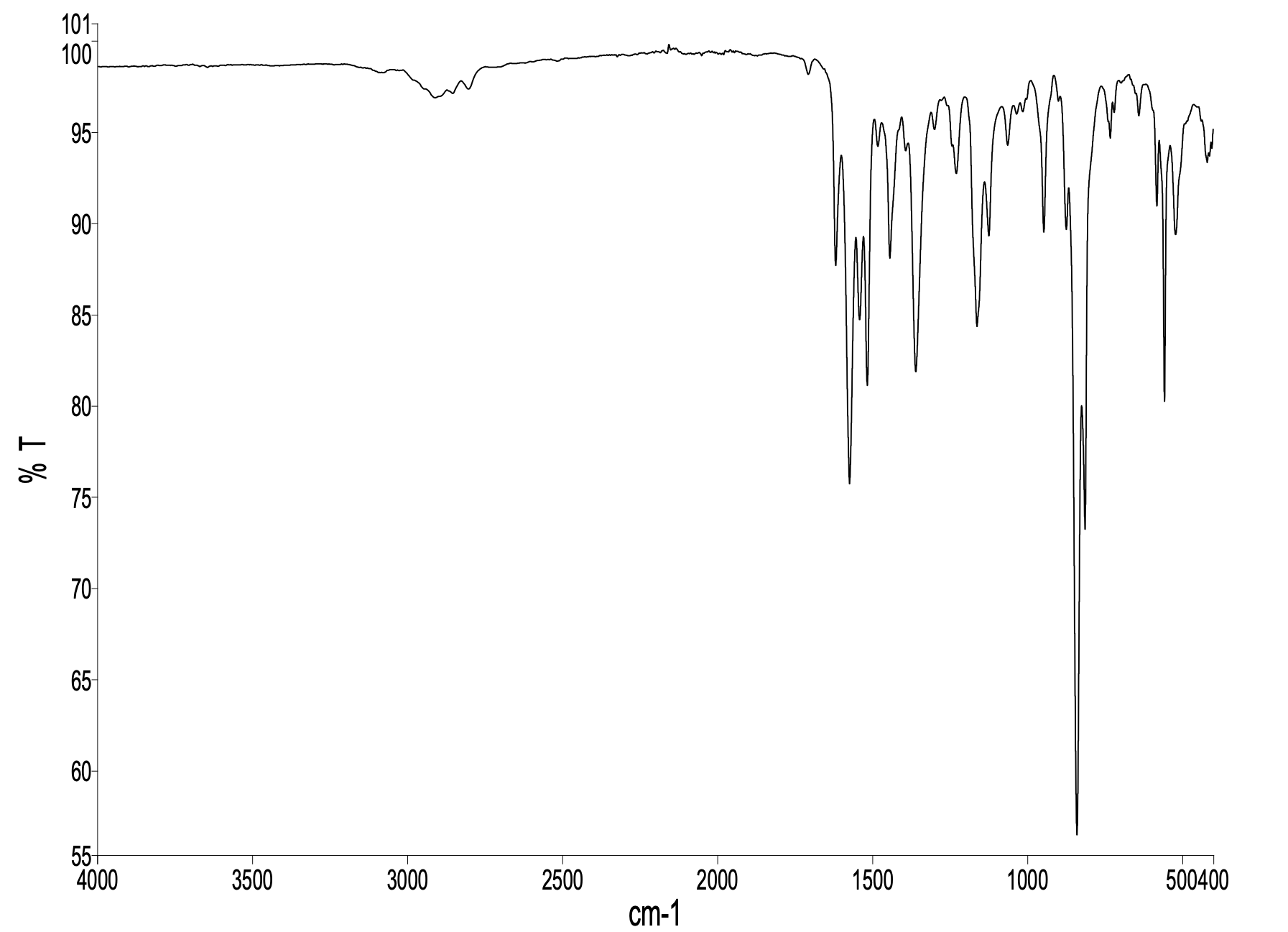


Figure S21. Solid-state FT-IR spectrum of [Cu(**5**)_2_][PF_6_].


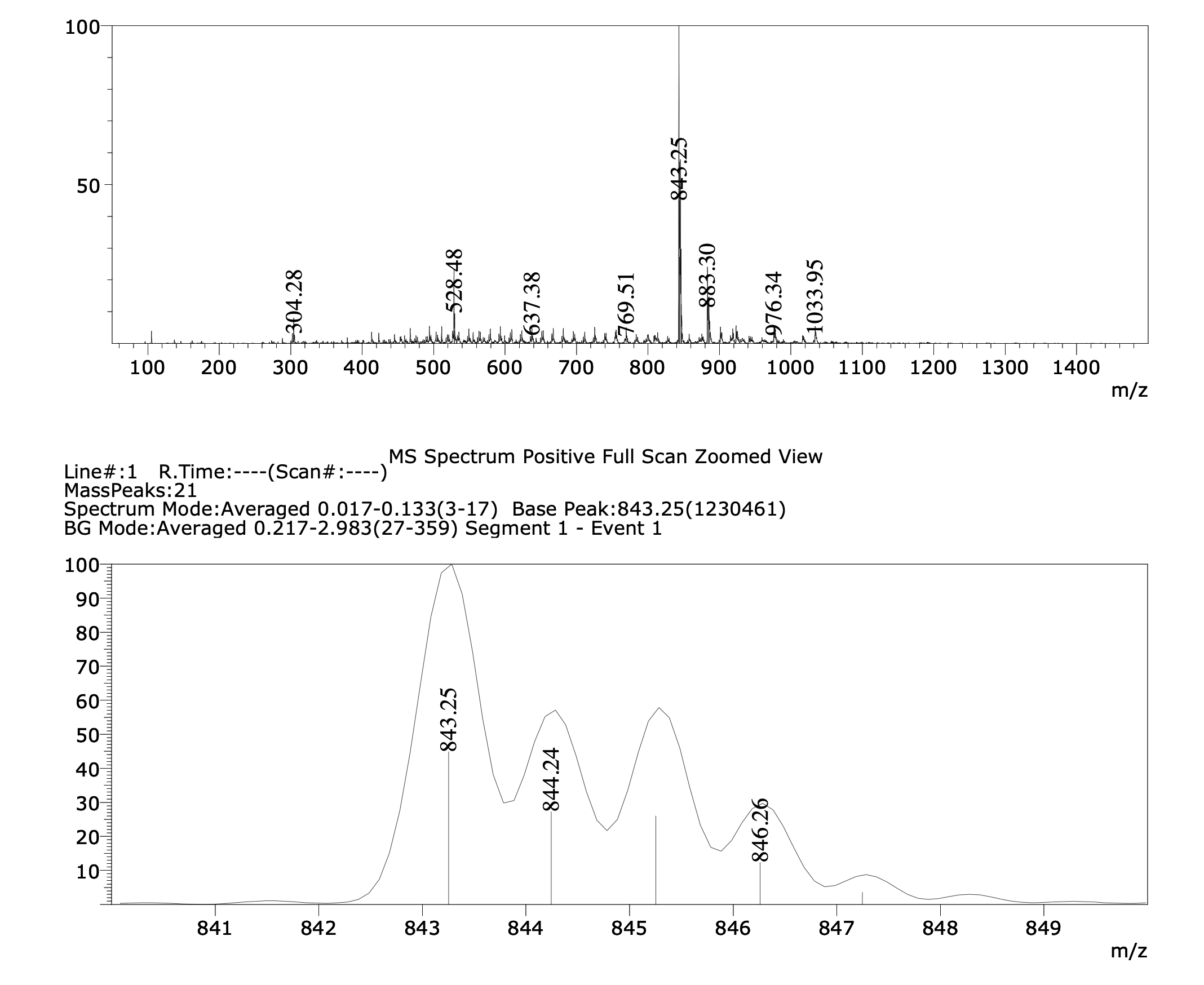


Figure S22. Electrospray mass spectrum of [Cu(**1**)_2_][PF_6_].


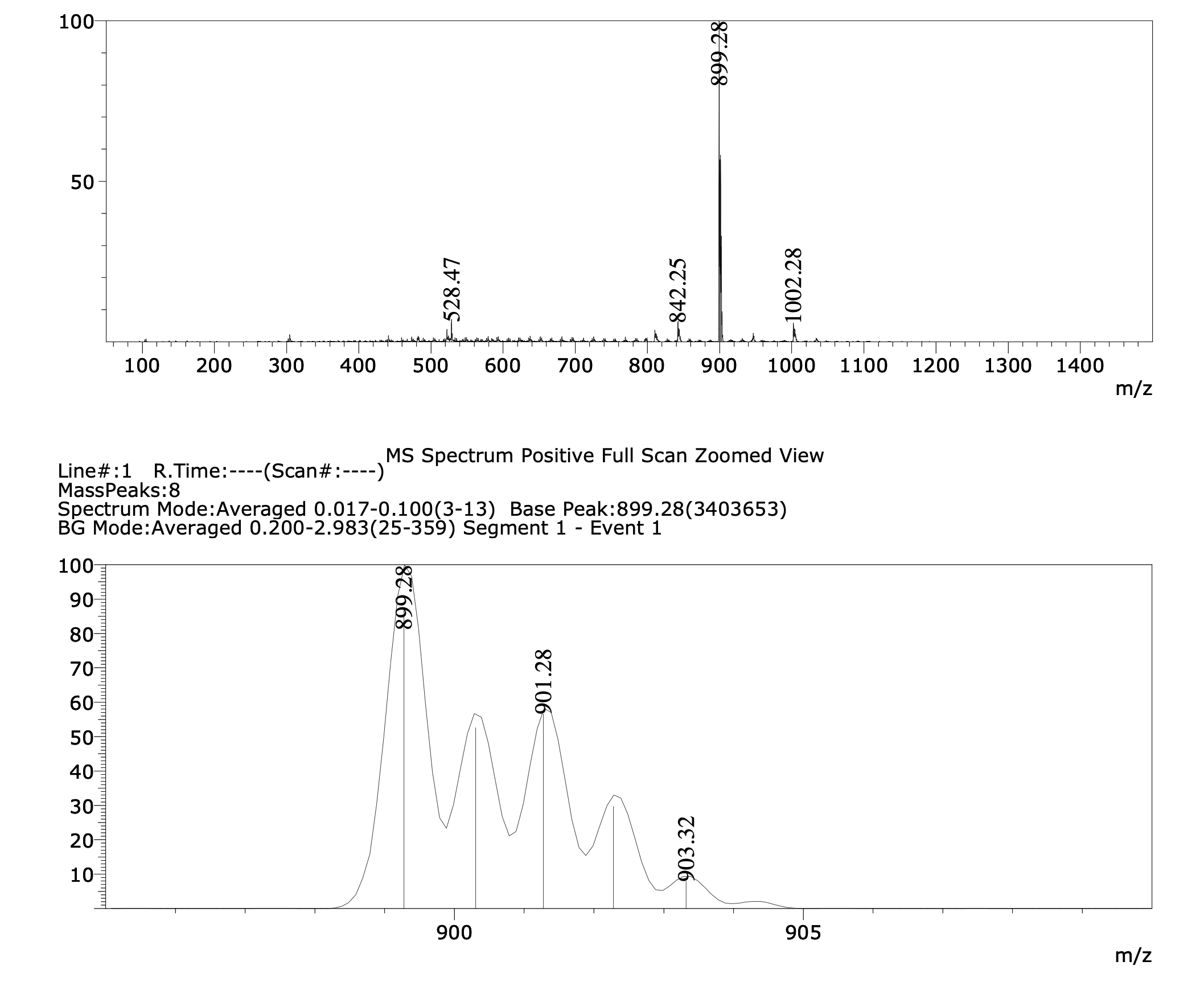


Figure S23. Electrospray mass spectrum of [Cu(**2**)_2_][PF_6_].

Figure S24. Electrospray mass spectrum of [Cu(**3**)_2_][PF_6_].


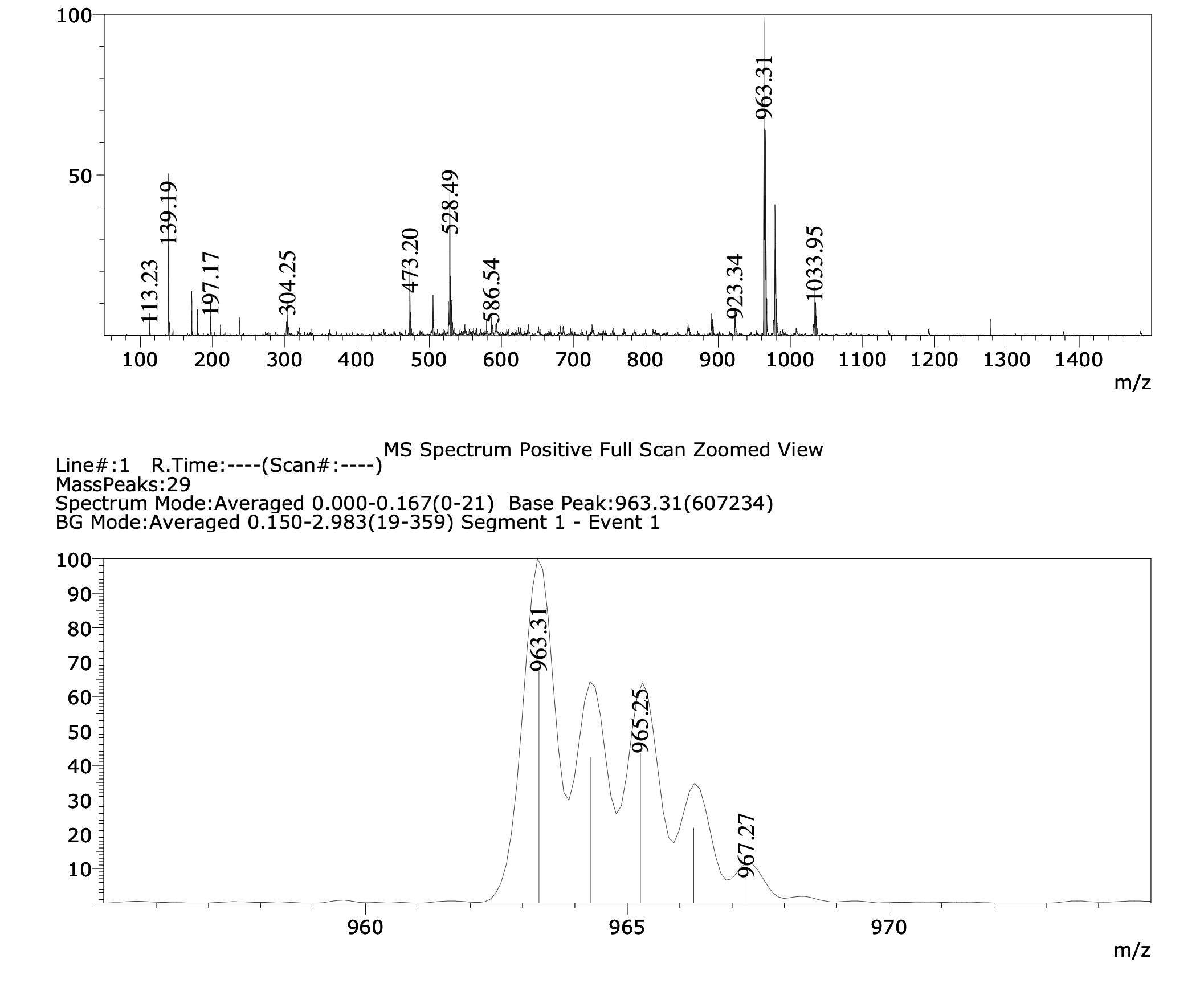


Figure S25. Electrospray mass spectrum of [Cu(**4**)_2_][PF_6_].


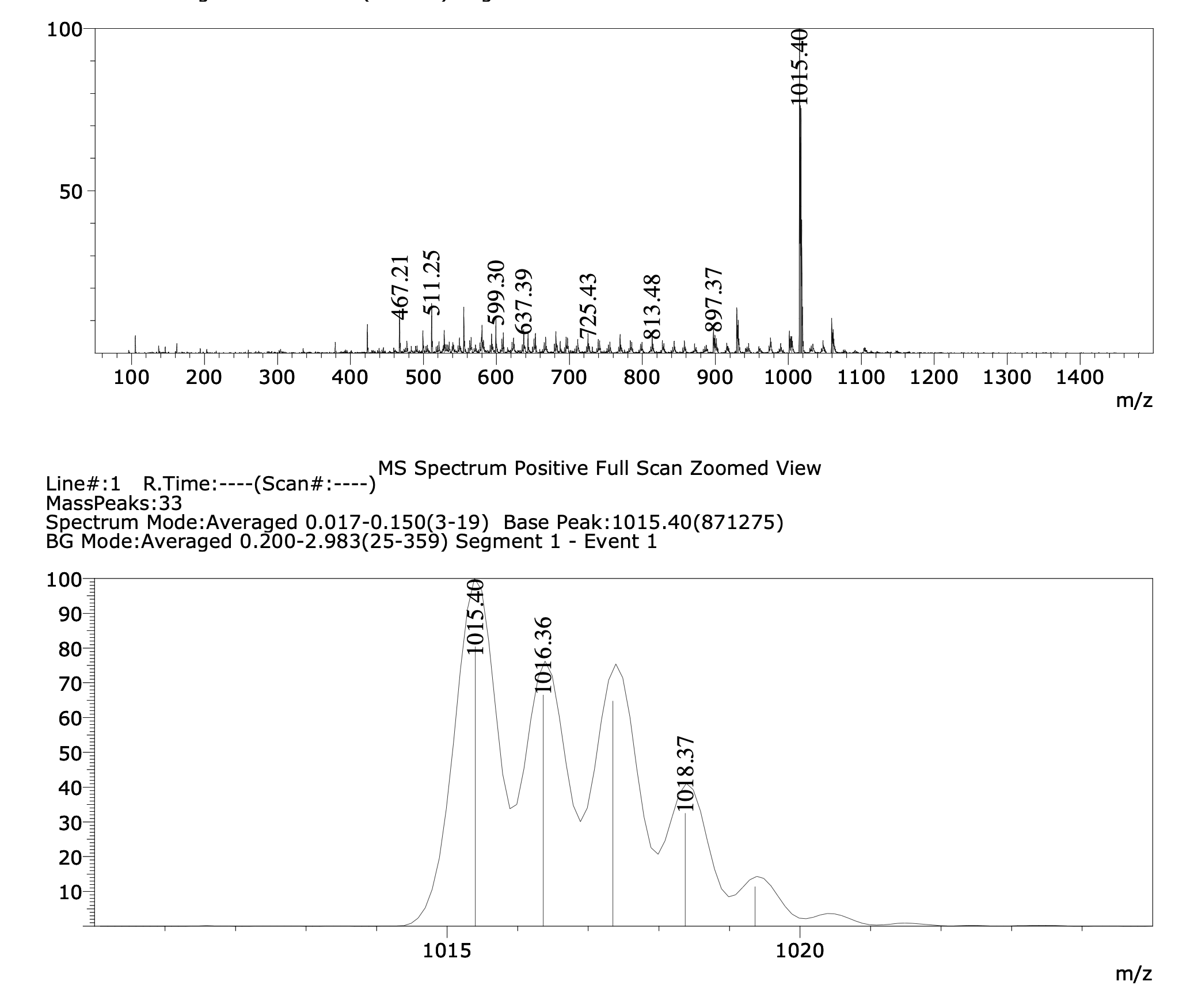


Figure S26. Electrospray mass spectrum of [Cu(**5**)_2_][PF_6_].


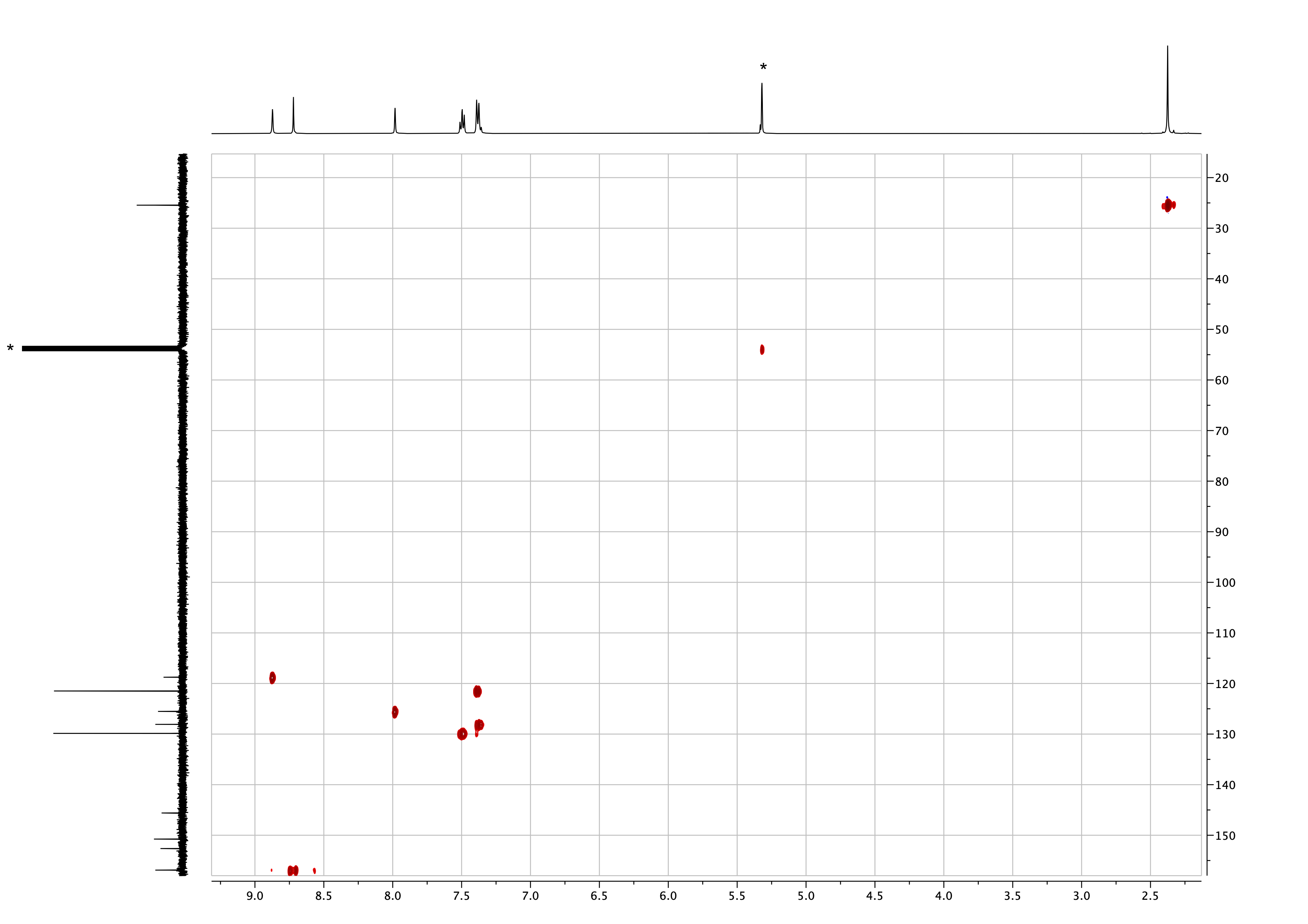


Figure S27. HMQC of complex [Cu(**1**)_2_][PF_6_] (500 MHz ^1^H, 126 MHz ^13^C, CD_2_Cl_2_, 298 K). * = residual CHDCl_2_.


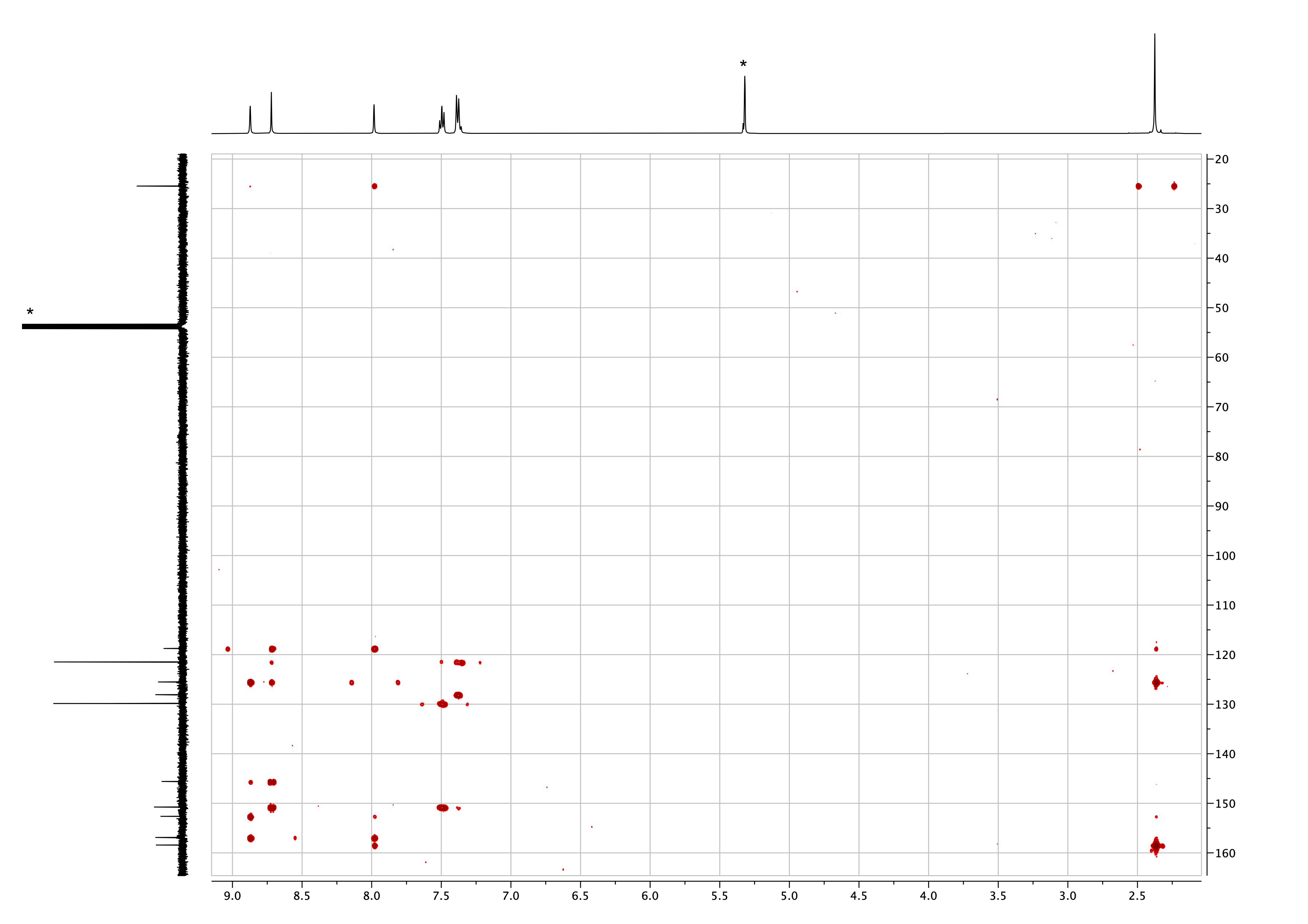


Figure S28. HMBC of complex [Cu(**1**)_2_][PF_6_] (500 MHz ^1^H, 126 MHz ^13^C, CD_2_Cl_2_, 298 K). * = residual CHDCl_2_.


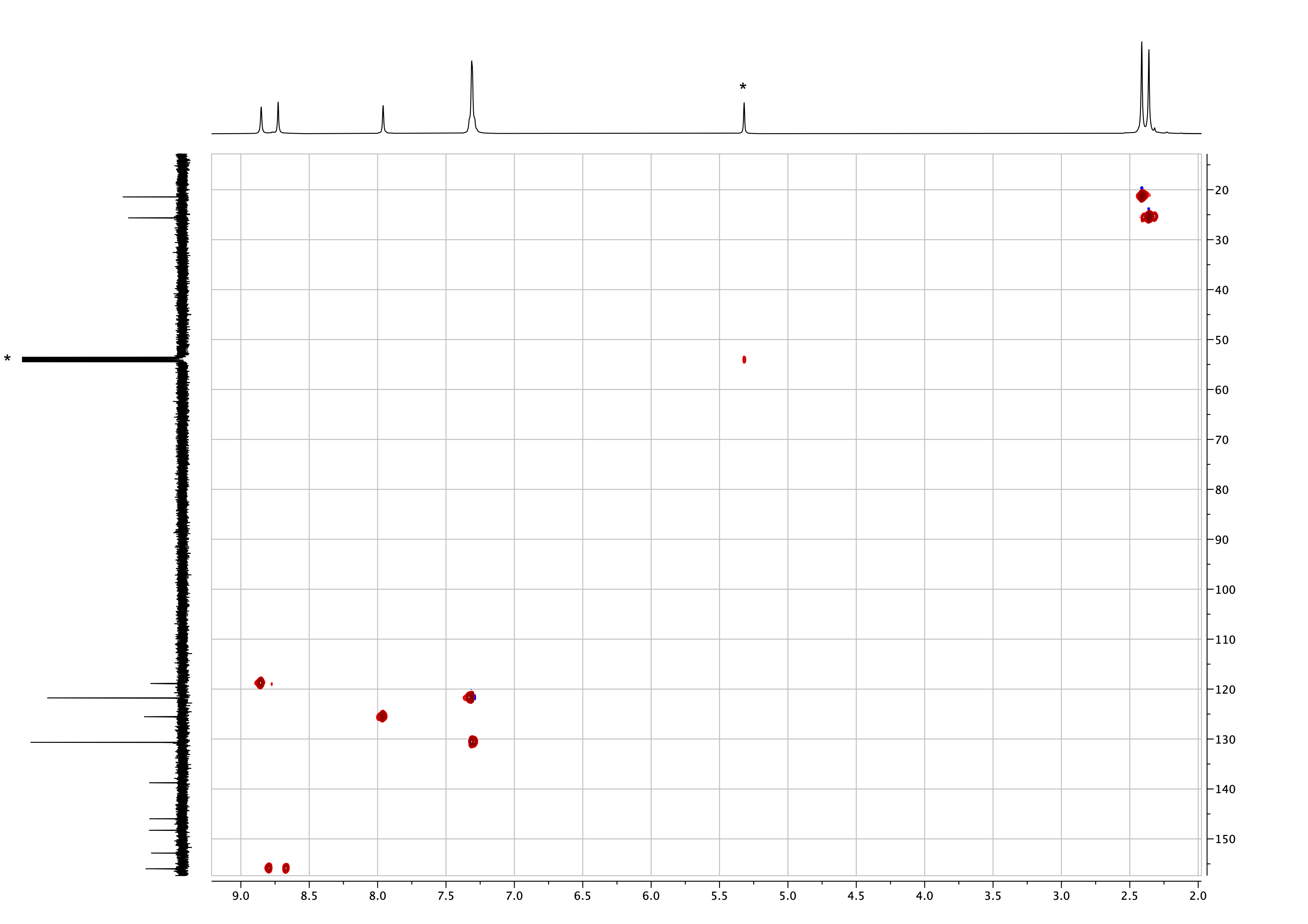


Figure S29. HMQC of complex [Cu(**2**)_2_][PF_6_] (500 MHz ^1^H, 126 MHz ^13^C, CD_2_Cl_2_, 298 K). * = residual CHDCl_2_.


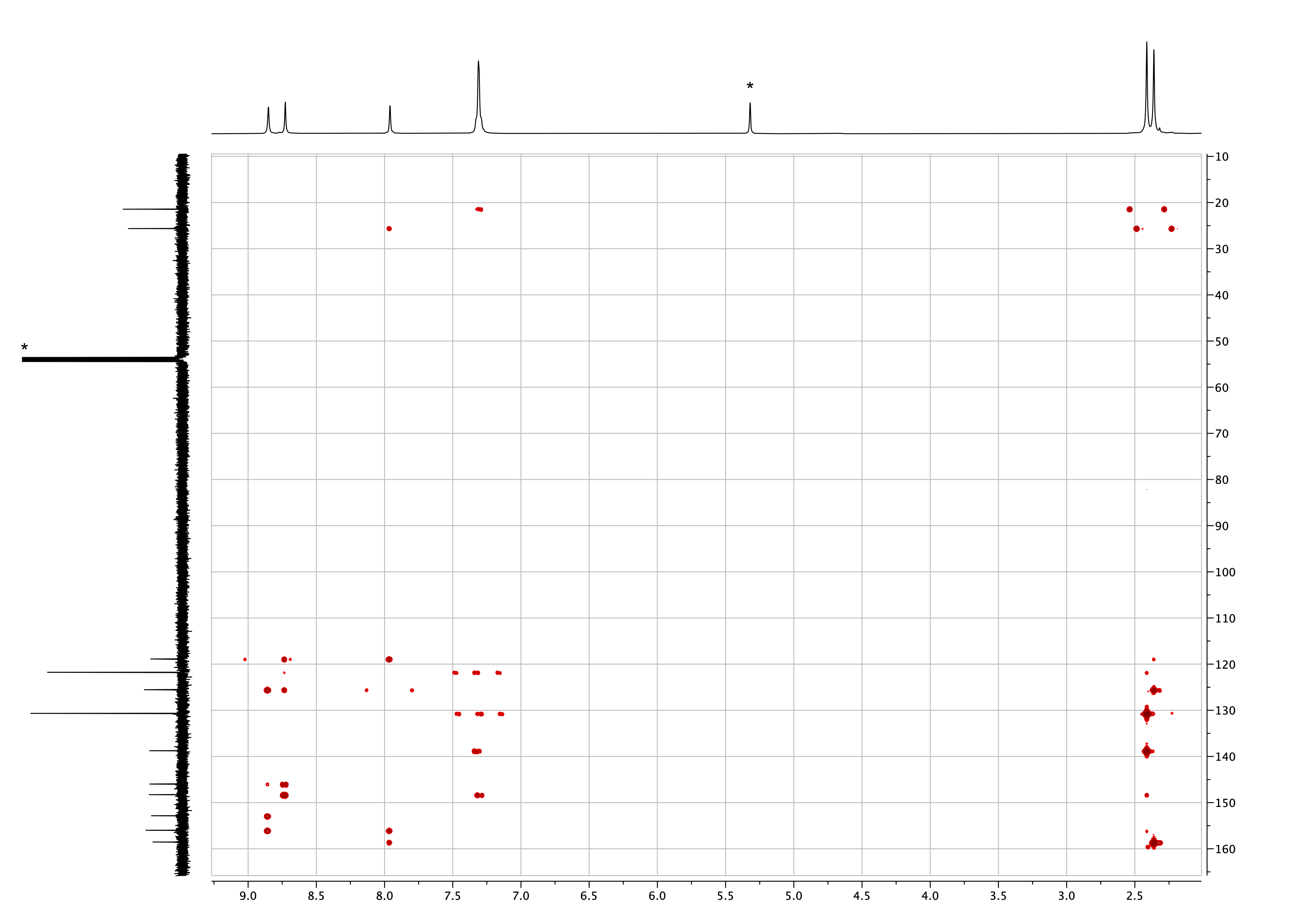
Figure S30. HMBC of complex [Cu(**2**)_2_][PF_6_] (500 MHz ^1^H, 126 MHz ^13^C, CD_2_Cl_2_, 298 K). * = residual CHDCl_2_.


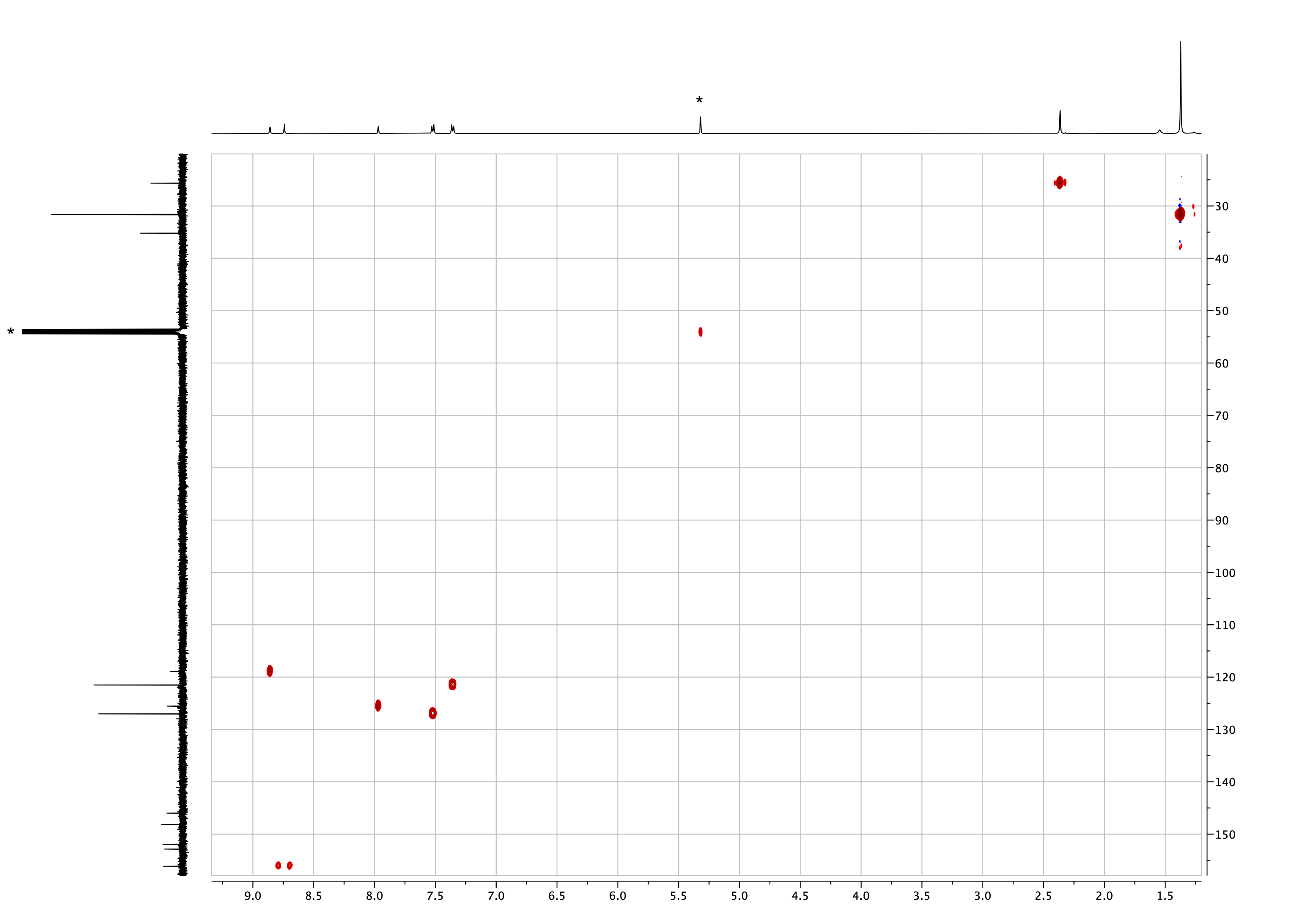


Figure S31. HMQC of complex [Cu(**3**)_2_][PF_6_] (500 MHz ^1^H, 126 MHz ^13^C, CD_2_Cl_2_, 298 K). * = residual CHDCl_2_.


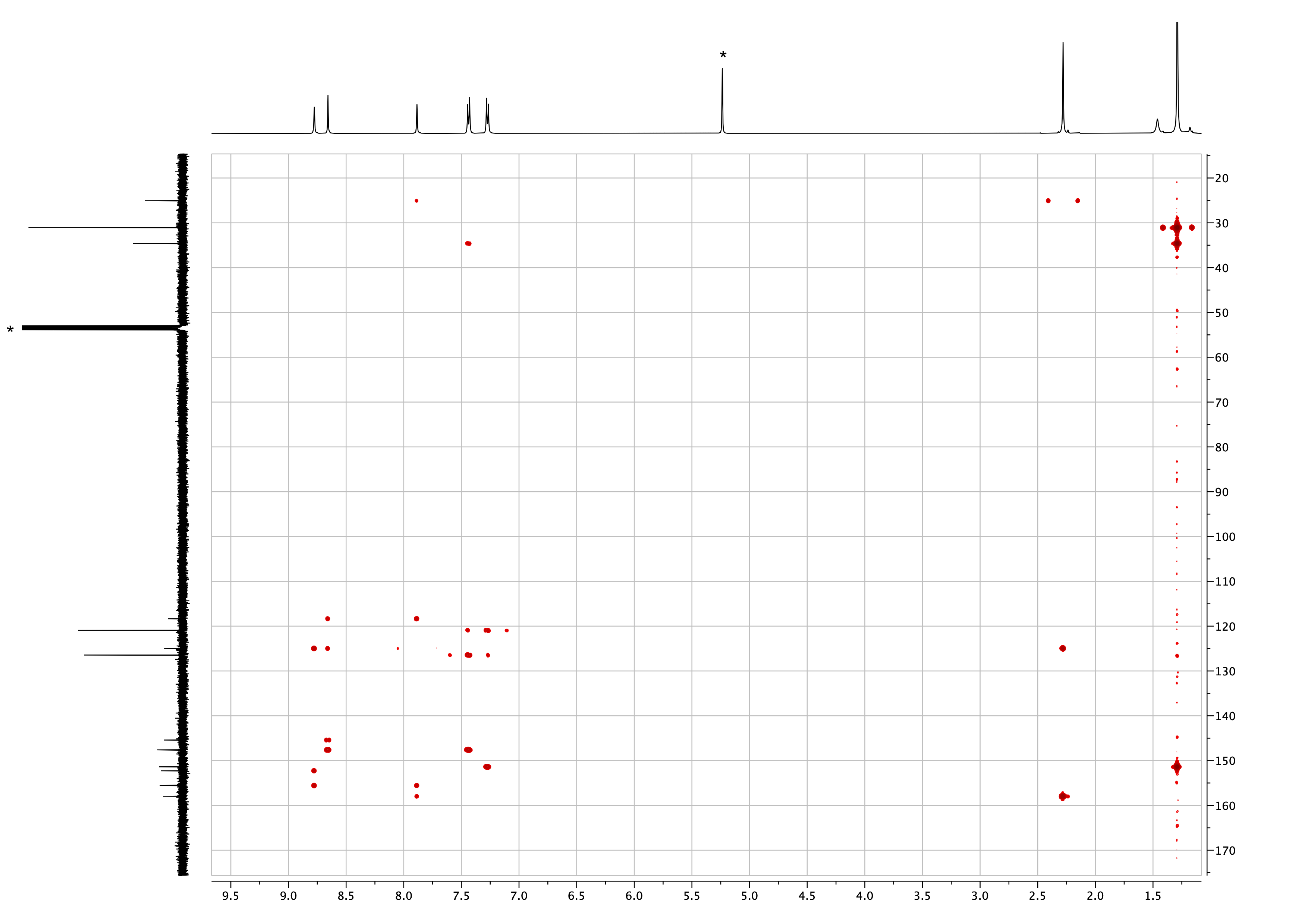


Figure S32. HMBC of complex [Cu(**3**)_2_][PF_6_] (500 MHz ^1^H, 126 MHz ^13^C, CD_2_Cl_2_, 298 K). * = residual CHDCl_2_.


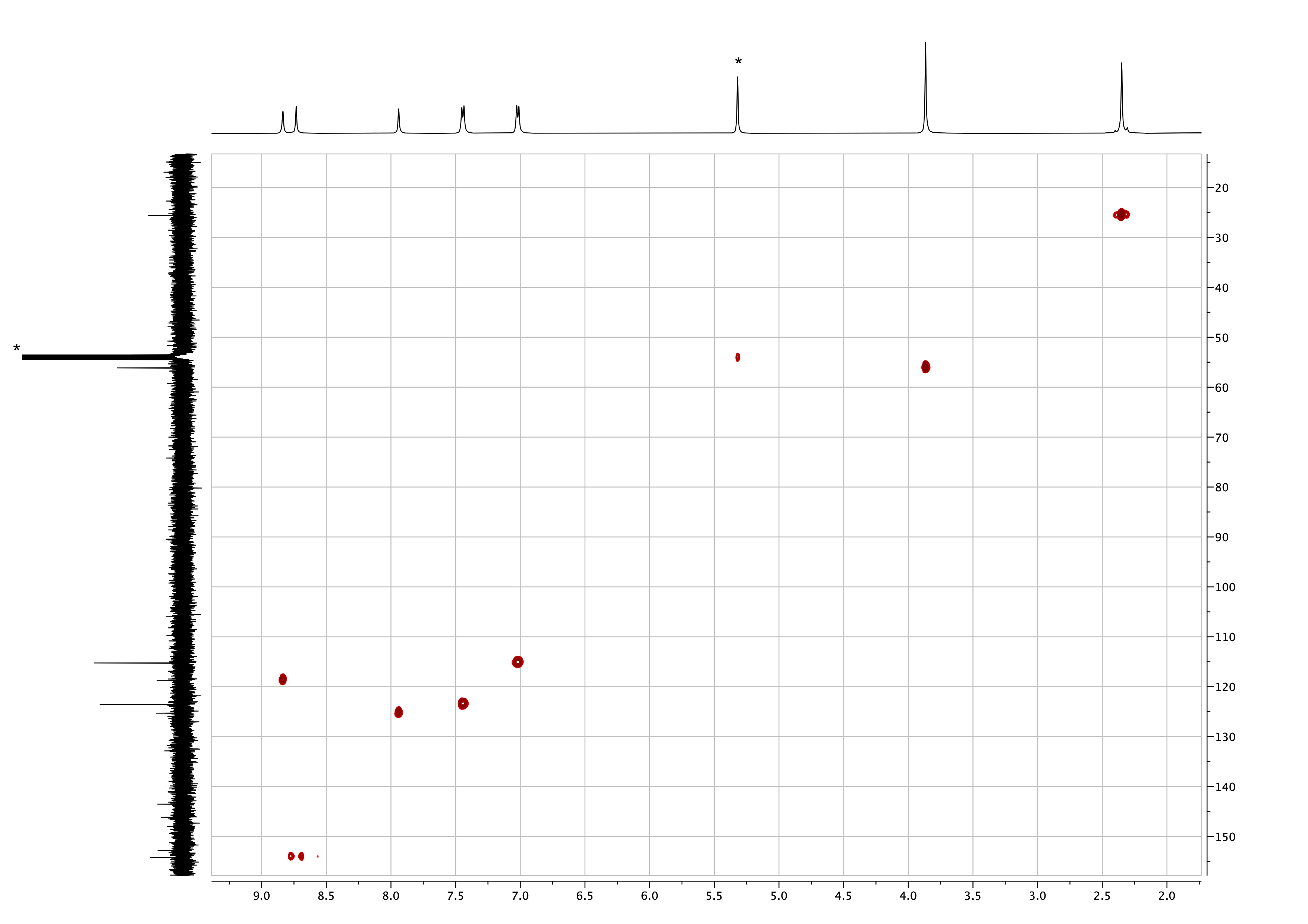


Figure S33. HMQC of complex [Cu(**4**)_2_][PF_6_] (500 MHz ^1^H, 126 MHz ^13^C, CD_2_Cl_2_, 298 K). * = residual CHDCl_2_.


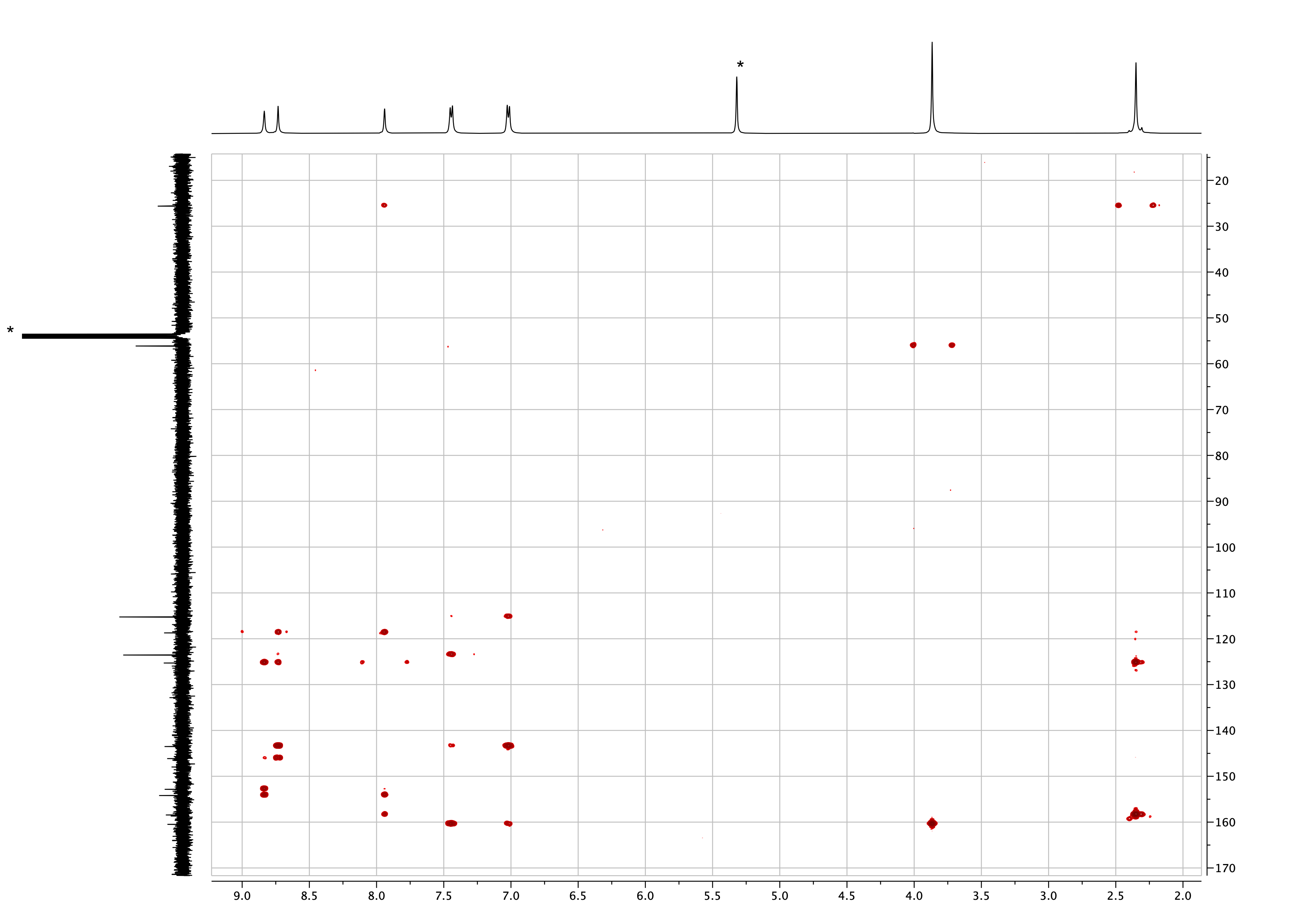


Figure S34. HMBC of complex [Cu(**4**)_2_][PF_6_] (500 MHz ^1^H, 126 MHz ^13^C, CD_2_Cl_2_, 298 K). * = residual CHDCl_2_.


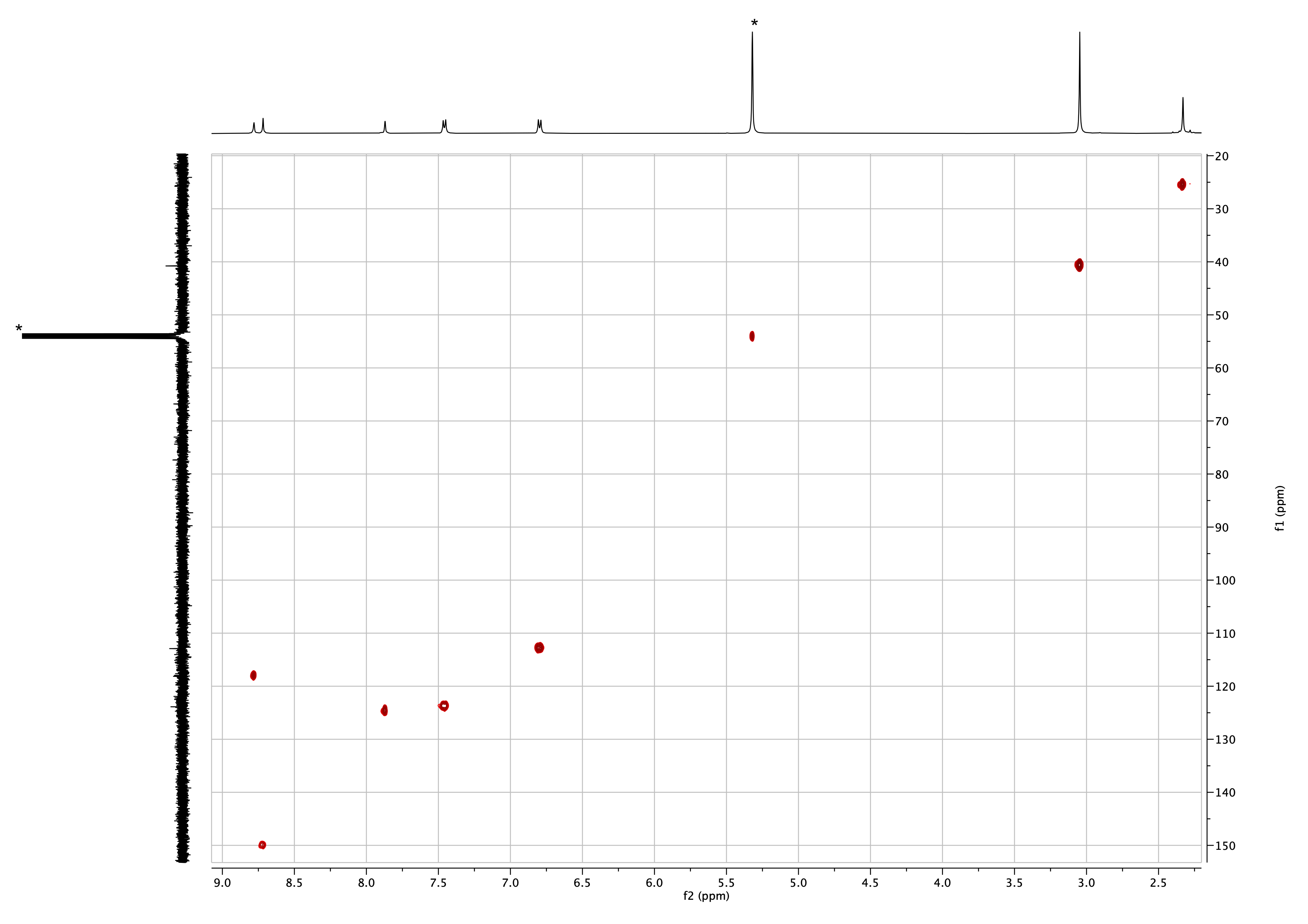


Figure S35. HMQC of complex [Cu(**5**)_2_][PF_6_] (500 MHz ^1^H, 126 MHz ^13^C, CD_2_Cl_2_, 298 K). * = residual CHDCl_2_.


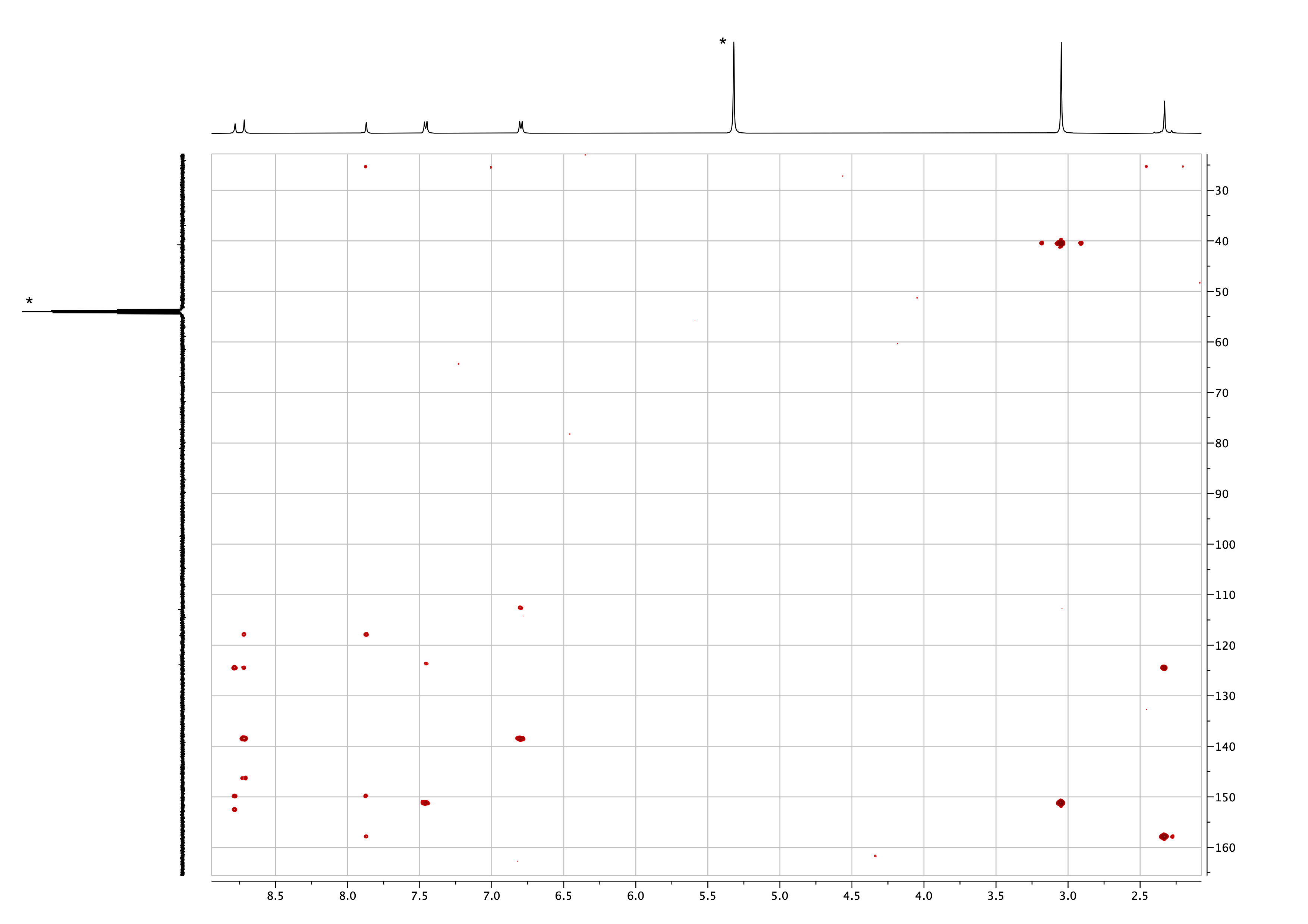
Figure S36. HMBC of complex [Cu(**5**)_2_][PF_6_] (500 MHz ^1^H, 126 MHz ^13^C, CD_2_Cl_2_, 298 K). * = residual CHDCl_2_.


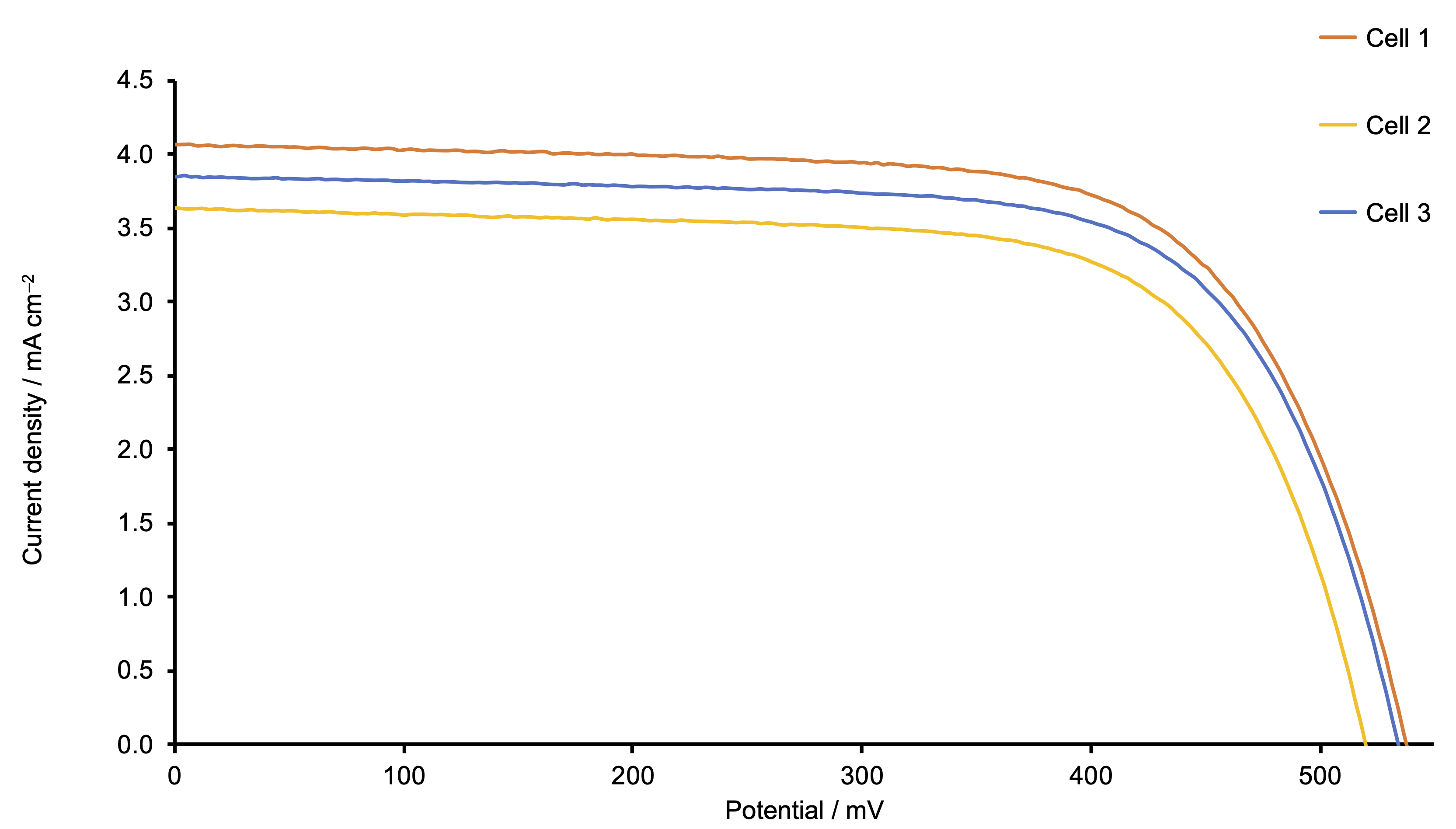


Figure S37. *J–V* curves for triplicate DSCs sensitized with [Cu(**6**)(**1**)]^+^. Measurements were made on the day of sealing the DSCs.


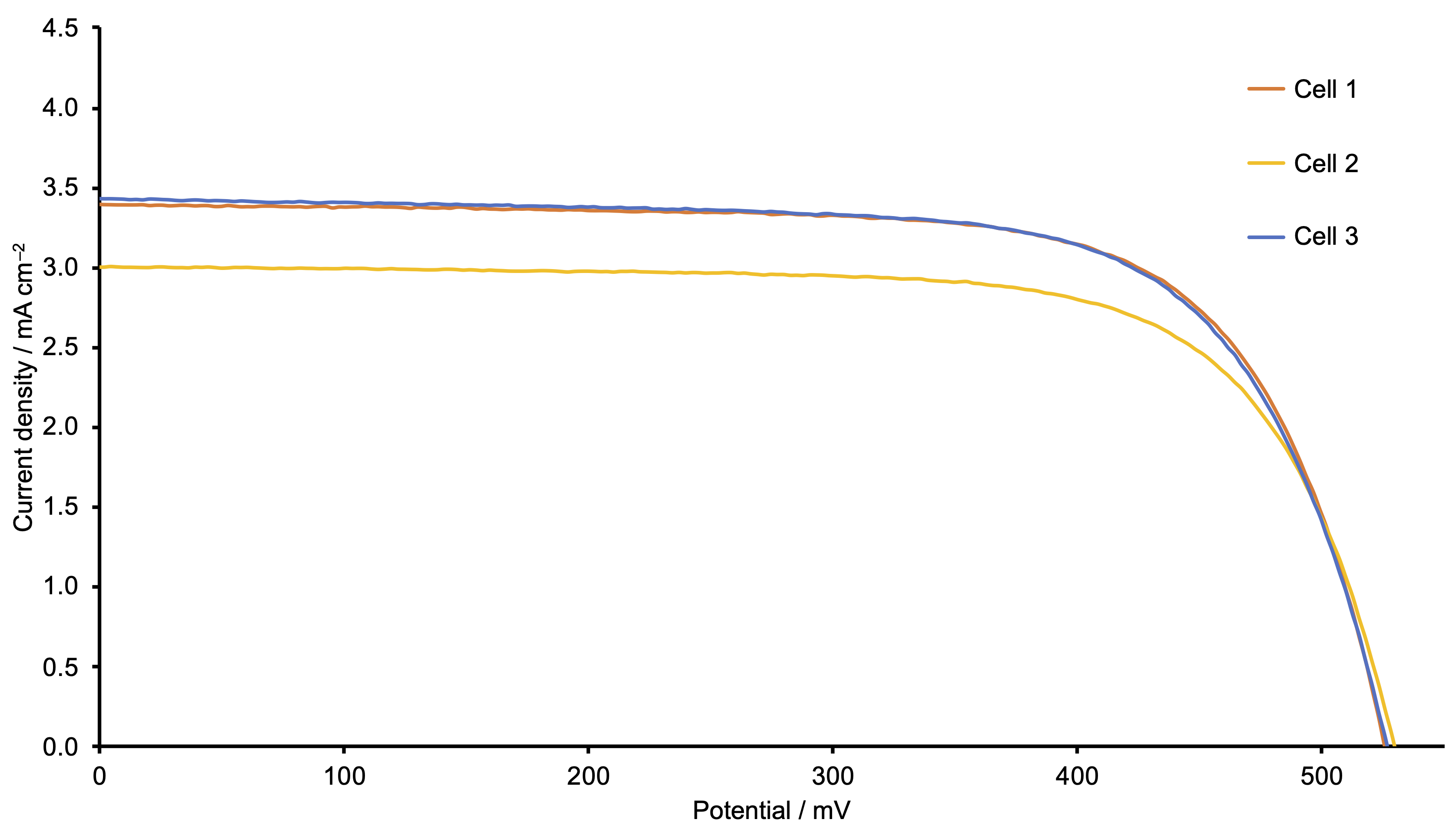


Figure S38. *J–V* curves for triplicate DSCs sensitized with [Cu(**6**)(**2**)]^+^. Measurements were made on the day of sealing the DSCs.


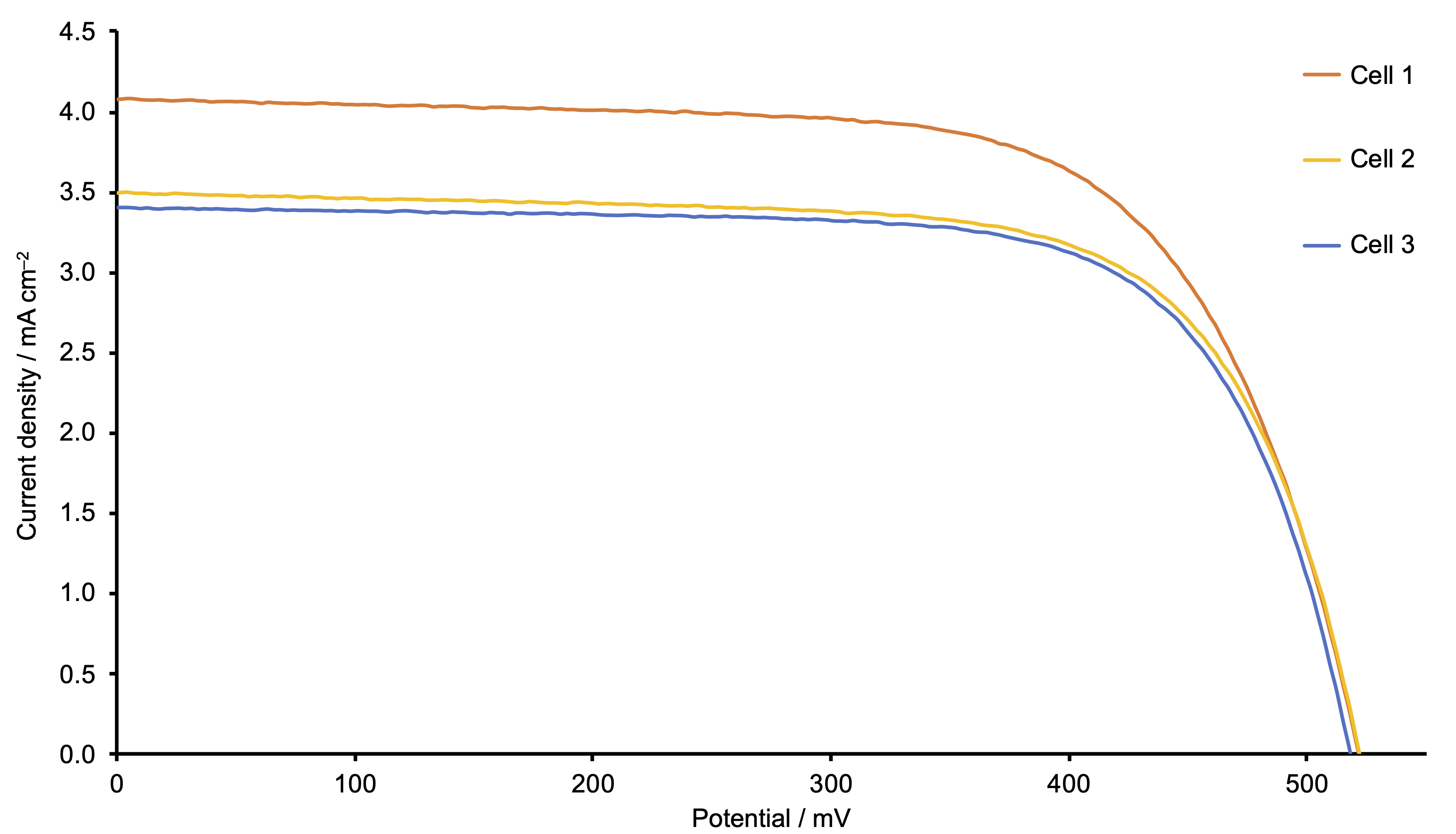


Figure S39. *J–V* curves for triplicate DSCs sensitized with [Cu(**6**)(**3**)]^+^. Measurements were made on the day of sealing the DSCs.


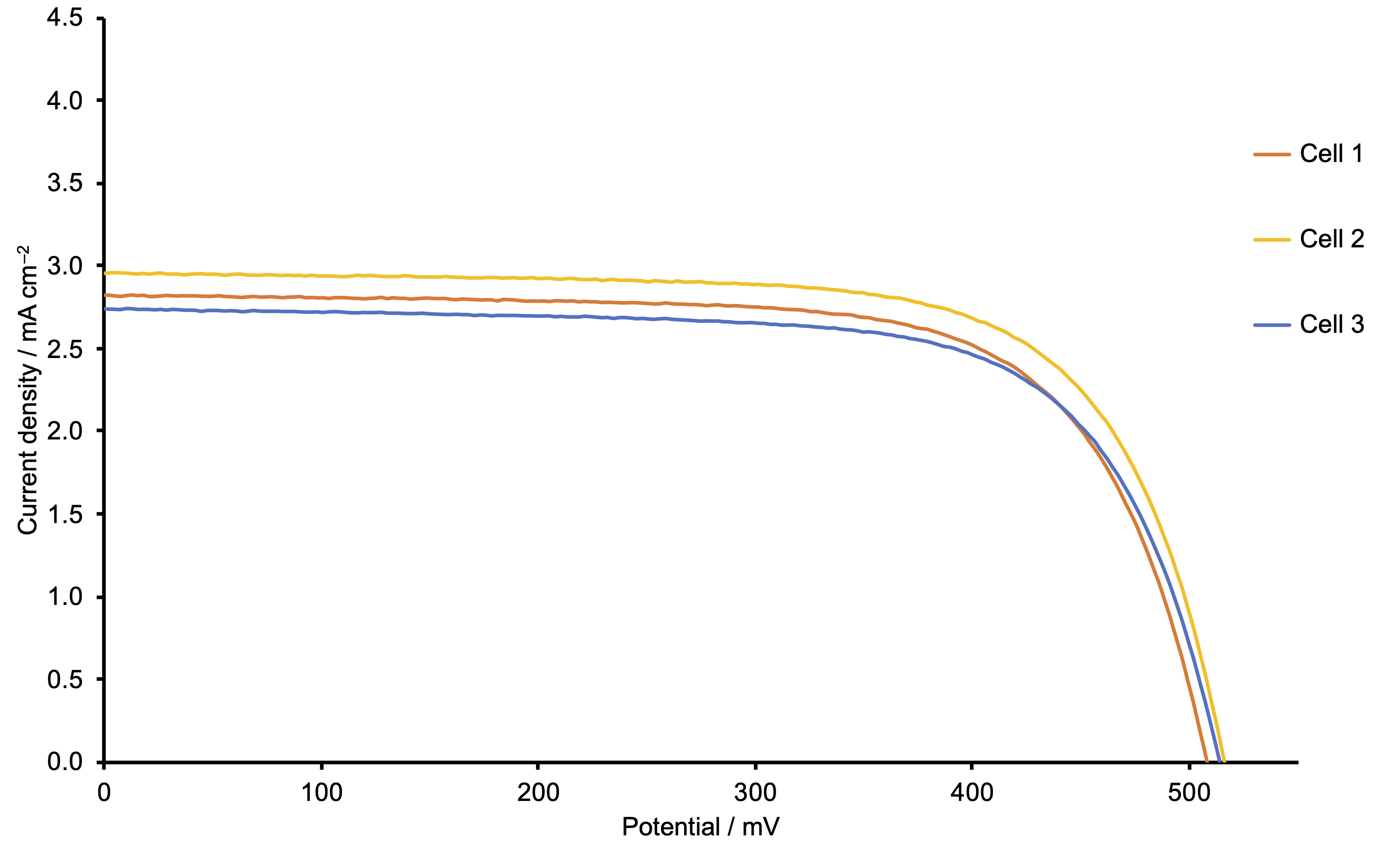


Figure S40. *J–V* curves for triplicate DSCs sensitized with [Cu(**6**)(**4**)]^+^. Measurements were made on the day of sealing the DSCs.


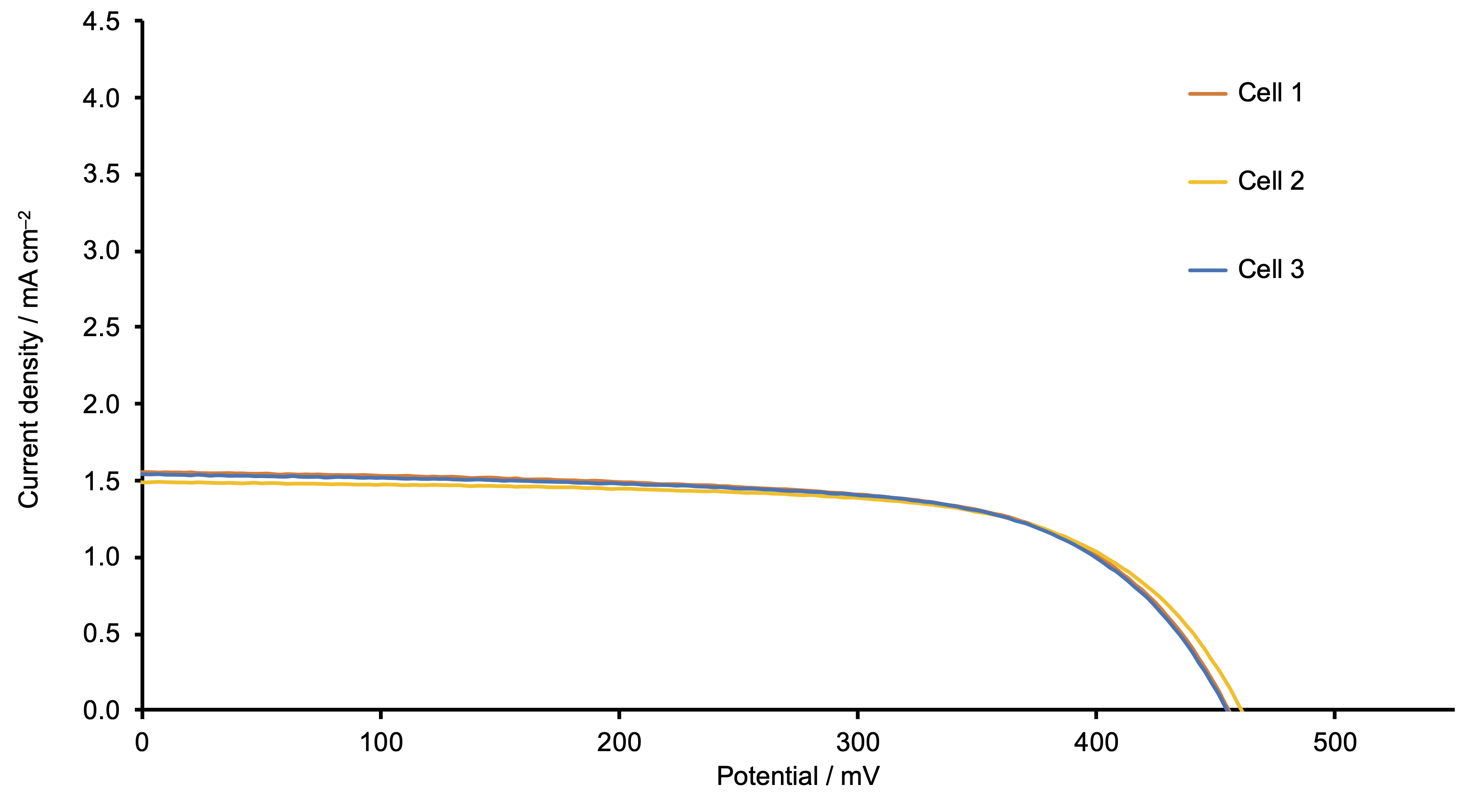


Figure S41. *J–V* curves for triplicate DSCs sensitized with [Cu(**6**)(**5**)]^+^. Measurements were made on the day of sealing the DSCs.


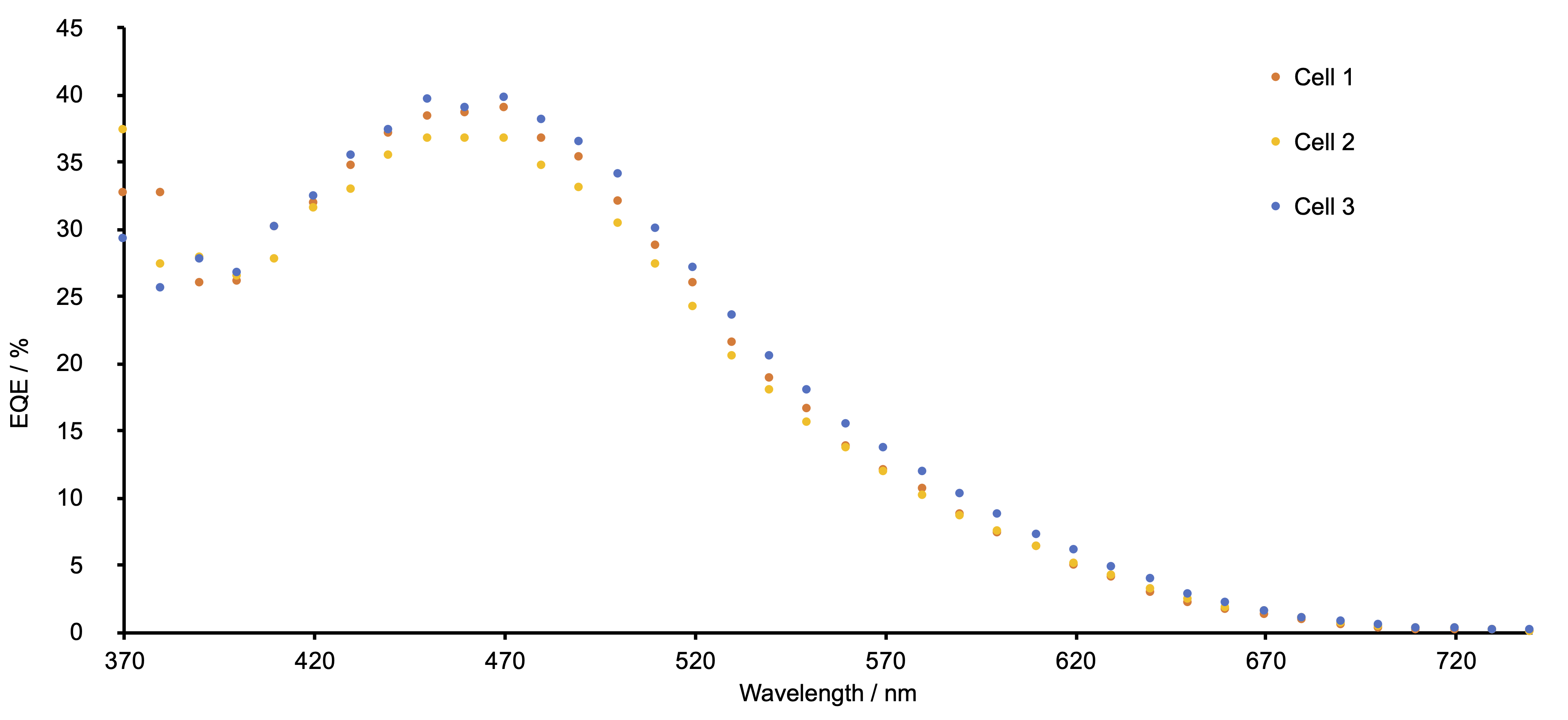


Figure S42. EQE spectra for triplicate DSCs sensitized with [Cu(**6**)(**1**)]^+^. Measurements were made on the day of sealing the DSCs.


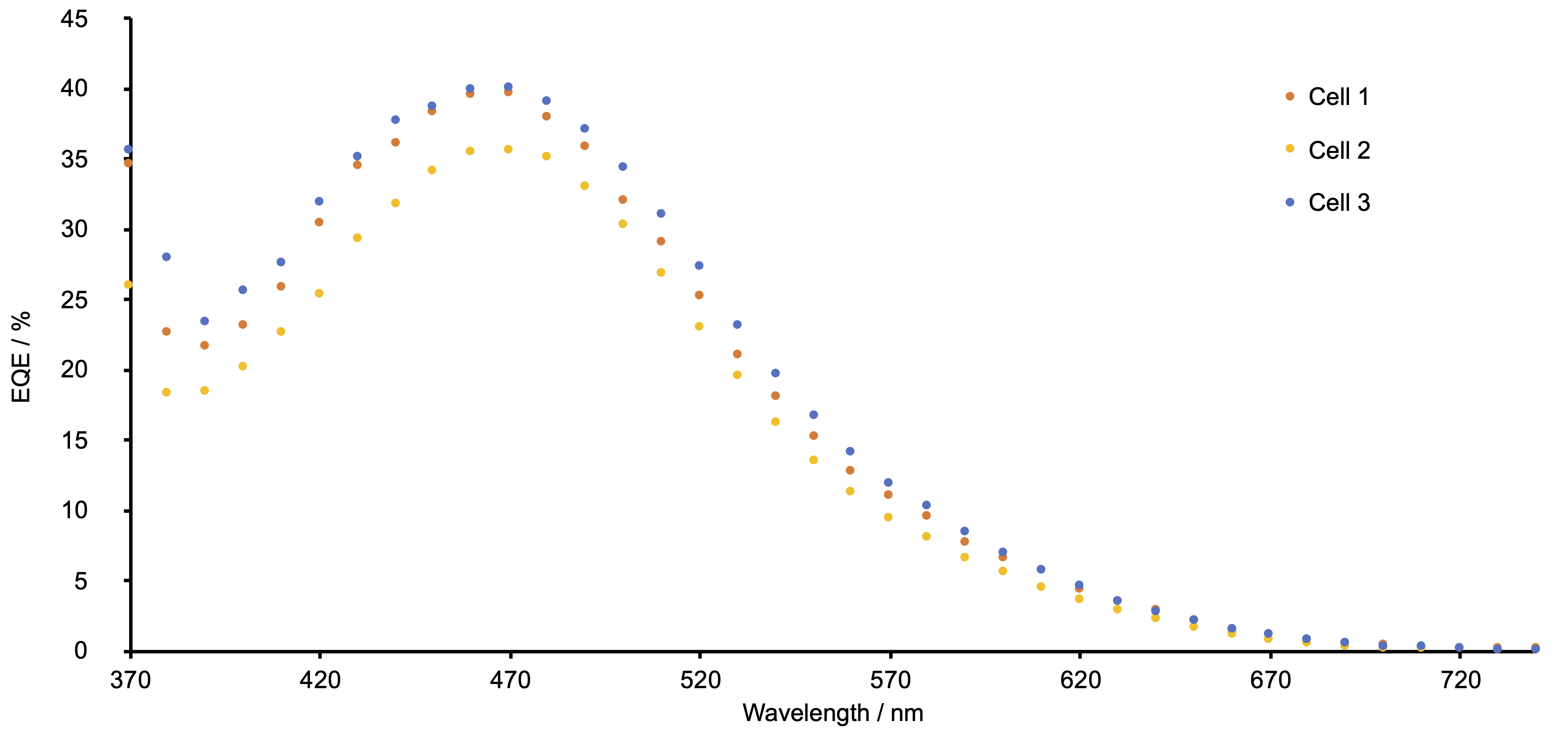


Figure S43. EQE spectra for triplicate DSCs sensitized with [Cu(**6**)(**2**)]^+^. Measurements were made on the day of sealing the DSCs.


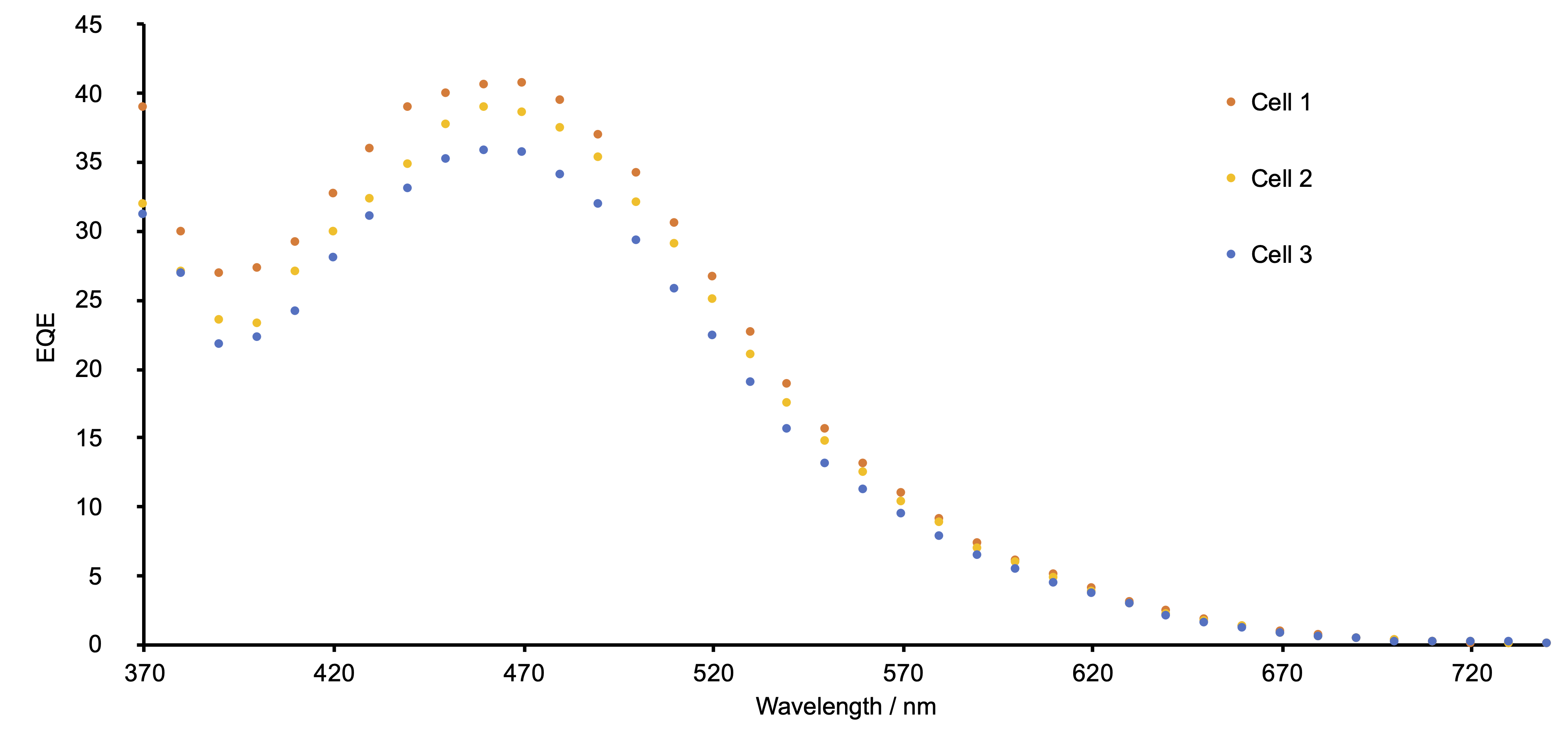


Figure S44. EQE spectra for triplicate DSCs sensitized with [Cu(**6**)(**3**)]^+^. Measurements were made on the day of sealing the DSCs.


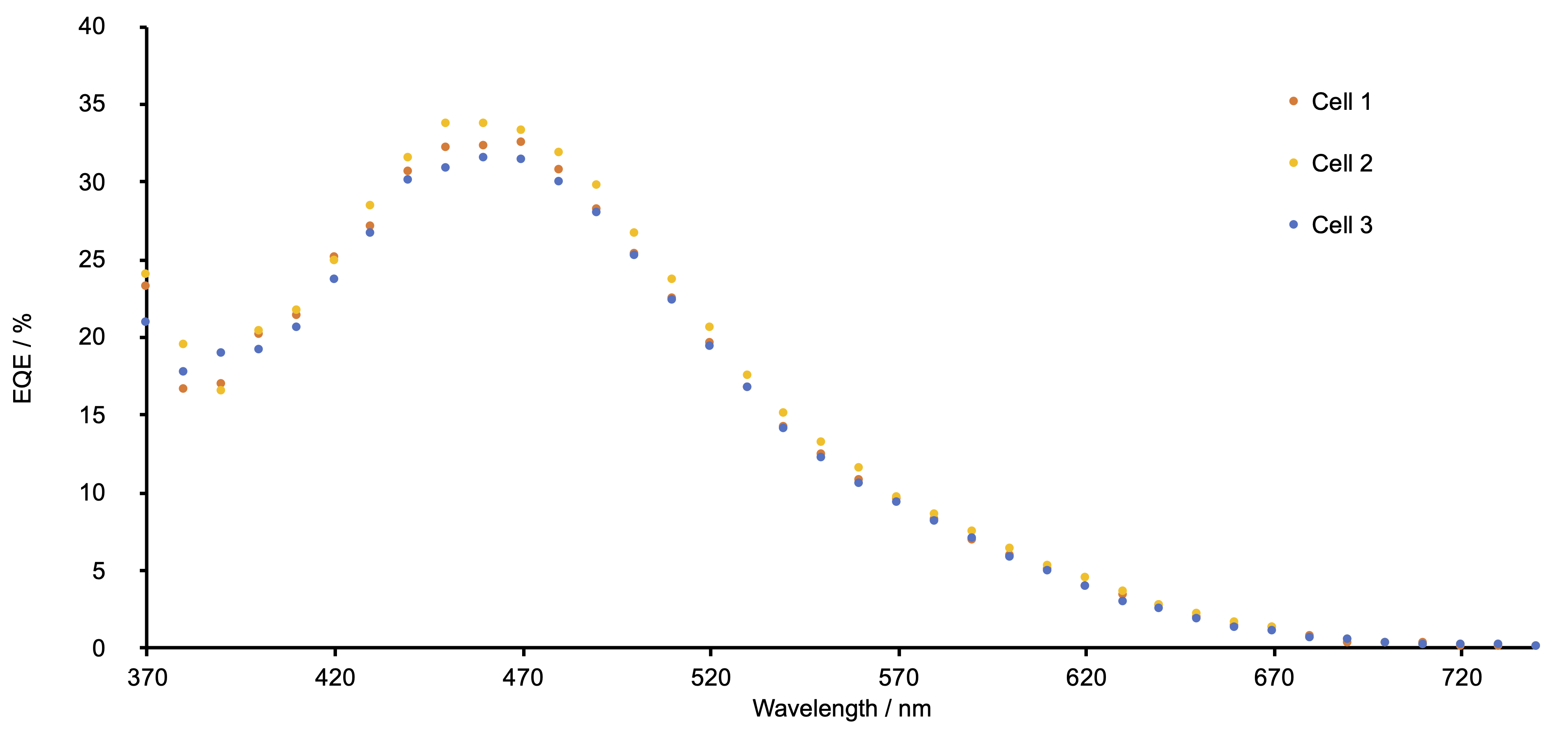


Figure S45. EQE spectra for triplicate DSCs sensitized with [Cu(**6**)(**4**)]^+^. Measurements were made on the day of sealing the DSCs.


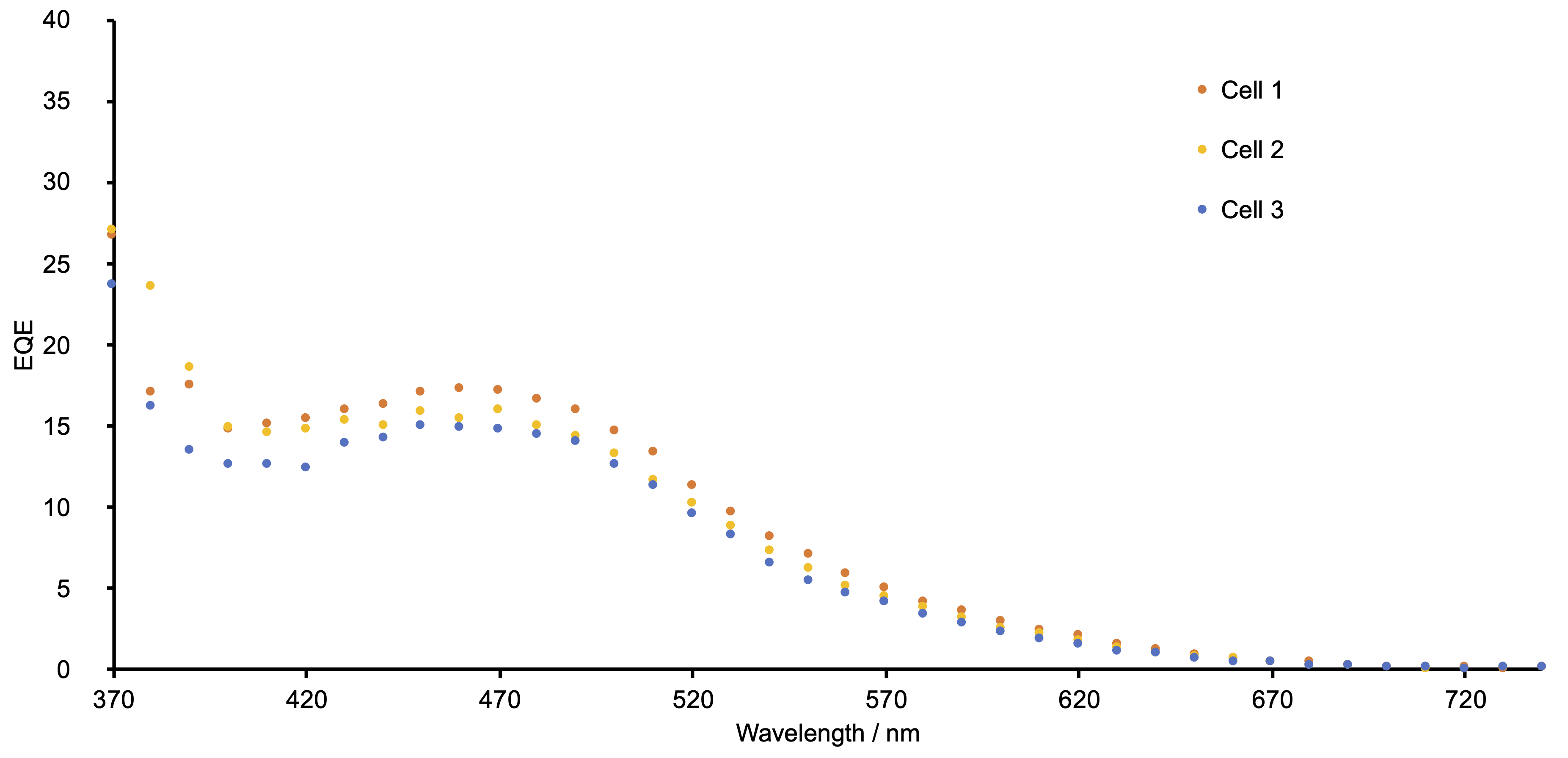


Figure S46. EQE spectra for triplicate DSCs sensitized with [Cu(**6**)(**5**)]^+^. Measurements were made on the day of sealing the DSCs.
